# Peptides inhibiting the assembly of monomeric human l‐lactate dehydrogenase into catalytically active homotetramer decrease the synthesis of lactate in cultured cells

**DOI:** 10.1002/pro.5161

**Published:** 2024-09-14

**Authors:** Alessandra Stefan, Luca Gentilucci, Francesca Ruffolo, Valentina Rossi, Sofia Sordi, Tingting He, Giuseppina di Stefano, Federica Santino, Maurizio Brigotti, Claudia Scotti, Luisa Iamele, Hugo de Jonge, Fabrizio Dal Piaz, Danilo Rocco Santarcangelo, Alejandro Hochkoeppler

**Affiliations:** ^1^ Department of Pharmacy and Biotechnology University of Bologna Bologna Italy; ^2^ CSGI, University of Firenze Sesto Fiorentino Italy; ^3^ Department of Chemistry “Giacomo Ciamician” University of Bologna Bologna Italy; ^4^ Department of Medical and Surgical Sciences University of Bologna Bologna Italy; ^5^ Department of Molecular Medicine University of Pavia Pavia Italy; ^6^ Department of Medicine University of Salerno Fisciano Italy

**Keywords:** human lactate dehydrogenase A, inhibition of assembly, monomer, peptides, subunit–subunit interaction

## Abstract

The energetic metabolism of cancer cells relies on a substantial commitment of pyruvate to the catalytic action of lactate‐generating dehydrogenases. This coupling mainly depends on lactate dehydrogenase A (LDH‐A), which is overexpressed in different types of cancers, and therefore represents an appealing therapeutic target. Taking into account that the activity of LDHs is exclusively exerted by their tetrameric forms, it was recently shown that peptides perturbing the monomers‐to‐tetramer assembly inhibit human LDH‐A (hLDH‐A). However, to identify these peptides, tetrameric hLDH‐A was transiently exposed to strongly acidic conditions inducing its dissociation into monomers, which were tested as a target for peptides at low pH. Nevertheless, the availability of native monomeric hLDH‐A would allow performing similar screenings under physiological conditions. Here we report on the unprecedented isolation of recombinant monomeric hLDH‐A at neutral pH, and on its use to identify peptides inhibiting the assembly of the tetrameric enzyme. Remarkably, the GQNGISDL octapeptide, mimicking the 296–303 portion of hLDH‐A C‐terminal region, was observed to effectively inhibit the target enzyme. Moreover, by dissecting the action of this octapeptide, the cGQND cyclic tetrapeptide was found to act as the parental compound. Furthermore, we performed assays using MCF7 and BxPC3 cultured cells, exclusively expressing hLDH‐A and hLDH‐B, respectively. By means of these assays we detected a selective action of linear and cyclic GQND tetrapeptides, inhibiting lactate secretion in MCF7 cells only. Overall, our observations suggest that peptides mimicking the C‐terminal region of hLDH‐A effectively interfere with protein–protein interactions responsible for the assembly of the tetrameric enzyme.

## 1 INTRODUCTION

Lactate dehydrogenases are stereospecific enzymes, exclusively generating d‐ or l‐lactate at the expense of pyruvate (Dennis & Kaplan, 1960). In particular, the repertoire of bacterial lactate dehydrogenases (LDHs) contains representatives featuring dimeric or tetrameric quaternary structure, and relying on β‐NADH or FAD as the redox cofactor (Garvie, 1980; Steinbüchel & Schlegel, 1983). The heterogeneity of prokaryotic LDHs does also concern their catalytic action, which is exerted according to Michaelis–Menten or cooperative kinetics (Arai et al., 2009; Garvie, 1980), with further complexity conferred to these enzymes by homotropic or heterotropic activation (e.g., by pyruvate and fructose 1,6‐bisphosphate, respectively). Remarkably, the structural and functional divergence observed for bacterial LDHs is essentially mirrored when the eukaryotic counterparts are considered. Indeed, LDHs isolated from different eukaryotic sources were reported to be assembled as dimers or tetramers (Kaplan et al., 1956; Kaplan et al., 1960; Selander & Yang, 1970; Takenaka & Schwert, 1956), and to be assisted by β‐NADH or FAD to exert their catalytic action (Cristescu et al., 2008; Eventoff et al., 1977). Quite intriguingly, the occurrence of allosteric transitions in lactate dehydrogenases expressed by eukaryotic vertebrates was early reported (Fritz, 1965), and it was recently interpreted as the outcome of dissociation‐association events affecting the tetrameric assembly of rabbit and human LDH (Iacovino et al., 2022; Pasti et al., 2022).

Concerning human lactate dehydrogenases, different homotetrameric forms were identified and studied: (i) the M form (also denoted as LDH5, and featuring the LDH‐A subunit), prevailing in muscles subjected to transient hypoxic conditions, and accordingly engaged in the reduction of pyruvate to l‐lactate (Valvona et al., 2016; Woodford et al., 2020); (ii) the H form (also known as LDH1, and containing the LDH‐B subunit), mainly expressed in aerobic tissues, where it is supposed to primarily catalyze the oxidation of l‐lactate (Read et al., 2001); (iii) the X form (LDH‐C), detected in mitochondria isolated from spermatozoa (Evrev et al., 1970; LeVan & Goldberg, 1991). In addition to the enzymes responsible for the interconversion of l‐lactate and pyruvate, the occurrence in human mitochondria of a d‐lactate dehydrogenase was recently shown (De Bari et al., 2013). Nevertheless, it is important to note that LDH‐5 and LDH‐1 represent the forms of human lactate dehydrogenase to which major attention was paid so far, both structurally and functionally speaking. Importantly enough, the structural analyses performed on the quaternary structure of LDH‐5 and LDH‐1 (Read et al., 2001) provide the necessary information to design inhibitors targeting the subunit–subunit interactions occurring in these enzymes. As will be later mentioned, this strategy represents an attractive alternative to conventional competitive inhibitors, the selectivity of which is questioned when dealing with dehydrogenases, that is, with enzymes sharing structural homology among their active sites.

Lactate is of primary importance for the energetic metabolism under conditions limiting oxidative phosphorylation, for example, in cells facing transient hypoxia. However, it was shown that lactate can be produced in fully aerobic tissues (Brooks, 1985; Schurr & Payne, 2007), and it was therefore proposed that glycolysis does always lead to the generation of lactate (Rogatzki et al., 2015). Indeed, the cytosolic lactate produced under fully aerobic conditions is transported to the mitochondrial intermembrane space, where it is oxidized to pyruvate, which is subsequently used by the Krebs cycle (Rogatzki et al., 2015). Therefore, any condition limiting the oxidative catabolism of pyruvate (not necessarily oxygen limitation) would translate into an increase of lactate level, a state which is known to take place in cancer cells (Warburg, 1956). Remarkably, a decrease of intracellular pH (pH_i_) is usually observed when the energetic metabolism is dominated by the glycolytic generation of lactate. It should however be noted that the generation of lactate by LDH does not induce acidification per se, the reduction of pyruvate at the expense of NADH + H^+^ being a proton‐consuming reaction. Instead, lactic acidosis represents the outcome of a net release of H^+^ from ATP hydrolysis under hypoxic or anoxic environments (Zilva, 1978). A peculiar situation concerns cancer cells, the energetic metabolism of which is mainly driven by glycolysis and lactate release, and whose pH_i_ is nevertheless higher than in normal cells (White et al., 2017). Remarkably, this phenotype of cancer cells avoids the dissociation of tetrameric LDH‐A, thereby preserving its catalytic action (Pasti et al., 2022).

An interesting link between the quaternary structure of LDH‐A and its relevance in the energetic metabolism of cancer cells has recently been reported (Fan et al., 2011). In particular, it was shown that tyrosine 10 (Y10) of LDH‐A is specifically phosphorylated in quite a number of human cancer cell lines. Moreover, elegant size exclusion chromatography experiments revealed that the phosphorylation of Y10 favors the assembly of tetrameric LDH‐A at the expense of the enzyme monomeric and dimeric forms, the relative abundance of which is higher in preparations of LDH‐A bearing unphosphorylated Y10. Remarkably, these biochemical features do phenotypically translate in the observation that the invasive potential of different forms of malignant cells can be inhibited by suppressing the phosphorylation of LDH‐A at Y10 (Jin et al., 2017). Furthermore, it was reported that pyruvate counteracts the inhibition exerted by metformin on cancer cells proliferation (Gui et al., 2016). This effect triggered by pyruvate is linked to the action of LDH, which is necessary to maintain the NAD^+^/NADH molar ratio within values appropriate for cells growth and duplication (Gui et al., 2016).

Not surprisingly, the importance of LDH‐A in the glycolytic pathway of malignant cells prompted a wide search of potent inhibitors of this enzyme. In particular, the active site of LDH‐A represents the major target selected so far, and quite a number of competitive inhibitors mimicking pyruvate or β‐NADH were synthesized and characterized (Granchi et al., 2013; Yu et al., 2001). Albeit highly effective inhibitors belonging to this class were reported (e.g., a glycolate derivative of β‐NADH, Kotlyar et al., 2010), they face the drawback of lacking selectivity. Structurally speaking, NADH‐dependent dehydrogenases do indeed feature a rather high level of similarity, the occurrence of which constrains the landscape of the searchable competitive inhibitors exerting a selective action on a particular member of this enzymatic family. In addition, the structural similarity of the human LDH‐A and LDH‐B represents an additional concern to the use of competitive inhibitors. Accordingly, to improve the selectivity of LDH antagonists a couple of allosteric inhibitors (a phthalimide and a dibenzofuran derivative, respectively) were recently synthesized, and it was shown that their IC_50_ values against LDH‐A and LDH‐B lie in the nM and μM range, respectively (Friberg et al., 2020).

Notably, a further alternative to competitive inhibitors is represented by compounds targeting protein–protein interactions. In particular, the loss of activity related to the dissociation of tetrameric LDH‐A (Hermann et al., 1981; Pasti et al., 2022) prompts the search for peptides able to interfere with the quaternary structure of this enzyme. Peptides can indeed mimic a secondary structure element supposed to be a major determinant of the reciprocal interactions engaged by the subunits of an oligomeric protein (Wang et al., 2021). Nevertheless, it should be noted that the effectiveness of peptides in interfering *in vivo* with protein–protein interactions can be negatively affected by their high degree of conformational freedom and by their facile degradation in host cells. Remarkably, these complications can be overcome using conformationally restricted cyclic peptides and by including d‐amino acids in the primary structure of peptides, respectively. Therefore, peptides represent a promising tool to inhibit the assembly of enzymes that exclusively feature catalytic activity in their oligomeric state. Concerning lactate dehydrogenases, this strategy was first tested using different peptides targeting the C‐terminal region of LDH‐A (Jafary et al., 2019). According to dynamic light scattering analyses, a couple of peptides among those tested, that is, the hexamers IYNLLK and KVVYNV, were found to be competent in inducing a shift of the apparent LDH‐A molecular mass from about 240 to ca. 100 kDa (Jafary et al., 2019). Soon after, very elegant work was performed to identify peptides capable of binding human LDH‐1 with high affinity (Thabault et al., 2020). To this aim, a dimeric form of LDH‐B was used for testing the association of candidate peptides to the tetramerization site of LDH‐B, that is, to the C‐terminal region of each enzyme subunit. By these means, an interesting observation was obtained: the linear peptide ATLKEKLI was found to feature a *K*_D_ for dimeric LDH‐B equal to 1 mM, whereas the corresponding constant of cyclic derivatives of this octapeptide was found to decrease down to 25 μM (Thabault et al., 2020). Moreover, when the cyclic octapeptide best performing against dimeric LDH‐B was used to target tetrameric LDH‐A and LDH‐B, *K*_D_ values equal to 117 and 380 μM were determined, respectively. Interestingly, an additional tetramerization site of LDH‐B (corresponding to residues located in the E62‐F72 region of the primary structure) was identified, and a hexadecapeptide peptide directed against this target was observed to bind dimeric LDH‐B with a *K*_D_ equal to 240 μM (Thabault et al., 2021). Quite intriguingly, the cyclic octapeptide and the linear hexadecapeptide share the capability of inducing the dissociation of tetrameric LDH‐A, according to EC_50_ values equal to 172 and 262 μM, respectively. Remarkably, it was reported that peptides appropriately designed are proficient in inhibiting the activity of human LDH‐A (Nadal‐Bufi et al., 2021). In particular, a heptadecapeptide (denoted cGmC9) consisting of two β‐strands connected by two loops was shown to inhibit the reduction of pyruvate catalyzed by LDH‐5, and the IC_50_ value for this antagonist action was determined as equal to 2.5 μM. Importantly enough, the cGmC9 peptide inhibits LDH‐5 independently of substrate (pyruvate) concentration, therefore ruling out a competitive mechanism for this peptide. It should however be noted that the inhibition of LDH‐5 by the cGmC9 peptide was assayed using the tetrameric enzyme exposed to pH 2.5 (in order to dissociate its subunits and let the peptide bind them under these conditions), and thereafter shifting the pH to 7.4. This procedure, besides being efficient, faces the inconvenient of exposing the target enzyme to harsh pH conditions, under which the peptide‐enzyme association is also tested.

To provide a useful tool for screening the capability of candidate peptides to inhibit LDH‐A activity, we considered of interest to attempt the isolation of homogeneous monomeric LDH‐A, the availability of which would greatly improve the robustness of assays designed to test the assembly of LDH‐5. In addition, to analyze in detail how the competence of peptides in inhibiting the assembly and the catalytic activity of LDH‐A is affected by their structure, we synthesized quite a number of linear and cyclic peptides, containing or not d‐amino acids. Accordingly, we report here on a simple procedure to obtain purified human LDH‐A monomers, along with the synthesis and characterization of an ensemble of peptides efficiently targeting this enzyme *in vitro* and in cultured cells. The implications of our observations on the use of peptides to interfere with the assembly of oligomeric proteins are also discussed.

## 2 RESULTS

### 2.1 Overexpression of human LDH‐A and purification of its monomeric form

**FIGURE 1:**
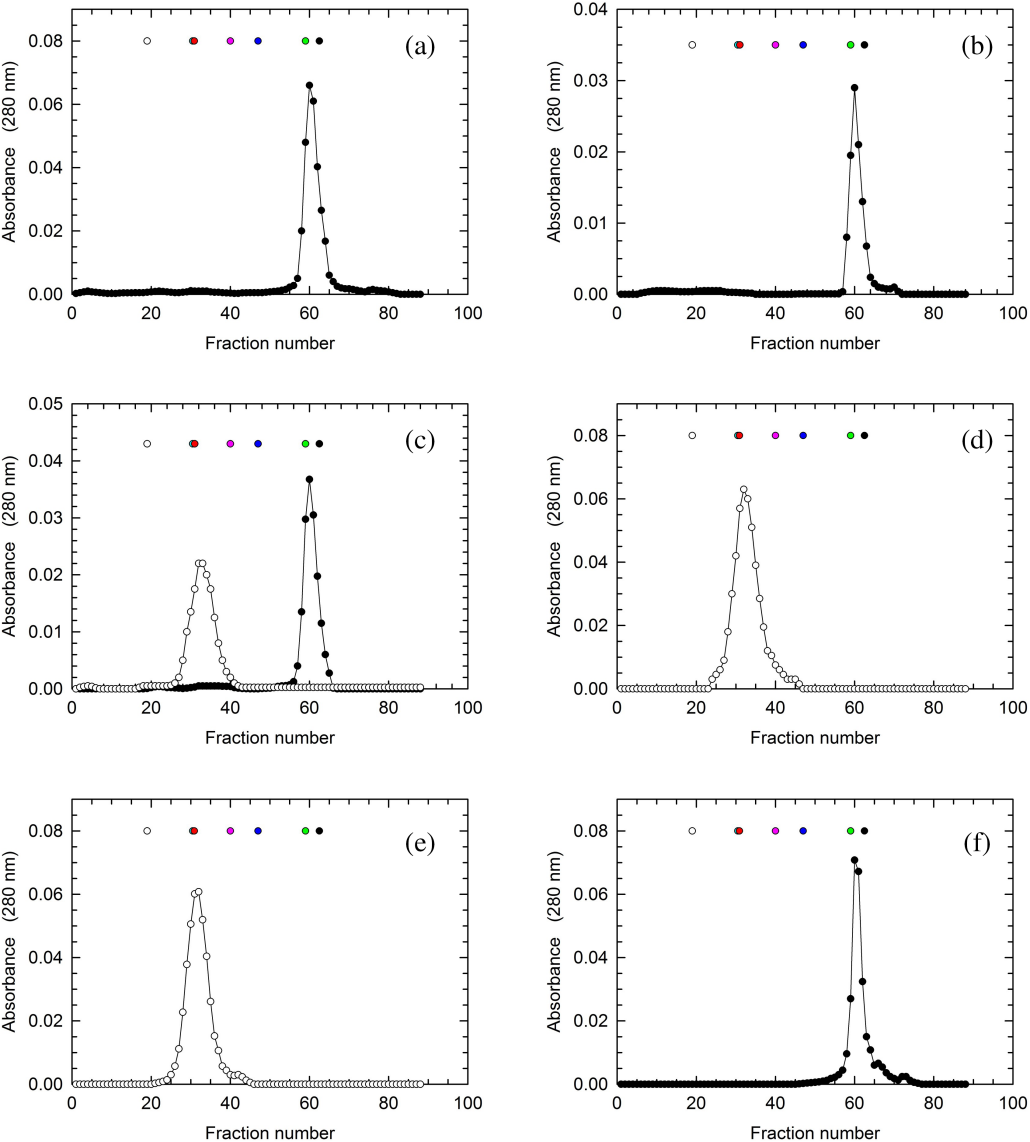
Isolation of monomeric and tetrameric human LDH‐A. Gel filtration of human LDH‐A previously subjected to affinity and hydrophobic interaction chromatography. The gel filtration chromatography was performed using: (a) 10 mM Tris–HCl; (b) 10 mM HEPES; (c) 10 mM Tris–HCl (black circles) or 10 mM Tris–HCl supplemented with 125 μM β‐NADH and 5 mM oxamate (white circles); (d) 10 mM HEPES, 150 mM NaCl; (e) 10 mM HEPES, 150 mM NaCl; (f) 10 mM Tris–HCl. A Superdex 200 column was used throughout, and all buffers were poised at pH 7.5. The white, cyan, red, magenta, blue, green, and black circles indicate the elution volume of ferritin, catalase, aldolase, albumin, ovalbumin, chymotrypsinogen, and RNase A, respectively. The absorption spectra of these molecular mass markers dissolved in Tris–HCl or HEPES buffer are reported in Figure S3.

To attempt the overexpression of human LDH‐A in *Escherichia coli*, we used a synthetic gene optimized for the codon usage of the bacterial host (Figure S1, see also Section 5). Notably, protein extracts obtained from cells overexpressing the synthetic gene were found to contain soluble LDH‐A, the purification of which was pursued with standard chromatographic methods (see Section 5). In particular, the first purification step was carried out with a Cibacron Blue column, and further separation from contaminants was obtained by means of hydrophobic interaction chromatography. Remarkably, the purification level detected after these two chromatographic steps was rather satisfactory (Figure S2). Nevertheless, we decided to achieve further purification with the aid of gel filtration chromatography. To this aim, we used a Superdex 200 column, conditioned with 10 mM Tris–HCl, pH 7.5. Surprisingly, we were unable to detect any significant amount of tetrameric LDH‐A in the eluted fractions, whereas a considerable amount of the enzyme in monomeric form was recovered from the column (Figure 1a). Moreover, it should be noted that the molecular mass calculated from the observed elution volume of LDH‐A was determined as equal to 19.8 kDa, a value significantly lower than that expected (36.6 kDa). To test if the isolation of monomeric LDH‐A was due to the particular conditions selected for gel filtration, we prepared a second batch of enzyme. This was accomplished according to the procedure previously mentioned, except for the size exclusion chromatography, which was performed using 10 mM HEPES, pH 7.5. Quite intriguingly, monomeric LDH‐A was again obtained, indicating that the output of the gel filtration chromatography is not affected by the cationic or anionic nature of the buffer selected for this purification step (Figure 1b). However, when the Superdex 200 column was conditioned with 10 mM Tris–HCl (pH 7.5) supplemented with 125 μM β‐NADH and 5 mM oxamate (a competitive inhibitor of lactate dehydrogenases, structurally similar to pyruvate), we recovered tetrameric LDH‐A, the molecular mass of which was estimated equal to 157 kDa (Figure 1c). In addition, a very similar output was observed when 10 mM HEPES, 150 mM NaCl (pH 7.5) was used for the size exclusion chromatography, with the peak containing tetrameric LDH‐A corresponding to a molecular mass equal to 198 kDa (Figure 1d). Finally, we tested if the addition of NaCl to previously purified monomeric LDH‐A triggers its assembly into the corresponding tetramer. Interestingly, when 150 mM NaCl was supplemented to monomeric LDH‐A and the enzyme solution accordingly obtained was loaded onto the Superdex 200 column conditioned with 10 mM Tris–HCl (pH 7.5) containing 150 mM NaCl, tetrameric LDH‐A was obtained (Figure 1e). Moreover, when the tetramer eluted by means of this size exclusion chromatography was loaded onto the Superdex 200 column conditioned with 10 mM Tris HCl devoid of NaCl, LDH‐A was eluted in monomeric form (Figure 1f). Therefore, the purification strategy reported here for human LDH‐A is useful to isolate this enzyme in tetrameric or in monomeric form (yielding ca. 10 mg/L of purified enzyme), with the selection between these two forms depending only on the type of buffer used to perform gel filtration chromatography.

**FIGURE 2:**
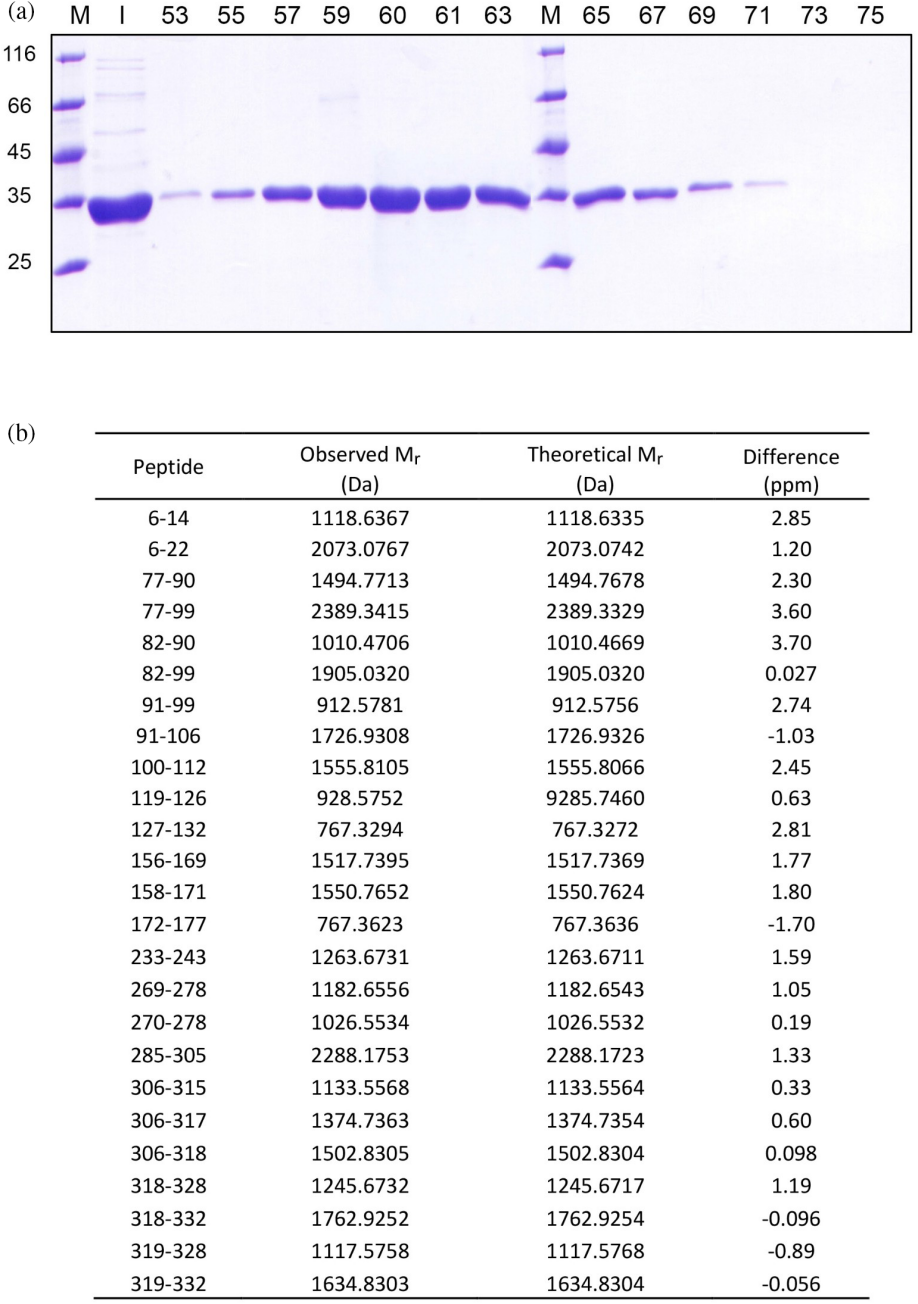
Analysis of monomeric human LDH‐A by SDS‐PAGE and mass spectrometry. (a) Electrophoretic analysis of fractions eluted from the Superdex 200 column equilibrated with 10 mM Tris–HCl (pH 7.5) and used to perform the last purification step of monomeric LDH‐A. M: Molecular mass markers (their *M*_*r*_ is indicated in kDa at the left); I: Input; the fraction numbers are indicated on the top. (b) Identification by mass spectrometry of peptides obtained by in‐gel tryptic digestion of monomeric human LDH‐A.

Considering that the deletion of few amino acids (e.g., 20) from the N‐terminal of LDH‐A is responsible for the dissociation of the tetrameric enzyme into dimers (Girg et al., 1981; Girg et al., 1983), we analyzed by mass spectrometry one of the fractions eluted from the Superdex 200 column that contained monomeric LDH‐A (Figure 2a). Importantly enough, by these means we were able to detect peptides that covered most of the enzyme primary structure (from Asp6 to Lys332, Figure 2b), indicating that the purification procedure reported here implies the isolation of full‐length monomeric LDH‐A.

### 2.2 Structural properties of monomeric LDH‐A

**FIGURE 3:**
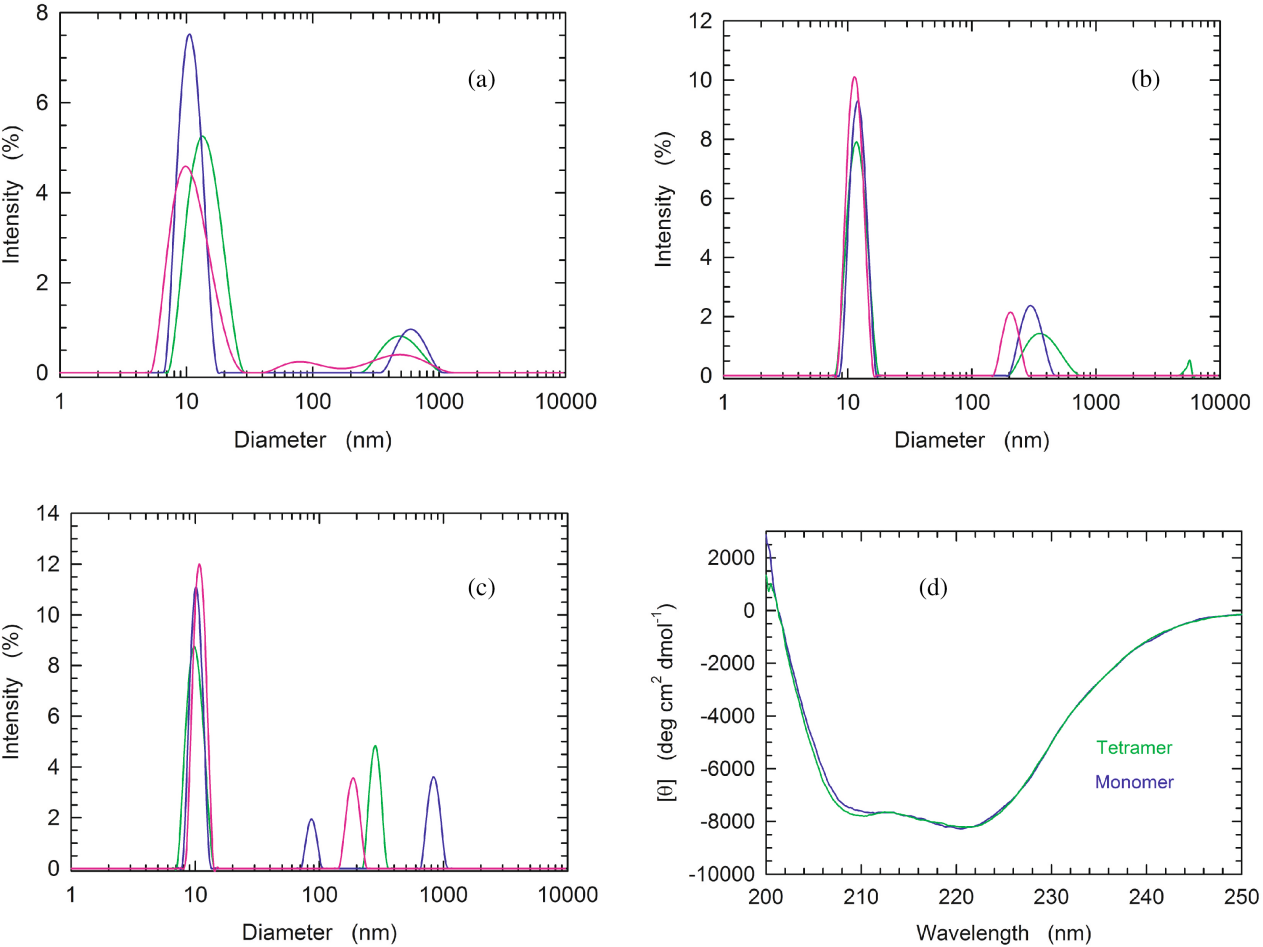
Analysis of the quaternary and secondary structure of human LDH‐A. (a–c) Dynamic light scattering experiments performed with monomeric LDH‐A in: (a) 10 mM Tris–HCl, (b) 10 mM Tris–HCl supplemented with 125 μM β‐NADH, (c) 10 mM Tris–HCl containing 125 μM β‐NADH and 10 mM oxamate. All buffers were poised at pH 7.5. (d) Far‐UV CD spectra of tetrameric and monomeric LDH‐A (green and blue line, respectively), in PBS buffer.

Having observed that β‐NADH and oxamate are able to promote the tetramerization of LDH‐A monomers, we considered of interest to characterize, by Dynamic Light Scattering (DLS), the association of the enzyme subunits. First, we analyzed the distribution of particles using monomeric LDH‐A in 10 mM Tris–HCl, pH 7.5. Notably, when three consecutive DLS assays were carried out with this sample the observed peaks were indicative of a considerable polydispersity of the detected particles (Figure 3a). Moreover, the size distribution of LDH‐A particles revealed quite important differences among the three assays, suggesting a considerable spreading over a wide conformational landscape (Figure 3a). In contrast, the addition of 125 μM β‐NADH to monomeric LDH‐A was found to translate into a monodisperse sample (Figure 3b). Furthermore, no major differences were observed among the outputs generated by three successive DLS assays (Figure 3b). In particular, under these conditions the molecular mass of LDH‐A was determined as equal to 212 ± 35, 212 ± 31, and 190 ± 27 kDa, respectively, yielding a mean value equal to 205 ± 13 kDa. Remarkably, when both 125 μM β‐NADH and 10 mM oxamate were added to monomeric LDH‐A, the DLS peaks observed with three consecutive analyses featured high reciprocal similarity, and are indicative of a monodisperse sample (Figure 3c). Under these conditions, the values of molecular mass of LDH‐A were determined as equal to 153 ± 9, 153 ± 14, and 138 ± 16 kDa, respectively, yielding a mean value of 148 ± 9 kDa, in excellent agreement with an expected value for tetrameric LDH‐A equal to 146.3 kDa.

To inspect the secondary structure of monomeric LDH‐A, we compared its circular dichroism (CD) spectrum with that of the tetrameric enzyme. It should be noted that both spectra were obtained in PBS buffer, containing 137 mM NaCl. Therefore, the ionic strength of the buffer should induce the association of LDH‐A monomers into the corresponding tetramer (cf. Figure 1d,e). Interestingly, no major differences were detected between the two samples, with the only exception being the amplitude of molar ellipticity at 208 nm (Figure 3d).

### 2.3 Binding of β‐NADH by tetrameric and monomeric LDH‐A

**FIGURE 4:**
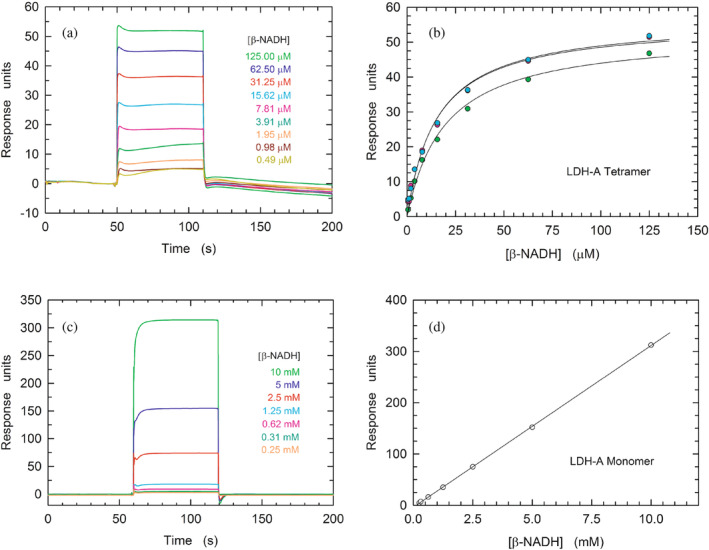
Binding of β‐NADH to tetrameric and monomeric hLDH‐A. (a) Kinetics of the association of β‐NADH to tetrameric hLDH‐A as detected by surface plasmon resonance. Sensorgrams were observed loading increasing concentration of β‐NADH (0.49–125 μM) on a sensor chip modified with immobilized tetrameric human LDH‐A. (b) Determination of the *K*_D_ of hLDH‐A for β‐NADH. The response units refer to the data shown in “a.” The continuous lines represent the best fit of a parametric rectangular hyperbola to the experimental observations. (c) Sensorgrams observed loading increasing concentration of β‐NADH (0.25–10 mM) on a sensor chip modified with immobilized monomeric human LDH‐A. (d) Linear dependence of the response units reported in “c” on the concentration of loaded β‐NADH. The continuous line represents the best fit of a linear equation to the experimental observations.

To further inspect the biochemical features of monomeric LDH‐A, we analyzed the binding of β‐NADH to LDH‐A (at pH 7.5) by Surface Plasmon Resonance (SPR). First, we tested the association between the tetrameric enzyme and the redox cofactor. Unfortunately, both the binding of β‐NADH to tetrameric LDH‐A and the subsequent dissociation of the binary complex were found to obey a very fast kinetics, whose rate constants could not be determined (Figure 4a). Nevertheless, our observations were useful to determine the *K*_D_ of the enzyme–cofactor complex, the value of which was evaluated by means of three independent assays as equal to 28, 22, and 21 μM, yielding a mean value equal to (23 ± 4)•10^−6^ M (Figure 4b). Surprisingly, when the binding of β‐NADH to monomeric LDH‐A was tested, the association of the redox cofactor to the monomeric enzyme featured a much lower amplitude than that detected in the presence of tetrameric hLDH‐A (Figure S4, cf. with Figure 4a). However, by decreasing to 7.0 the pH of the binding assay, the observed amplitude was more than twice the corresponding response at pH 7.5 (Figure S4). Moreover, a further assay performed at pH 7.0 (i.e., under the more favorable condition) revealed a linear dependence of the SPR response up to very high β‐NADH concentrations, for example, 10 mM (Figure 4c,d). Accordingly, the *K*_D_ for the monomer‐coenzyme binary complex cannot be determined, with this finding being apparently in contrast with the observations obtained by means of DLS experiments (Figure 3). However, it should be considered that the DLS experiments were carried out with free LDH‐A monomers, whereas the SPR assays were performed with immobilized enzyme which therefore is incapable to assemble into the tetrameric form. Accordingly, we propose that the binding of β‐NADH and the assembly of monomers represent a concerted action, the occurrence of which is hampered by the immobilization of LDH‐A.

### 2.4 Kinetics of pyruvate reduction by tetrameric and monomeric LDH‐A

**FIGURE 5:**
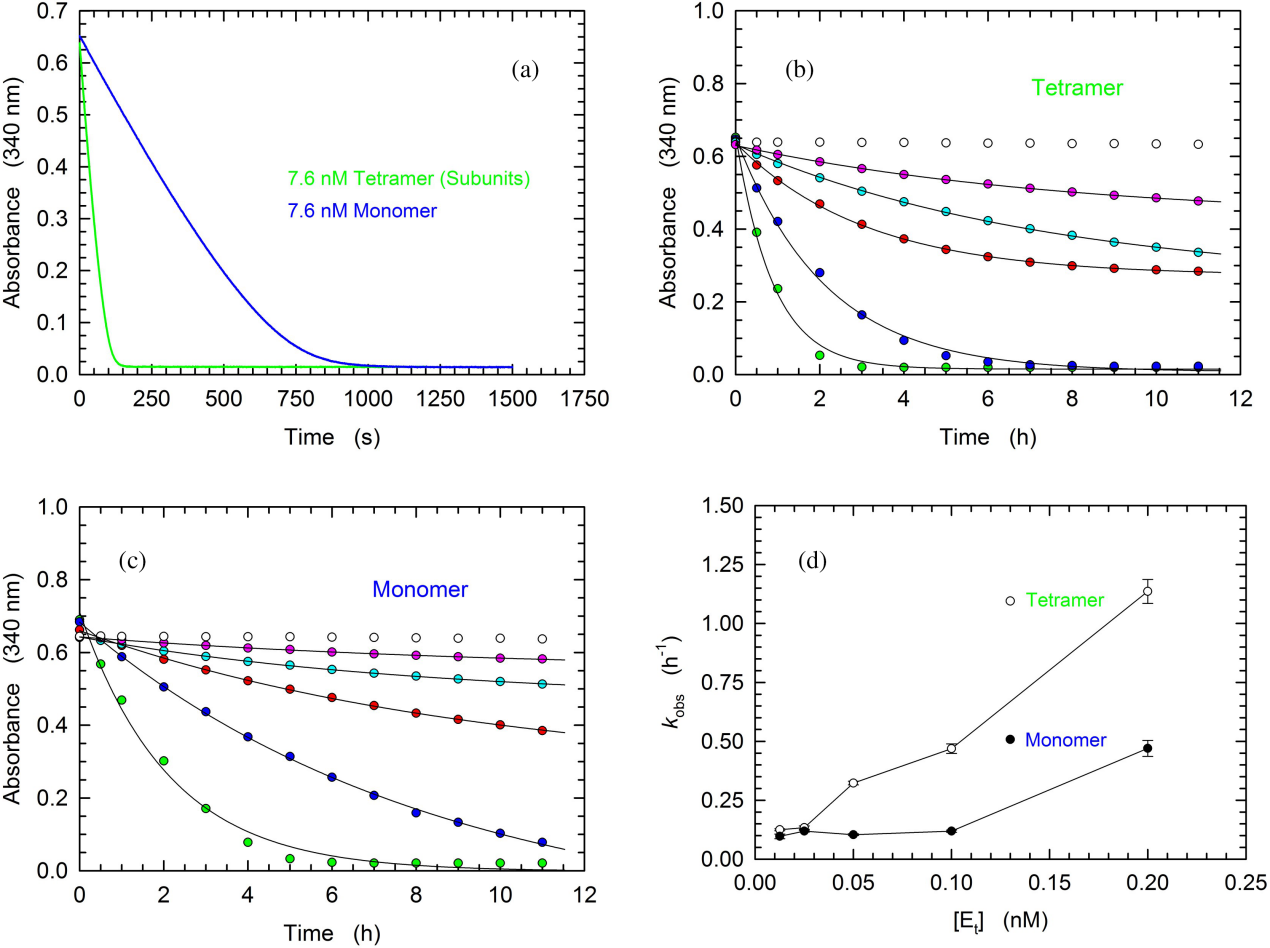
Kinetics of β‐NADH oxidation catalyzed by tetrameric or monomeric human LDH‐A. (a–c) Activity assays were performed using reaction mixtures containing 50 mM Tris–HCl (pH 7.5), 100 μM β‐NADH, and 500 μM pyruvate. (a) Kinetics of β‐NADH oxidation detected in the presence of 7.6 nM of tetrameric or monomeric LDH‐A (green and blue line, respectively). (b, c) Time‐course of β‐NADH oxidation catalyzed by 12.5, 25, 50, 100, or 200 pM tetrameric (b) or monomeric (c) human LDH‐A (magenta, cyan, red, blue, and green circles, respectively). The time‐course observed in the absence of enzyme is also reported (white circles). (d) Dependence on enzyme concentration of the rate constants determined for the reactions catalyzed by tetrameric (white circles) or monomeric LDH‐A (black circles) and reported in “b” and “c.” To obtain the *k*_obs_ values a single‐exponential equation was fitted to the experimental observations.

As previously mentioned, DLS assays revealed that the addition of β‐NADH and oxamate to monomeric LDH‐A triggers the assembly of LDH‐A monomers into the enzyme tetrameric form. Accordingly, the assembled monomers should feature catalytic competence, the extent of which would be, in turn, suggestive of monomers‐to‐tetramer conversion. Therefore, we performed a set of activity assays using reaction mixtures containing 10 mM Tris–HCl (pH 7.5), 100 μM β‐NADH, 500 μM pyruvate, and variable concentrations of LDH‐A in monomeric or tetrameric form. To prime this analysis, we compared the kinetics of β‐NADH oxidation catalyzed by tetrameric or monomeric LDH‐A, at 7.6 nM final concentration of subunits. When the tetrameric enzyme was used, we detected a very fast reaction, reaching equilibrium in about 2.5 min (Figure 5a). Conversely, when monomeric LDH‐A was used, the reaction rate was definitely slower, taking approximately 20 min to approach completion (Figure 5a). We propose that this sharp difference represents the outcome of two major factors: (i) a partial assembly of monomers into the enzyme homotetramer and (ii) during the reaction the limiting substrate (β‐NADH) is oxidized, therefore restraining the assembly of monomers. Taking into account that tetrameric LDH‐A faces a dilution‐induced dissociation, even under conditions of neutral pH (Pasti et al., 2022), further tests were carried out in the presence of much lower concentrations of enzyme. We indeed reasoned that in the presence of low enzyme concentrations the difference between the activities exerted by tetrameric LDH‐A and by assembled monomers should be less pronounced when compared to the difference observed at higher enzyme concentrations. Therefore, LDH‐A activity was assayed in the presence of concentrations of LDH‐A subunits ranging from 0.0125 to 0.2 nM. As expected, tetrameric LDH‐A outperformed the catalytic action of assembled monomers (Figure 5b,c), albeit to a lower extent compared to the difference observed in the presence of 7.6 nM enzyme (Figure 5a). Quantitatively speaking, by fitting a single exponential equation to the experimental observations larger *k*_obs_ values were determined for the LDH‐A tetramer, except under the conditions of maximal dilution (0.0125 and 0.025 nM subunits, Figure 5d).

### 2.5 Kinetic parameters of tetrameric and monomeric LDH‐A

**FIGURE 6:**
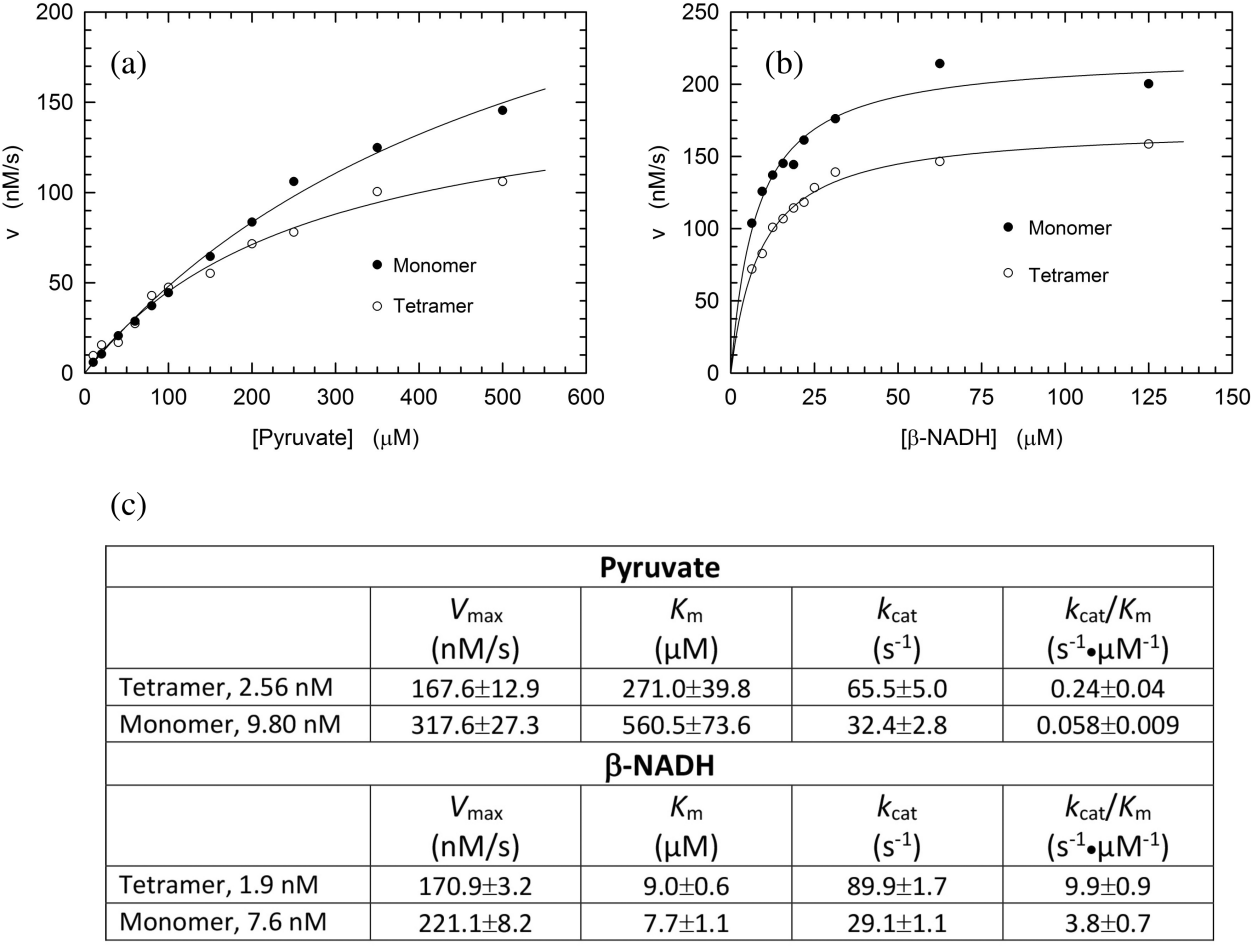
Catalytic action of tetrameric and monomeric human LDH‐A. (a) Dependence of the initial velocity of β‐NADH oxidation observed as a function of pyruvate concentration, in the presence of 125 μM β‐NADH and 2.6 nM tetrameric (white circles) or 9.8 nM monomeric (black circles) human LDH‐A. The continuous lines represent the best fit of the Michaelis–Menten equation to the experimental observations. (b) Dependence of the initial velocity of β‐NADH oxidation observed as a function of β‐NADH concentration, in the presence of 500 μM pyruvate and 1.9 nM tetrameric (white circles) or 7.6 nM monomeric (black circles) human LDH‐A. The continuous lines represent the best fit of the Michaelis–Menten equation to the experimental observations. (c) Kinetic parameters determined by performing activity assays using monomeric or tetrameric LDH‐A, at the indicated final concentrations. Under conditions of variable pyruvate concentration, β‐NADH was always used at 125 μM. When reaction mixtures contained variable concentrations of the redox cofactor, pyruvate was invariably added at 500 μM. All the assays were performed at pH 7.5 (Tris‐BisTris, 10 mM each).

To further characterize the catalytic action of monomeric LDH‐A exposed to β‐NADH and pyruvate, and therefore assembled into tetramer (cf. Figure 3c), we determined the enzyme activity as a function of pyruvate or β‐NADH concentration. In addition, the kinetics of pyruvate reduction catalyzed by tetrameric LDH‐A was assayed under the same conditions used for the monomeric enzyme. Structurally and functionally speaking, the availability of monomeric LDH‐A does indeed provide an unprecedented and quite interesting tool to perform quantitative tests of the assembly of monomers into the corresponding catalytically active tetramer. When reaction velocity was observed as a function of pyruvate concentration in the presence of 125 μM β‐NADH, tetrameric LDH‐A outperformed its monomeric counterpart, featuring a lower *K*_m_ and a higher *k*_cat_, that is, 271 ± 40 versus 561 ± 74 μM and 66 ± 5 versus 32 ± 3 s^−1^ (Figure 6a,c). In addition to this, the enzyme kinetic parameters were also determined testing reaction velocity as a function of β‐NADH concentration, in the presence of 500 μM pyruvate. Under these conditions, tetrameric LDH‐A was again observed to outperform the assembled monomers, essentially featuring the same *K*_m_ for the redox cofactor (9.0 ± 0.6 and 7.7 ± 1.1 μM, for tetramer and assembled monomers, respectively) and a three‐fold higher *k*_cat_, that is, 90 ± 2 and 29 ± 1 s^−1^, respectively (Figure 6b,c).

The divergence between the kinetic parameters determined for monomeric and tetrameric LDH‐A should be interpreted in terms of the monomers‐to‐tetramer assembly. In particular, when the *k*_cat_ values are considered, the ratio of their values suggests that approximately one half to one third of monomers generates tetramer, in the presence of excess pyruvate and β‐NADH, respectively. Moreover, it is interesting to note that the *K*_m_ of assembled monomers for pyruvate is about twice the value determined for tetrameric LDH‐A. This observation suggests that pyruvate is capable to stabilize tetrameric LDH‐A by binding to a secondary site, featuring low affinity for the monocarboxylic acid.

### 2.6 Design of peptides directed against LDH‐A subunit–subunit interactions

**FIGURE 7:**
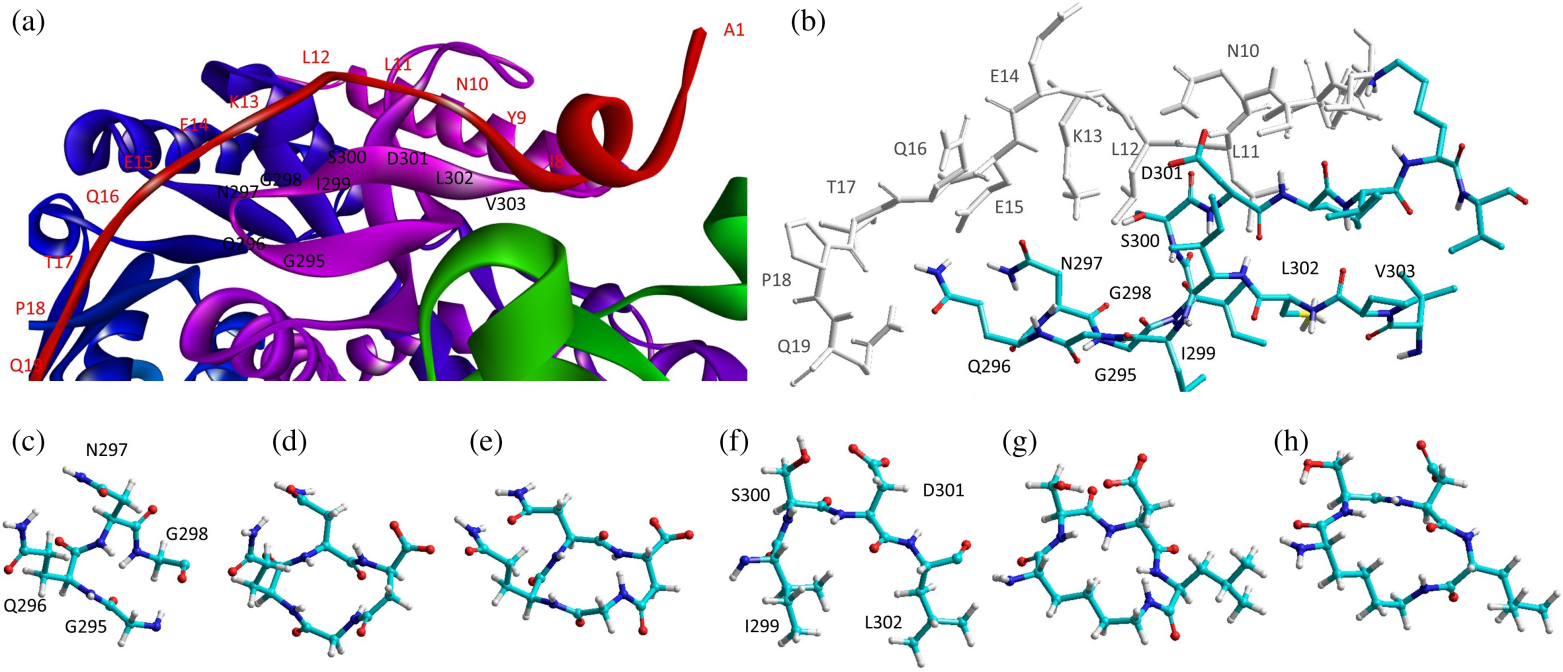
Design of the peptides to be tested against monomeric LDH‐A. (a) Details of the interactions between the N‐terminal region of one monomer with the residues 295–302 located at the C‐terminus of a second monomer, and (b) stick representation of the same region. Structural details of: (c) the G^295^QNG^298^ turn, and (d) the 13‐membered cyclopeptides c[GQN‐isoD] (TH2) and (e) c[GQN‐(*R*)‐isoD] (TH3), (f) the I^299^SDL^302^ turn, and the cyclopeptides (g) c[isoKSDL] (TH4) and (h) c[isoKSD‐(*R*)‐L] (TH5).

The inspection of two quaternary structures available for LDH‐5 (PDB files 1I10 and 4OJN: Read et al., 2001; Kolappan et al., 2015) led us to identify the N‐terminal region of LDH‐A as the target for inhibiting the assembly of the enzyme tetrameric form. In particular, since LDH‐5 can be considered as a binary association of dimers, we focused on the interactions between the residues 10–20 of the N‐terminal region of one monomer, and the residues 296–303 near the C‐terminus of the second monomer (Figure 7a). Accordingly, we designed the linear octapeptide TH1 as a potential inhibitor of LDH‐A oligomerization, featuring the primary structure GQNGISDL, the sequence of which is identical to the stretch of residues 296–303 of the target protein. To correlate the coordinates of peptides with those of LDH‐5 as denoted in the PDB files (shifted to a − 1 position due to the lack of the methionine coded by the start codon) the positions of the amino acids in the peptides are hereafter accordingly indicated (e.g., the residues 296–303 translate into 295–302). Interestingly, it can be recognized that the stretch of amino acids 295–302 does clearly feature two turns, centered on the tetrapeptides G^295^QNG^298^, and I^299^SDL^302^, respectively (Figure 7b). To design cyclic peptides inspired by these turns, we performed molecular modeling simulations. Plausible 3D structures of candidate turn mimetics were estimated by simulated annealing and molecular dynamics (MD) simulations, using the AMBER force field in explicit water (Cornell et al., 1995). In brief, random geometries of each cyclopeptide were sampled during a high temperature unrestrained MD simulation in a box of TIP3P models of equilibrated water molecules (Jorgensen et al., 1983). Each random structure was slowly cooled, the resulting structures were minimized, and the backbones of the structures were clustered by rmsd analysis. Only candidates showing one major cluster comprising the large majority of the geometries were considered. From this cluster the representative structures with the lowest energy were then selected and their conformations compared to those of the G^295^QNG^298^ (Figure 7c) and I^299^SDL^302^ (Figure 7f) turns. This procedure resulted in the in silico selection of two mimetics of G^295^QNG^298^ (Figure 7c), that is, the cyclopeptides c[GQN‐isoD] (TH2, Figure 7d) and [GQN‐(*R*)‐isoD] (TH3, Figure 7e), in which either (*S*)‐ or (*R*)‐Asp is framed within a 13‐membered ring as a β‐amino acid (isoAsp). The structures calculated for the 13‐membered cyclotetrapeptides are not unexpected. Indeed, β‐amino acids are well known to stabilize defined secondary structures, in particular γ‐turns or pseudo‐γ‐turns (Glenn et al., 2003).

In addition, by means of this strategy two mimetics of I^299^SDL^302^ were selected (Figure 7f), namely the cyclopeptides c[isoKSDL] (TH4, Figure 7g) and c[isoKSD‐(*R*)‐L] (TH5, Figure 7h), in which (*S*)‐ or (*R*)‐Lys is involved in the macrolactamization by the side‐chain amino group (isoLys). The chemical sketches of the cyclopeptides and of the majority of the intermediates are reported in Table 1. These cyclopeptides were prepared by cyclization of linear precursors, obtained in turn by solid phase peptide synthesis. To this aim, the tetrapeptide GQNG was modified into the partially protected GQND‐OBn (TH6) or GQN‐(*R*)‐d‐OBn (TH7), while ISDL was modified into Fmoc‐KSD(OBn)L (TH8) or Fmoc‐KSD(OBn)‐(*R*)‐L (TH9). These linear sequences were synthesized using standard procedures on a Wang resin using Fmoc‐protected amino acids, and DCC/HOBT as activating agents (Table S1). (*S*)‐ or (*R*)‐Asp was introduced as Fmoc‐Asp(OBn)OH, or Fmoc‐Asp(OBn); Lys was introduced as Fmoc‐Lys(Boc)OH. Fmoc deprotection was carried out using 20% (v/v) piperidine in DMF. Cleavage from the resin and simultaneous removal of the acid‐labile protecting groups was performed by using TFA in the presence of scavengers.

**TABLE 1:** Structural and functional features of the linear and cyclic peptides assayed as candidate inhibitors of LDH‐A activity.

Peptide	Structure	Sequence	Inhibition (%)
TH1	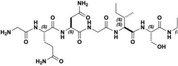	GQNGISDL	36 ± 1
TH2	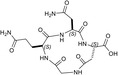	c[GQN‐isoD]	31 ± 3
TH3	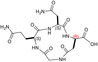	c[GQN‐iso(*R*)D]	26 ± 1
TH4	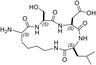	c[isoKSDL]	16 ± 3
TH5	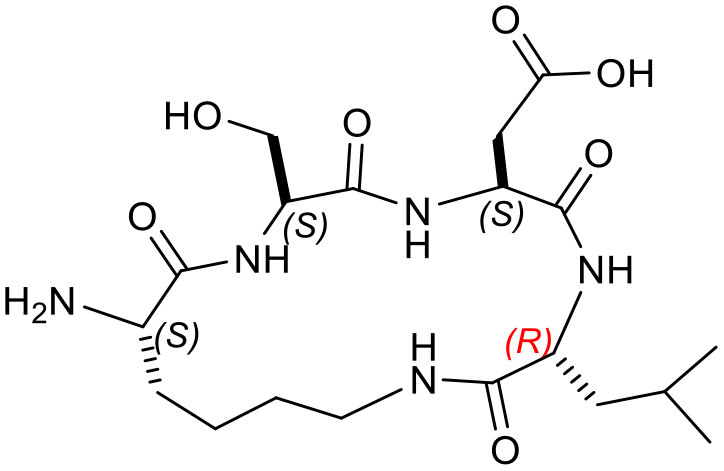	c[isoKSDL]	7 ± 3
TH6	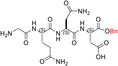	GQND‐OBn	14 ± 7
TH7	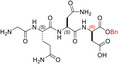	GQN‐(*R*)D‐OBn	4 ± 2
TH10	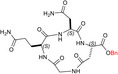	c[GQN‐isoD(OBn)]	6 ± 3
TH11	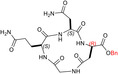	c[GQN‐(*R*)isoD‐(OBn)]	8 ± 4
TH14	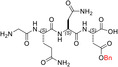	GQND(OBn)	15 ± 4
TH15	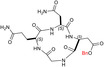	c[GQND(OBn)]	9 ± 2
TH16	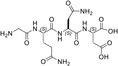	GQND	22 ± 2
TH17	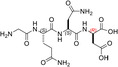	GQN‐(*R*)D	4 ± 8
TH18	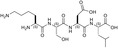	KSDL	5 ± 6
TH19	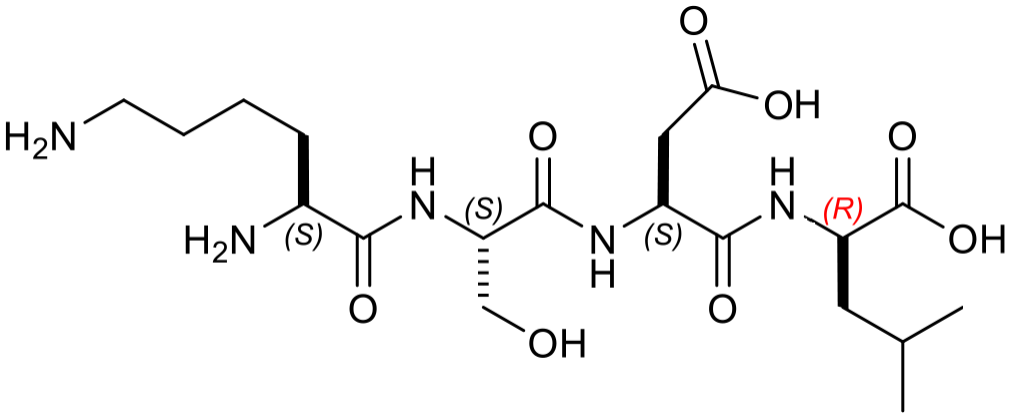	KSD‐(*R*)L	4 ± 3
DS105	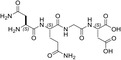	NQGD	0

*Note*: The SD (*n* = 3) associated with each estimated value of inhibition is indicated.

The cyclization of the partially protected linear peptides was performed under pseudo‐high dilution conditions, by slowly adding the peptide to a mixture of NaHCO_3_ and DPPA in DMF. The crude cyclopeptides c[GQN‐isoD(OBn)](TH10), c[GQN‐(*R*)‐isoD(OBn)] (TH11), c[isoK(Fmoc)‐SD(OBn)L] (TH12), c[isoK(Fmoc)‐SD(OBn)‐(*R*)‐L] (TH13), were isolated by RP HPLC on a semipreparative C18 column (Table S1). Removal of benzyl protecting groups was performed in quantitative yield by catalytic hydrogenation, giving c[GQN‐isoD] (TH2), c[GQN‐(*R*)‐isoD] (TH3), while Fmoc deprotection was done as described above, affording c[isoKSDL] (TH4), c[isoKSD‐(*R*)‐L] (TH5). The purities of the final products were determined to be >95% by RP HPLC (Table S1), and their identity was confirmed by ESI‐MS, ^1^H and ^13^C NMR, and by 2D gCOSY experiments at 600 MHz in DMSO‐*d*_*6*_.

We also prepared and tested the partially protected linear peptides GQND(OBn) (TH14), whose cyclization provided the 12‐membered c[GQND(OBn)] (TH15). Furthermore, starting from the partially protected linear peptides described above, we prepared and assayed the fully deprotected linear sequences GQND (TH16), GQN‐(*R*)‐D (TH17), KSDL (TH18), and KSD‐(*R*)‐L (TH19) (Tables 1, and S2).

### 2.7 Peptides and inhibition of LDH‐A activity

**FIGURE 8:**
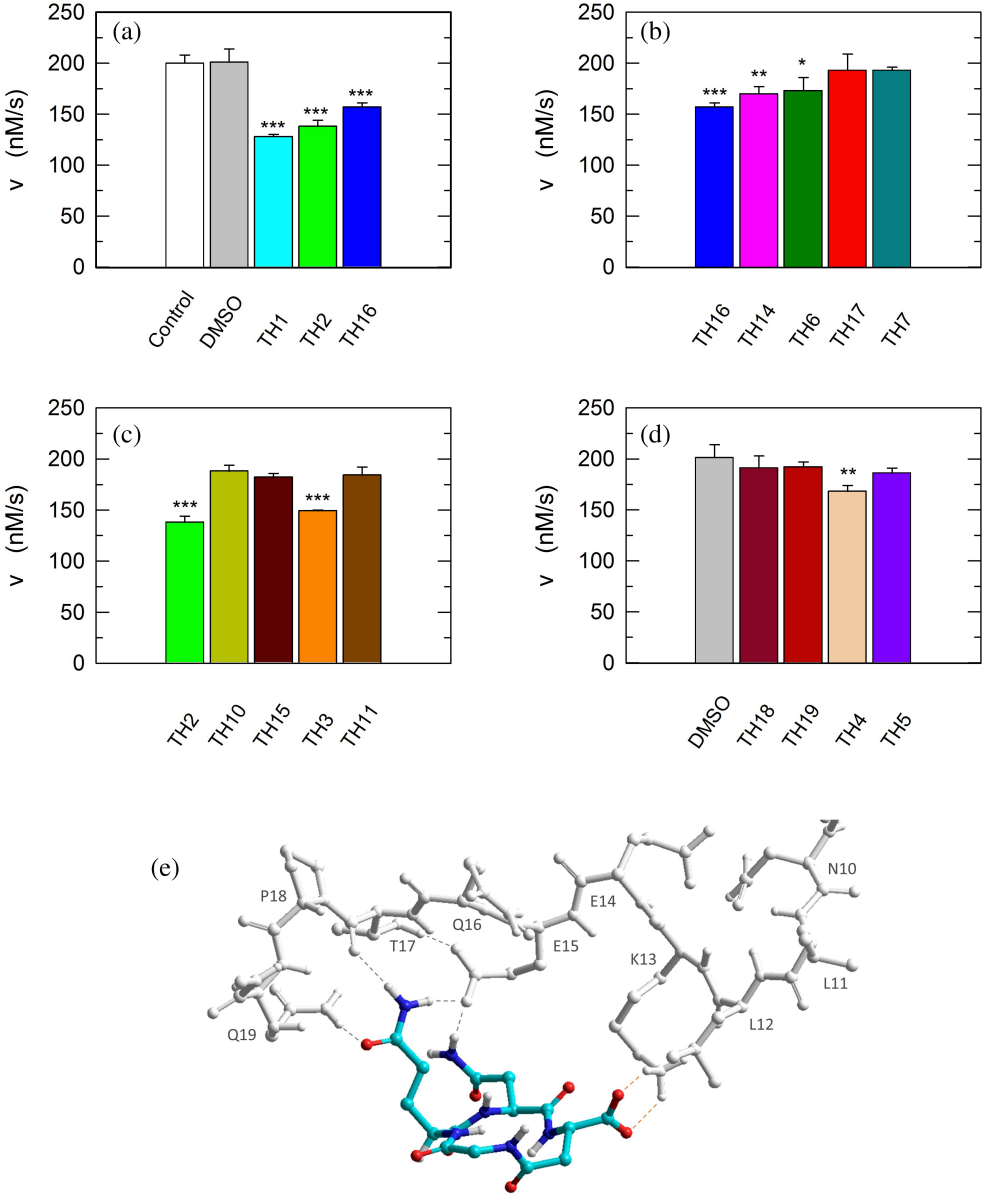
Inhibition exerted by linear and cyclic peptides on LDH‐A activity detected *in vitro*. Activity assays were performed with reaction mixtures containing 6.2 nM monomeric LDH‐A, 125 μM β‐NADH, and 500 μM pyruvate in Tris‐BisTris (10 mM each) buffer, pH 7.5 (white bar in “a”). The gray bars (in “a” and “d”) represent the activity observed in the presence of the same volume of DMSO (1%, v/v) carried to the assay mixtures by the peptides to be tested. (a–c) The effect, if any, of tetrameric peptides featuring the GQND primary structure (see also Table 1) on LDH‐A activity is shown. The inhibition exerted by the octameric peptide TH1 is also reported in “a.” The enzyme activity observed in the presence of linear or cyclic peptides is shown in “b” and “c”, respectively. (d) Extent of LDH‐A activity detected in the presence of linear or cyclic tetrapeptides featuring the KSDL primary structure. Error bars represent SD (*n* = 3). The experimental observations were compared by Student's *t*‐test. The ***, **, and * symbols denote *p* values lower than 0.001, 0.01, and 0.02, respectively. (e) Simulation of the association between the TH2 peptide and monomeric LDH‐A. Predicted interactions between the cyclopeptide c[GQN‐isoD] (TH2) and the N‐terminal sequence Y^9^NLLKEEQTPQ^19^, as simulated by molecular dynamics in a box of explicit TIP3P water molecules; gray and red dotted lines represent hydrogen bonds and salt bridges, respectively.

As previously mentioned, monomeric LDH‐A assembles into the corresponding tetramer when exposed to β‐NADH and pyruvate (or β‐NADH and oxamate, see Figure 1c), therefore gaining dehydrogenase activity. Accordingly, to test the action of peptides against the assembly of tetrameric LDH‐A, we performed activity assays under steady‐state conditions, in the presence of monomeric enzyme, 125 μM β‐NADH and 0.5 mM pyruvate. In particular, all the peptides were tested at 80 μM (final concentration), and the LDH activity detected in their presence was compared with the catalytic action observed with control samples devoid of any peptide. First, we decided to assay the octameric peptide TH1, the primary structure of which (GQNGISDL) is identical to the LDH‐A region spanning residues 295–302. Remarkably, the presence of this octapeptide was found to decrease the observed LDH activity by 36% (Figure 8a and Table 1). Moreover, it is important to note that when the TH1 octapeptide was tested against tetrameric LDH‐A no significant inhibition of the enzyme activity was observed. Indeed, the catalytic action exerted by 6 nM tetrameric LDH‐A (in 10 mM Tris–HCl, pH 7.5, containing 150 mM NaCl) was determined as equal to 433 ± 11 and 452 ± 19 nM/s in the absence and in the presence of 80 μM TH1 octapeptide, respectively. Overall, this insensitivity along with the significant effect elicited by TH1 on monomeric LDH‐A indicate that the octapeptide inhibits the assembly of LDH‐A monomers into the corresponding, catalytically active, tetramer. To dissect the inhibition triggered by TH1 on monomeric LDH‐A, we decided to evaluate: (i) whether or not the two halves of the octapeptide, that is, GQNG and ISDL, are equally competent in inhibiting LDH‐A; (ii) the action of linear and cyclic peptides, containing or not D amino acids; and (iii) the effect, if any, triggered by the presence of protecting groups in the peptides to be assayed. The structures and the distinctive features of all peptides considered are reported in Table 1. It should be noted that to allow the cyclization of tetrapeptide GQNG while maintaining a terminal carboxylic group, the sequence was modified to GQND, while for the cyclization of ISDL while maintaining a terminal amino group, the sequence was modified into KSDL (Table 1).

Remarkably, the GQND linear tetrapeptide TH16 was found to be a quite effective inhibitor, being responsible for a 22% decrease of LDH‐A activity (Figure 8a and Table 1). In addition, to obtain a scrambled variant of TH16 presumably devoid of inhibitory action, we constructed DS105, a linear tetrapeptide featuring the NQGD primary structure. As expected, the catalytic action of LDH‐A is insensitive to the presence of DS105 in the assay mixture (data not shown). Furthermore, we did not detect a significant inhibition of LDH‐A in the presence of TH17, which differs from TH16 for the specific substitution of l‐aspartate with d‐aspartate (Table 1 and Figure 8b). Therefore, the replacement of a single l‐amino acid with its d‐counterpart does completely suppress the interference exerted by TH16 on the assembly of LDH‐A monomers into the corresponding catalytically active tetramer. The effect, if any, of the presence of a protecting benzyl group in the GQND tetrapeptide was then analyzed. In particular, the inhibitory action of different protected peptides was compared with the action of the TH16 peptide, and the following observations were accordingly obtained: (i) the TH14 peptide, bearing a benzyl group bound to the C′‐carboxyl (Table 1), performs less than its progenitor TH16 (Figure 8b); (ii) the TH6 peptide, featuring a benzyl group located at the Cβ‐carboxyl (Table 1), does weakly perform when compared to TH16, being responsible for 14% inhibition of LDH‐A activity (Figure 8b); (iii) the presence in TH7 of a benzyl group bound to the C′‐carboxyl (Table 1) does not confer a significant inhibitory action to this tetrapeptide, which therefore behaves as its inactive parental unprotected peptide TH17 (Figure 8b). Rather interestingly, when cyclic and unprotected GQND tetrapeptides were assayed, a similar extent of LDH‐A inhibition was observed with TH2 and TH3 (Table 1), containing l‐ and d‐aspartate, respectively (Figure 8c). In addition to this, we determined that cyclic GQND TH10, TH11, and TH15 tetrapeptides, bearing a benzyl protecting group, were outperformed by their unprotected counterparts (Figure 8c).

Overall, the KSDL tetrapeptides were found less effective than their GQND counterparts. In particular, the cyclic TH4 tetrapeptide was found to inhibit LDH‐A activity by 16% only (Figure 8d and Table 1), which is significantly lower than the level of inhibition exerted by TH2 (Figure 8c and Table 1).

### 2.8 Molecular modeling

According to our observations, among the cyclopeptides tested c[GQN‐isoD] (TH2) and [GQN‐(*R*)‐isoD] (TH3), feature the highest inhibitory efficiency (Figure 8c). Apparently, the cyclic structure contributes to improve their performances, possibly by imposing a proper 3D structure mimicking the G^295^QNG^298^ turn. Nevertheless, the presence of a free carboxylic group seems also quite important, as suggested by the very poor efficacy of the corresponding benzyl esters c[GQN‐isoD(OBn)] (TH10), and c[GQN‐(R)‐isoD(OBn)] (TH11). To ascertain any role played by the free carboxylic group in the inhibition of LDH‐A oligomerization, we predicted by molecular modeling and molecular dynamics the possible interactions between the cyclopeptide c[GQN‐isoD] (TH2) and the N‐terminal target sequence of the monomer (Figure 7a,b). The structures of the interacting N‐terminal residues 9 to 19 and the C‐terminal residues 295–302 were obtained from the quaternary structure reported by Read et al. (PDB file 1I10, Read et al., 2001). To prime the analysis, the cyclopeptide TH2 was superimposed to the G^295^QNG^298^ turn, then the C‐terminal sequence was removed. The system accordingly obtained was subjected to molecular dynamics simulations at 298 K in a box of explicit TIP3P equilibrated water molecules. During this period, the geometry of the N‐terminal 9–19 sequence was restrained by applying a force field to dihedral angles and distances, while the cyclopeptide was maintained unrestrained. This step was followed by a stage with a scaled force field, and finally by a phase of unrestrained dynamics. During the last step, the conformation of the N‐terminal sequence 9–19 slightly changed, allowing the formation of a salt bridge between the carboxylate of cyclopeptide TH2 and the protonated amino side chain of Lys^13^ of the target N‐terminal region (Figure 8e).

With all due caution, the simulations seem to point at this interaction as a main contributor of the comparatively higher inhibitory efficacy of the cyclopeptides.

### 2.9 Inhibitory action of peptides on lactate production by human cells

**FIGURE 9:**
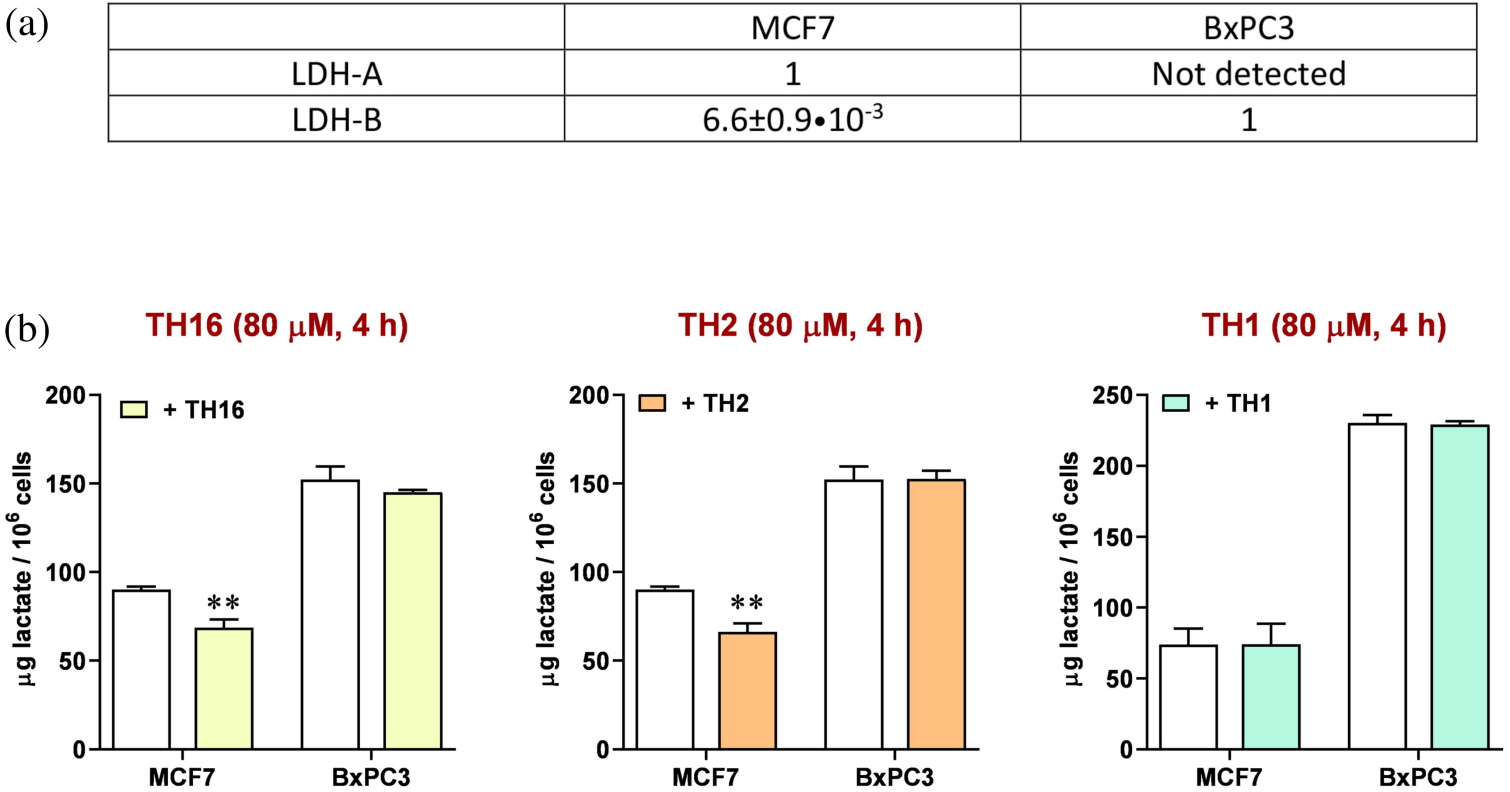
Effect of peptides on the secretion of lactate by human cell lines. (a) Relative levels of mRNA coding for LDH‐A or LDH‐B as detected by RT‐PCR in MCF7 cells. Only the mRNA coding for LDH‐B was detected in BxPC3 cells. (b) The amount of lactate secreted by MCF7 or BxPC3 cells in the absence (white bars) or in the presence (yellow, orange, and green bars) of different peptides is shown. Error bars represent SD (*n* = 3). The experimental observations were compared by two‐way ANOVA. The ** symbol denote a *p* value lower than 0.01.

Having identified peptides able to interfere with the assembly of monomeric LDH‐A into catalytically active tetramer, we considered of interest to test the action of peptides against LDH‐A expressed in human cell lines. In particular, we analyzed the lactate secreted by cultured human cells exposed or not to the peptides better performing as inhibitors of LDH‐A *in vitro*, that is, TH1, TH2, and TH16. Moreover, we used two different cell lines exclusively expressing LDH‐A or LDH‐B, that is, MCF7 and BxPC3, respectively. In MCF7 cells, the [mRNA_LDH‐A_]/[mRNA_LDH‐B_] ratio is indeed equal to ca. 150 (Figure 9a), whereas in BxPC3 cells the messenger coding for LDH‐A was not detected (Figure 9a). Therefore, by comparing the decrease, if any, of the lactate concentration secreted by these two cell lines the selectivity of peptides acting against LDH‐A or LDH‐B can be conveniently estimated. When the MCF7 cell line is considered, the linear TH16 and the cyclic TH2 tetrapeptide performed equally, being both responsible for a concentration of secreted lactate 20% lower than that detected in their absence (Figure 9b). On the contrary, and rather interestingly, when BxPC3 cells were used no significant differences were observed between the lactate secreted by cells exposed or not to the tetrapeptides (Figure 9b). Surprisingly enough, the TH1 octapeptide did not induce any significant effect on lactate secretion by MCF7 and BxPC3 cells (Figure 9b). We propose that the absence of a detectable phenotype linked to the treatment of cells with TH1 is due to a poor import of this octameric peptide, which proved to be rather effective when tested in vitro. It should indeed be noted that the assays with cells were performed in the presence of lipofectamine (see Section 5). In addition, it is important to mention that when tested in the absence of lipofectamine both the TH16 and the TH2 tetrapeptide were found to trigger negligible and not significant effects on lactate production by MCF7 cells. Accordingly, it seems likely that lipofectamine is effective in assisting the penetration of the TH16 and TH2 peptides into cells, with this action of lipofectamine being much weaker when dealing with a larger molecule, that is, an octapeptide.

## 3 DISCUSSION

So far, the functional and structural features of human LDH‐A were almost exclusively inspected using LDH‐5, that is, the tetrameric form of this enzyme. Accordingly, we considered of interest to isolate homogeneous monomeric LDH‐A, the availability of which could be of help to investigate the assembly of tetrameric LDH‐A. In addition to this, it should be mentioned that the procedure reported here for the purification of monomeric human LDH‐A can be conveniently modified to isolate the corresponding tetramer. In particular, the addition of β‐NADH and oxamate or 150 mM NaCl to enzyme samples being subjected to the last purification step, that is, gel filtration chromatography, translates into the isolation of LDH‐5 (Figure 1c,d). Remarkably, these observations are in substantial agreement with those reported by Trausch for rabbit skeletal muscle lactate dehydrogenase (Trausch, 1972). Tetrameric rabbit LDH‐A was indeed found to dissociate into dimers when the enzyme was loaded onto a Sephadex G‐200 column under conditions of high ionic strength (1 M NaCl), unless 100 μM β‐NADH and 10 mM pyruvate were added to the buffer used for the gel filtration chromatography. Therefore, the observations shown here and those obtained by Trausch indicate that both the redox cofactor and the monocarboxylic substrate are important factors promoting the stability of tetrameric LDH‐A. Quite intriguingly, by means of gel filtration chromatography we determined the molecular mass of monomeric human LDH‐A as equal to 19.8 kDa (Figure 1), a value considerably lower than expected. This peculiar observation could represent the output of a particularly slow diffusion of human monomeric LDH‐A along the void volume of the chromatographic column, implying an increase of the enzyme elution volume compared to the expected value, which is related to the diffusion of the enzyme into the chromatographic beads. This, in turn, implies that the conformation of monomeric human LDH‐A is rather extended, conferring to the enzyme a molecular volume larger than that of a single subunit of tetrameric LDH‐A. This feature of the enzyme could be responsible for the unexpected observations obtained when monomeric LDH‐A was analyzed by DLS. Indeed, the diameters of monomeric LDH‐A molecules would be, according to the DLS experiments, larger than those determined for the same protein in the presence of β‐NADH or in the presence of both β‐NADH and oxamate (Figure 3). It is important to remind that this last condition translates into the assembly of monomers into tetramers, the molecular mass of which was estimated by DLS in excellent agreement with the expected value.

When the competence of monomeric LDH‐A in binding β‐NADH was tested by surface plasmon resonance (SPR) a weak interaction was detected, the occurrence of which did not translate into an appreciable saturation of the binding site (Figure 4). This suggests that the binding of β‐NADH is severely inhibited when the mutual association of LDH‐A monomers is hampered in the immobilized state. Interestingly, when dissociated rabbit LDH‐A was immobilized and exposed to free enzyme subunits the assembly into the tetrameric form occurred, albeit this competence was rapidly lost (Chan & Mosbach, 1976). Conversely, when free monomeric LDH‐A is hosted in solutions containing β‐NADH and pyruvate the assembly into the tetrameric forms is triggered, conferring competence in catalytic action. Moreover, the extent of this assembly should be mirrored by the level of catalytic activity, which can be compared with the action exerted by the tetrameric enzyme at an equal concentration of subunits. Accordingly, activity assays were performed as a function of monomeric or tetrameric LDH‐A concentration, in the presence of 100 μM β‐NADH and 500 μM pyruvate. Remarkably, the observed kinetics of β‐NADH oxidation suggest that 20–40% of LDH‐A monomers do assemble into tetrameric enzyme (Figure 5b–d). In addition to these tests, we also determined the kinetic parameters of tetrameric and monomeric LDH‐A. Concerning the tetrameric enzyme, *k*_cat_ values equal to 65.5 and 89.9 s^−1^ were determined under conditions of variable pyruvate or β‐NADH concentration, respectively (Figure 6c). The corresponding values for monomeric LDH‐A were estimated as equal to 32.4 and 29 s^−1^ (Figure 6c), suggesting that 30%–50% of monomers do assemble into the tetrameric form of the enzyme. It should be noted that these values are slightly higher than those (20%–40%) determined detecting the oxidation of β‐NADH under conditions exclusively yielding first‐order kinetics and implying the depletion of a considerable quantity of the redox cofactor (which with pyruvate is responsible for the assembly of tetrameric LDH‐A, Figure 5b,c).

When the *K*_m_ for pyruvate is considered, we determined a value equal to 560 and 271 μM for monomeric and tetrameric LDH‐A, respectively (Figure 6c). It should be mentioned that the *K*_m_ value of the tetrameric enzyme is in reasonable agreement with previous observations, yielding values equal to 252 and 398 μM (Pasti et al., 2022; Pettit et al., 1981). Accordingly, monomeric LDH‐A features a rather high *K*_m_ for pyruvate and, as previously mentioned, we interpret this feature as indicative of a secondary site for pyruvate, possibly engaged in the stabilization of the assembled tetramer.

Structurally speaking, the four subunits of LDH‐5 are arranged as a binary association of dimers, with their N‐ and C‐terminal regions reciprocally engaged in dimer‐dimer interactions (Read et al., 2001). As a target of peptides hopefully able to interfere with the assembly of LDH‐5, we selected the N‐terminal region of LDH‐A. We indeed reasoned that any peptide identified as competent in inhibiting the assembly of monomeric LDH‐A into tetrameric LDH‐5 *in vitro*, could also be able to interact with the N‐terminal part of the nascent LDH‐A displayed by ribosomes during mRNA translation. Moreover, it should be considered that it was previously shown that the N‐terminal region of LDH‐A represents a better target than its C‐terminal part (Nadal‐Bufi et al., 2021). Accordingly, we designed the TH1 octapeptide, featuring the primary structure GQNGISDL, which is identical to the C‐terminal part of LDH‐A consisting of residues 295–302. Remarkably, the effectiveness of this octapeptide against the assembly of LDH‐5 is rather satisfactory, its presence in assay mixtures being responsible for a 36% decrease of the observed LDH‐5 activity. This observation diverges from the output reported for a quite similar peptide tested against LDH‐A, that is, the nonamer Ac‐QNGISDLVK‐NH_2_ (denoted C1), which resulted ineffective (Nadal‐Bufi et al., 2021). The reason for this apparent discrepancy should reside in the different procedures used to evaluate the action of the TH1 and C1 peptides. Indeed, Nadal‐Bufi et al. assayed the C1 nonapeptide using LDH‐5 exposed to pH 2.5 to dissociate its subunits, incubating the dissociated enzyme to the peptide for 2 min, and finally shifting the pH to 7.4. On the contrary, as reported here, we used monomeric LDH‐A at pH 7.5 as the target of peptides selected as candidates to interfere with the assembly of LDH‐5. Therefore, it seems quite likely that exposing both the target and the candidate peptide to pH 2.5 could limit their mutual interactions.

As previously mentioned, we dissected the action of TH1 using two different families of tetrapeptides, and we attempted to identify the molecular determinants conferring to the better performers the competence in inhibiting the assembly of LDH‐5. Accordingly, we recognized the importance of constructing unprotected peptides, and featuring a cyclic structure. In particular, we observed that the cyclic TH2 tetrapeptide outperformed its conjugate linear TH16 peptide, with both of them featuring the GQND primary structure. We propose that this difference is likely due to the conformational stability conferred by cyclization to TH2, the binding competence of which to monomeric LDH‐A would nevertheless be supported by the stretchy conformation of the target. According to the tertiary structure of LDH‐A, the N‐terminal region spanning amino acids 12–21 is indeed arranged as a coil, therefore featuring conformational freedom in the monomeric state. Nevertheless, the cyclic nature of TH2 implies that a limited number of amino acids, residing on one side of this tetrapeptide, would be able to interact with the target. In particular, we propose that the side containing the couple of residues QN could favorably interact with residues located in the region of the target spanning amino acids 14–19, as previously reported (Jafary et al., 2019). In addition to the difference between the action of peptides TH2 and TH16, it is important to note further unprecedented observations that we have shown here: (i) the inhibition exerted on LDH‐A by linear peptides is sensitive to their stereochemistry, as indicated by the finding that replacing the l‐aspartate in the effective TH16 peptide with a d‐aspartate yields the ineffective conjugate TH17 peptide (Table 1); (ii) the inhibition of LDH‐A triggered by cyclic peptides is insensitive to the presence of l‐ or d‐aspartate, as determined for the TH2 and TH3 peptides (Table 1). Remarkably, these observations suggest the use of conformationally restricted cyclic peptides, preferably containing d‐amino acids to limit their degradation *in vivo*.

We reported here for the first time on the inhibition of LDH‐A exerted in cultured cells by tetrapeptides. Remarkably, by means of assays detecting the lactate secreted by cell lines exclusively expressing LDH‐A or LDH‐B we were able to show that the tetrapeptides TH16 and TH2 feature high selectivity against LDH‐A. However, the octapeptide TH1 that was found to be the best performer *in vitro* did not elicit any effect in cultured cells, probably because of a limited import of this peptide in the cellular cytosol. Accordingly, it will be of interest to construct chimeric peptides, containing a region targeting LDH‐A and a arginine‐ and/or lysine‐rich part, the presence of which could mimic cell‐penetrating peptides (Gori et al., 2023; Green & Loewenstein, 1988; Joliot et al., 1991). The heptadecameric cGmC9 peptide constructed and tested by Nadal‐Bufi et al. was indeed designed to contain four arginines (Nadal‐Bufi et al., 2021), most likely favoring its import into cells' cytosol, the compartment where LDH‐A is located and can therefore be targeted. Moreover, and importantly, it was shown that this type of peptides can be successfully used to inhibit the proliferation of cancer cells (Nadal‐Bufi et al., 2022).

## 4 CONCLUSIONS

We have reported here on the isolation of homogeneous human LDH‐A in monomeric form by means of a simple purification procedure performed at pH 7.5. Remarkably, this finding provides a convenient and reliable tool to assay candidate inhibitors directed against the assembly of monomeric hLDH‐A into the corresponding, catalytically active, tetramer. Moreover, we have shown that appropriately designed cyclic peptides can exert this inhibitory action. Considering the relevance of LDH‐A in the energetic metabolism of malignant cells, it is our hope that the availability of monomeric hLDH‐A will be of help to the identification of further peptides repressing glycolysis in cancer cells by means of a strong inhibition of the protein–protein interactions essential to the assembly of tetrameric hLDH‐A.

## 5 MATERIALS AND METHODS

### 5.1 Purification of LDH‐A monomer

To obtain human LDH‐A in monomeric form, a gene coding for this enzyme and optimized for *Escherichia coli* codon usage was synthesized (GenScript, Leiden, NL) and cloned into the pET9a expression vector using the *Nde*I and *Bam*HI sites (Figure S1). The construct accordingly obtained was used to transform *E. coli* BL21(DE3) electrocompetent cells. Transformants were selected using Petri dishes containing LB solid medium supplemented with kanamycin (40 μg/ml), and subsequently purified on LB‐kan plates. Single colonies were then picked up to inoculate LB‐kan liquid medium, and grown for 15 h at 37°C under shaking conditions (180 rpm). The pre‐cultures accordingly obtained were diluted (1:500) into fresh LB‐kan medium and grown at 30°C for 9 h. Then, 1 mM isopropyl‐β‐d‐thiogalactopyranoside (IPTG) was added, and the induced cultures were incubated at 30°C for 15 h. Induced cells (300 ml) were harvested by centrifugation (4500×*g*, 20 min, 4°C), and the pellets were resuspended in 15 ml of 50 mM Na_2_HPO_4_, 150 mM NaCl, 1 mM EDTA, pH 7.5 (buffer A). Total proteins were extracted by sonication (Misonix‐3000 sonifier, output level of 18 W for 15 s, followed by a 15 s cooling interval, for 4 cycles). The crude protein extract accordingly obtained was centrifuged (13,000×*g*, 30 min, 4°C), the pellet was discarded, and the supernatant was immediately loaded onto a HiTrap Blue column (5 ml, GE Healthcare, Piscataway), previously equilibrated with buffer A. After extensive washing of the column with buffer A, LDH‐A was eluted with the same buffer supplemented with 0.5 M NaCl. The best fractions (as determined by activity assays and SDS‐PAGE analysis) were pooled, and solid NaCl was added to increase its concentration to 3 M in the pooled fractions. The sample accordingly obtained was loaded onto a HiTrap Phenyl FF column, previously equilibrated with Buffer A containing 3.0 M NaCl. After washing the column, a reverse NaCl gradient (3.0–0 M) was applied (10 column volumes), and afterwards LDH‐A was eluted with buffer A. The best eluted fractions were pooled, concentrated to 2 ml and loaded onto a Superdex‐200 column, equilibrated with 10 mM Tris–HCl, pH 7.5 (or alternatively 10 mM HEPES, pH 7.5). After pooling and concentrating the fractions containing homogeneous LDH‐A, aliquots of the concentrated purified enzyme were stored at −20°C until used. Protein concentration was determined according to Bradford (Bradford, 1976).

### 5.2 Activity assays

The enzyme‐catalyzed reduction of pyruvate was assayed by determining the decrease in Absorbance at 340 nm related to the oxidation of β‐NADH. The extinction coefficient of β‐NADH at 340 nm was considered equal to 6.22•10^3^ M^−1^ cm^−1^ (Bernofsky & Wanda, 1982). All the assays were performed at 25°C using a Cary 300 Bio spectrophotometer. To determine the *K*_m_ of monomeric or tetrameric LDH‐A for pyruvate, reaction mixtures contained 125 μM β‐NADH in 50 mM Tris–HCl (pH 7.5). Conversely, to determine the *K*_m_ of monomeric or tetrameric LDH‐A for β‐NADH reaction mixtures contained 500 μM pyruvate in 50 mM Tris–HCl, pH 7.5.

The inhibition exerted by peptides on LDH‐A activity was tested by incubating 6.2 nM enzyme and 80 μM peptide in 10 mM Tris–HCl, 10 mM BisTris (pH 7.5) buffer for 5 min, at the end of which the reaction was started by the addition of 125 μM β‐NADH and 500 μM pyruvate.

### 5.3 Dynamic light scattering

Dynamic light scattering experiments were performed with a Malvern Panalytical (Malvern, UK) Zetasizer Nano ZS system. All the measurements were recorded at 25°C using solutions previously filtered with 0.2 μm filters, and containing 5 μM enzyme in 10 mM Tris–HCl, pH 7.5. Scattering was evaluated at an angle of 173 degrees. Each individual observed value of enzyme diameter represents the average output of three groups of consecutive determinations. Raw data were analyzed with the Zetasizer software (Malvern Panalyticals), release 7.11.

### 5.4 Circular dichroism

CD spectra were recorded over the 200–250 nm wavelength interval at a scan rate equal to 50 nm/min, using a Jasco J‐810 spectropolarimeter and a 0.5 cm path‐length cuvette. Protein samples were in PBS, and the band width was set to 1 nm. Sixteen scans per sample were acquired and averaged.

### 5.5 Surface plasmon resonance

The binding of β‐NADH by monomeric and tetrameric hLDH‐A was assayed using a Biacore T200 instrument (Cytiva, Marlborough, MA). Both monomeric and tetrameric LDH‐A were immobilized on a Xantec CMD500 chip at a concentration of 30 μg/ml in 10 mM HEPES, pH 7 using a standard amine coupling protocol, yielding an observed immobilization level of 6984 and 17,424 response units (Rus), respectively. The binding of β‐NADH to hLDH‐A was tested in 50 mM HEPES, pH 7.5, at 25°C, using a flow rate of 50 μl/min, with 60 and 10 s of association and dissociation time, respectively. Different ranges of β‐NADH concentration were used, as indicated in the figures. The elaboration of sensorgrams was achieved using the BIAevaluation software.

### 5.6 Mass spectrometry

To verify the identity of monomeric LDH‐A, spots were excised from SDS‐PAGE gels and underwent trypsin‐in‐gel digestion as previously reported (Schevchenko et al., 2007). The resulting peptides were analyzed by LC–MS/MS using a Q‐Exactive instrument (Thermo‐Fisher Scientific, Waltham, MA) equipped with a nano‐ESI source coupled with an Ultimate capillary UHPLC as reported elsewhere (Conte et al., 2012).

### 5.7 Cell cultures and determination of lactate dehydrogenase expression

To evaluate the competence of peptides in inhibiting the action of LDH‐A in cultured cells, two human cell cultures were used: MCF7 and BxPC3, isolated from breast and pancreatic adenocarcinoma, respectively. Cultures were grown in DMEM (MCF7) or RPMI medium (BxPC3), supplemented with 100 U/ml penicillin/streptomycin, 2 mM glutamine and 10% (v/v) FBS. All the components of the media used for cell culture were obtained from Merck‐Millipore.

The expression of LDH subunits in both cell lines was evaluated by RT‐PCR. RNA extraction, retro‐transcription and DNA amplification were performed as previously described (Rossi et al., 2023). For each culture, the relative expression *ldh‐a/ldh‐b* was evaluated. The primers used for the RT‐PCR reaction were: (a) for LDH‐A: 5′‐GCAACCCTGCAACGATTTT‐3′ (forward), and 5′‐TTCCAGAGGACAAGATCTCAAA‐3′ (reverse); (b) for LDH‐B: 5′‐CCAACCCAGTGGACATTCTT‐3′ (forward), and 5′‐AAACACCTGCCACATTCACA‐3′ (reverse).

### 5.8 Colorimetric assays of lactate secretion by cultured human cells

The competence of peptides in inhibiting LDH in cultured cells was tested by assessing the concentration of lactate released by MCF7 and BxPC3 cell cultures. To this aim, cells were seeded in duplicate in 24‐wells plates (2 × 10^5^/well) and let to adhere overnight; the culture medium was then replaced by Krebs‐Ringer buffer (300 μl/well). Stock solutions of peptides (8 mM in DMSO) were diluted in Krebs‐Ringer buffer to a final concentration of 640 μM and mixed with an equal volume of 4% (v/v, in Krebs‐Ringer buffer) Lipofectamine 2000 (Invitrogen). The final solution was thoroughly mixed, kept for 25 min at room temperature, and finally 100 μl were added to each plate well, obtaining a final peptide concentration equal to 80 μM. The concentration of lactate released in the Krebs–Ringer buffer was determined after 4 h of incubation at 37°C, using the colorimetric assay previously reported (Rossi et al., 2023). Each experiment was performed in triplicate and included control samples consisting of untreated cell cultures exposed to the same final concentration of DMSO (1%, v/v) and Lipofectamine (0.5%, v/v) to which were exposed the samples treated with peptides.

### 5.9 Synthesis of peptides, general procedures

Unless otherwise stated, standard chemicals and solvents were purchased from commercial sources and used as received without further purification. The purity of target compounds was determined to be ≥95% by HPLC analyses, performed using an Agilent 1200 series apparatus, equipped with a Phenomenex reverse‐phase column (Gemini C18, 3 μm, 110 Å, 100 × 3.0 mm, no. 00D4439‐Y0). Column description: stationary phase octadecyl‐carbon chain‐bonded silica (C18) with trimethylsilyl end‐cap, fully porous organo‐silica solid support, particle size 3 μm, pore size 110 Å, length 100 mm, internal diameter 3 mm; mobile phase for neutral compounds: from 9:1 H_2_O/CH_3_CN to 2:8 H_2_O/CH_3_CN in 20 min at a flow rate of 1.0 ml min^−1^, followed by 10 min at the same composition; DAD (diode‐array detection) 210 nm; mobile phase for ionizable peptides: from 9:1 H_2_O/CH_3_CN and 0.1% HCOOH to 2:8 H_2_O/CH_3_CN and 0.1% HCOOH in 20 min, flow rate of 1.0 ml min^−1^; DAD 254 nm. Semipreparative RP HPLC was carried out with a Waters 2489 UV/visible Dual Detector equipped with a Waters 1525 Binary HPLC pump, using reverse‐phase column XSelect Peptide CSH C18 OBD column (Waters, 19 × 150 mm, 5 μm, no.186007021). Column description: stationary phase octadecyl‐carbon chain‐bonded silica (C18), double end‐capped, particle size 5 μm, pore size 130 Å, length 150 mm, internal diameter 19 mm; DAD 210 nm, DAD 254 nm. Mobile phase for neutral compounds: isocratic 6:4 H_2_O/CH_3_CN for 2 min, then gradient from 6:4 H_2_O/CH_3_CN to 2:8 H_2_O/CH_3_CN in 10 min, then isocratic 2:8 H_2_O/CH_3_CN for 2 min; flow rate: 10 ml min^−1^. Mobile phase for ionizable peptides: isocratic 6:4 H_2_O/CH_3_CN and 0.1% HCOOH for 2 min, then gradient from 6:4 H_2_O/CH_3_CN and 0.1% HCOOH to 2:8 H_2_O/CH_3_CN and 0.1% HCOOH in 10 min, then isocratic 2:8 H_2_O/CH_3_CN and 0.1% HCOOH for 2 min. Routine ESI MS analysis was carried out using an MS single quadrupole HP 1200 MSD detector, with a drying gas flow of 12.5 L min^−1^, nebulizer pressure 30 psig, drying gas temperature 350°C, capillary voltage 4500 (+) and 4000 (−), scan 50–2600 amu. NMR spectra were recorded with a Bruker BioSpin GmbH (^1^H: 600 MHz, ^13^C: 151 MHz) at 298 K in 5 mm tubes, using 0.01 M peptide. Chemical shifts are reported in ppm (δ) and internally referenced with (CD_3_)_2_SO: ^1^H: 2.50, ^13^C: 39.52 ppm. The unambiguous assignment of ^1^H NMR resonances was based on 2D gCOSY experiments. Coupling constants (J) are reported in Hz.

The NMR spectra and the HPLC chromatograms of the synthesized peptides are reported in Figures S5a–q and S6a–c, respectively.

### 5.10 Solid phase synthesis, general procedure for linear peptides

Solid‐phase peptide synthesis was performed in polypropylene syringes fitted with a polyethylene porous disc. The linear peptides were assembled manually on Wang resin (0.3 g, 1.1 mmol/g loading capacity) using Fmoc‐protected amino acids under standard procedures; (*S*)‐ or (*R*)‐Asp was introduced as Fmoc‐Asp(OBn)OH, or Fmoc‐Asp(OBn); Lys was introduced as Fmoc‐Lys(Boc)OH.

Prior to use, the resin was swollen in DMF (3 ml) for 15 min. In a separate sample tube, (*S*)‐ or (*R*)‐Fmoc‐AA‐OH (0.6 mmol) and HOBT (81 mg, 0.6 mmol) were dissolved in DMF (4 ml). After 20 min, the mixture was added to the resin, followed by DCC (124 mg, 0.6 mmol) and a catalytic amount of DMAP, and the resin was gently shaken at RT for 3 h. Thereafter, a mixture of Ac_2_O (0.92 ml, 10 mmol) and pyridine (0.81 ml, 10 mmol) was added and shaken for additional 30 min to end‐cap the unreacted 4‐hydroxybenzyl alcohol linkers. The resin was filtered and washed alternatively with DMF, MeOH, and DCM (3 × 4 ml each).

Fmoc cleavage was carried out using 20% (v/v) piperidine in DMF (5 ml), while gently shaking at RT for 20 min. After washing with DMF and DCM (5 ml), the deprotection was repeated. The resin was then washed sequentially with DMF, MeOH, and DCM (3 × 4 ml each).

The subsequent coupling reactions were performed by dissolving in a separate vial Fmoc‐protected amino acids (0.6 mmol) and HOBt (81 mg, 0.6 mmol) in DMF (4 ml) for 20 min. The mixture was poured into the reactor followed by DCC (124 mg, 0.6 mmol), and the suspension was shaken at RT for 3 h. Coupling efficacy was monitored by the Kaiser test. Fmoc cleavage was carried out as reported above.

Cleavage from the resin and simultaneous removal of the acid‐labile protecting groups was performed by using a 95:2.5:2.5 v/v/v mixture of TFA/TIPS/H_2_O (10 ml) while shaking for 2.5 h at RT. The mixture was filtered and the resin washed twice with 5% TFA in Et_2_O (10 ml). The filtrates were collected and solvents were removed under reduced pressure. Then ice‐cold Et_2_O was added to precipitate the crude peptides as TFA salts, which were recovered by centrifuge. The peptides were isolated by RP HPLC on a semipreparative C18 column. Linear sequences destined to cyclization were utilized without further purifications. The purity and the identity of the products were determined to be >95% by RP HPLC coupled to ESI MS, by ^1^H and ^13^C NMR.

### 5.11 Synthesis of cyclopeptides, general procedure

The cyclization of the crude peptides was performed under pseudo‐high dilution conditions, by slowly adding the peptide using a temporized syringe pump (dual‐channel KD Scientific model 200). A solution of the linear peptide (0.15 mmol) in DMF (10 ml) was added to a mixture of NaHCO_3_ (38 mg, 0.45 mmol) and DPPA (65 μl, 0.3 mmol) in DMF (4 ml) at RT over 16 h. Once the addition was complete, the reaction was stirred for additional 6 h. Then the solvent was distilled under reduced pressure, and the residue was suspended in water (5 ml), and extracted 3 times with EtOAc (20 ml). The combined organic solvent was removed at reduced pressure, and the crude peptides were isolated by RP HPLC on a semipreparative C18 column.

Removal of benzyl protecting groups was performed by catalytic hydrogenation. A stirred suspension of the protected peptides (0.1 mmol) and a catalytic amount of 10% (w/w) Pd/C in absolute EtOH (10 ml) was stirred under H_2_ atmosphere at RT for 12 h. Thereafter, the catalyst was filtered off over Celite and the solvent was distilled under reduced pressure, to afford the products in quantitative yield. The purity and the identity of the final products were determined to be >95% by RP HPLC coupled to ESI MS, by ^1^H and ^13^C NMR, and by 2D gCOSY experiments at 600 MHz in DMSO‐*d*_*6*_.

### 5.12 Analytical characterization of synthesized peptides

H‐Gly‐Gln‐Asn‐Gly‐Ile‐Ser‐Asp‐Leu‐OH (TH1). ^1^H NMR (600 MHz, DMSO‐*d*_6_) δ 8.56 (d, *J* = 7.8 Hz, 1H, GlnNH), 8.37 (d, *J* = 7.7 Hz, 1H, AsnNH), 8.16 (d, *J* = 8.2 Hz, 1H, AspNH), 8.03 (d, *J* = 7.6 Hz, 1H, SerNH), 7.99 (t, *J* = 6.0 Hz, 1H, GlyNH), 7.94 (br s, 2H, GlyNH_2_), 7.87 (d, *J* = 8.0 Hz, 1H, LeuNH), 7.85 (d, *J* = 8.2 Hz, 1H, IleNH),7.38 (br s, 1H, Asn‐CONH_2_), 7.23 (br s, 1H, Gln‐CONH_2_), 6.91 (br s, 1H, Asn‐CONH_2_), 6.77 (br s, 1H, Gln‐CONH_2_), 5.10 (t, *J* = 6.0 Hz, 1H, SerOH), 4.62 (td, *J* = 7.8, 5.4 Hz, 1H, AspHα), 4.57–4.52 (m, 1H, AsnHα), 4.36 (td, *J* = 7.8, 5.7 Hz, 1H, GlnHα), 4.32 (dt, *J* = 7.7, 6.1 Hz, 1H, SerHα), 4.23 (dd, *J* = 8.8, 7.4 Hz, 1H, IleHα), 4.20–4.15 (m, 1H, LeuHα), 3.78 (dd, *J* = 16.9, 5.8 Hz, 1H, GlyHα), 3.71 (dd, *J* = 16.9, 5.8 Hz, 1H, GlyHα), 3.64–3.56 (m, GlyHα+SerHβ), 3.56–3.50 (m, 1H, SerHβ), 2.68 (dd, *J* = 16.7, 5.4 Hz, 1H, AspHβ), 2.60–2.51 (m, 2H, AsnHβ+AspHβ), 2.43 (dd, *J* = 15.5, 7.7 Hz, 1H, AsnHβ), 2.10 (t, *J* = 8.0 Hz, 2H, GlnHγ), 1.88 (dt, *J* = 13.8, 7.5 Hz, 1H, GlnHβ), 1.78–1.70 (m, 2H, GlnHβ+IleHβ), 1.65–1.59 (m, 1H, LeuHγ), 1.53–1.46 (m, 2H, LeuHβ), 1.43–1.37 (m, 1H, IleHγ), 1.08–1.03 (m, 1H, IleHγ), 0.88 (d, *J* = 6.6 Hz, 3H, LeuHδ‐CH_3_), 0.82 (d, *J* = 6.6 Hz, 6H, LeuHδ‐CH_3_+IleHγ‐CH_3_), 0.80 (t, *J* = 7.8 Hz, 3H, IleHδ‐CH_3_). ^13^C NMR (151 MHz, DMSO‐*d*_6_) δ 173.70, 173.64, 171.77, 171.44, 171.05, 170.95, 170.70, 170.45, 169.82, 168.56, 165.85, 61.88, 56.78, 54.79, 52.21, 50.41, 49.81, 49.30, 42.11, 37.06, 36.66, 35.96, 31.15, 28.29, 27.15, 24.27, 24.11, 22.85, 21.37, 15.34, 10.99. ESI MS m/z calcd. for [C_32_H_55_N_10_O_14_]^+^ 803.4, found 803.3 [M + H]^+^.

H‐Gly‐Gln‐Asn‐Asp‐OH (TH16). ^1^H NMR (600 MHz, DMSO‐*d*_6_) δ 8.56 (d, *J* = 8.0 Hz, 1H, GlnNH), 8.33 (d, *J* = 7.8 Hz, 1H, AsnNH), 8.01 (d, *J* = 8.0 Hz, 1H, AspNH), 7.35 (br s, 1H, Asn‐CONH_2_), 7.24 (br s, 1H, Gln‐CONH_2_), 6.89 (br s, 1H, Asn‐CONH_2_), 6.79 (br s, 1H, Gln‐CONH_2_), 4.58 (qd, *J* = 7.9, 4.9 Hz, 1H, AsnHα), 4.45 (q, *J* = 6.9 Hz, 1H, AspHα), 4.37 (td, *J* = 7.9, 5.6 Hz, 1H, GlnHα), 3.59 (d, *J* = 9.4 Hz, 2H, GlyHα), 2.62–2.54 (m, 3H, AspHβ+AsnHβ), 2.42–2.35 (m, 1H, AsnHβ), 2.11 (t, *J* = 8.0 Hz, 2H, GlnHγ), 1.89 (dq, *J* = 14.1, 7.2 Hz, 1H, GlnHβ), 1.75 (dt, *J* = 14.0, 7.6 Hz, 1H, GlnHβ). ^13^C NMR (151 MHz, DMSO‐*d*_6_) δ 173.81, 172.48, 171.90, 171.27, 170.65, 170.63, 165.81, 52.11, 49.58, 48.70, 43.72, 37.10, 34.32, 31.21, 28.45. ESI MS m/z calcd. for [C_15_H_25_N_6_O_9_]^+^ 433.2, found 432.9 [M + H]^+^.

H‐Gly‐Gln‐Asn‐d‐Asp‐OH (TH17). ^1^H NMR (600 MHz, DMSO‐*d*_6_) δ 8.56 (d, *J* = 8.2 Hz, 1H, GlnNH), 8.33 (d, *J* = 7.9 Hz, 1H, AsnNH), 8.06 (d, *J* = 8.0 Hz, 1H, AspNH), 7.98 (br t, *J* = 5.7 Hz, 2H, GlyNH_2_), 7.33 (br s, 1H, Asn‐CONH_2_), 7.18 (br s, 1H, Gln‐CONH_2_), 6.88 (br s, 1H, Asn‐CONH_2_), 6.79 (br s, 1H, Gln‐CONH_2_), 4.61 (td, *J* = 8.1, 5.4 Hz, 1H, AsnHα), 4.51 (dt, *J* = 8.1, 6.0 Hz, 1H, AspHα), 4.39 (td, *J* = 8.1, 5.5 Hz, 1H, GlnHα), 3.59 (br d, *J* = 5.1 Hz, 2H, GlyHα), 2.68 (dd, *J* = 16.7, 5.9 Hz, 1H, AspHβ), 2.60 (dd, *J* = 16.6, 6.1 Hz, 1H, AspHβ), 2.53–2.51 (m, 1H, AsnHβ), 2.35 (dd, *J* = 15.5, 8.2 Hz, 1H, AsnHβ), 2.10 (t, *J* = 8.0 Hz, 2H, GlnHγ), 1.89 (dtd, *J* = 13.5, 7.9, 5.6 Hz, 1H, GlnHβ), 1.74 (dq, *J* = 13.6, 8.0 Hz, 1H, GlnHβ). ^13^C NMR (151 MHz, DMSO‐*d*_6_) δ 173.73, 172.13, 171.73, 171.12, 170.61, 170.58, 165.73, 51.99, 49.47, 48.58, 43.73, 37.22, 36.01, 31.20, 28.51. ESI MS m/z calcd. for [C_15_H_25_N_6_O_9_]^+^ 433.2, found 432.9 [M + H]^+^.

H‐Gly‐Gln‐Asn‐d‐Asp‐OBn (TH7). ^1^H NMR (600 MHz, DMSO‐*d*_6_) δ 8.54 (d, *J* = 8.2 Hz, 1H, GlnNH), 8.32 (d, *J* = 7.9 Hz, 1H, AsnNH), 8.29 (d, *J* = 8.0 Hz, 1H, AspNH), 7.40–7.30 (m, 6H, Bn‐aromatic+Asn‐CONH_2_), 7.20 (br s, 1H, Gln‐CONH_2_), 6.89 (br s, 1H, Asn‐CONH_2_), 6.80 (br s, 1H, Gln‐CONH_2_), 5.13 (d, *J* = 12.6 Hz, 1H, Bn‐CH_2_), 5.09 (d, *J* = 12.6 Hz, 1H, Bn‐CH_2_), 4.67–4.58 (m, 2H, AspHα+AsnHα), 4.37 (td, *J* = 8.2, 5.5 Hz, 1H, GlnHα), 3.59 (s, 2H, GlyHα), 2.73 (dd, *J* = 16.9, 6.1 Hz, 1H, AspHβ), 2.64 (dd, *J* = 16.8, 6.2 Hz, 1H, AspHβ), 2.49–2.46 (m, 1H, AsnHβ), 2.37 (dd, *J* = 15.5, 8.3 Hz, 1H, AsnHβ), 2.10 (t, *J* = 8.0 Hz, 2H, GlnHγ), 1.93–1.85 (m, 1H, GlnHβ), 1.74 (dq, *J* = 13.4, 8.0 Hz, 1H, GlnHβ). ^13^C NMR (151 MHz, DMSO‐*d*_6_) δ 173.69, 171.67, 171.05, 170.86, 170.61, 170.54, 165.81, 135.84, 128.41, 127.94, 127.57, 66.15, 52.07, 49.53, 48.74, 43.72, 37.20, 35.91, 31.24, 28.49. ESI MS m/z calcd. for [C_22_H_31_N_6_O_9_]^+^ 523.2, found 523.2 [M + H]^+^.

c[Gly‐Gln‐Asn‐isoAsp] (TH2). ^1^H NMR (600 MHz, DMSO‐*d*_6_) δ 8.40 (t, *J* = 6.0 Hz, 1H, GlyNH), 8.06 (d, *J* = 7.7 Hz, 1H, AspNH), 7.56 (d, *J* = 8.6 Hz, 1H, GlnNH), 7.31 (br s, 1H, Asn‐CONH_2_), 7.22 (br s, 1H, Gln‐CONH_2_), 6.90 (d, *J* = 8.9 Hz, 1H, AsnNH), 6.76 (br s, 1H, Gln‐CONH_2_), 6.74 (br s, 1H, Asn‐CONH_2_), 4.57 (dt, *J* = 8.9, 5.3 Hz, 1H, AsnHα), 4.40 (td, *J* = 7.8, 5.6 Hz, 1H, AspHα), 4.09 (td, *J* = 8.6, 6.7 Hz, 1H, GlnHα), 3.66 (dd, *J* = 14.6, 6.3 Hz, 1H, GlyHα), 3.48 (dd, *J* = 15.0, 6.0 Hz, 1H, GlyHα), 2.81–2.72 (m, 2H, AsnHβ+AspHβ), 2.56 (dd, *J* = 15.5, 5.1 Hz, 1H, AsnHβ), 2.30 (dd, *J* = 15.4, 5.7 Hz, 1H, AspHβ), 2.04 (dt, *J* = 9.0, 6.0 Hz, 2H, GlnHγ), 1.79–1.66 (m, 2H, GlnHβ). ^13^C NMR (151 MHz, DMSO‐*d*_6_) δ 173.30, 172.28, 171.81, 170.93, 170.36, 169.45, 169.32, 54.04, 49.58, 48.47, 44.17, 36.87, 35.45, 31.39, 27.33. ESI MS m/z calcd. for [C_15_H_23_N_6_O_8_]^+^ 415.4, found 415.4 [M + H]^+^.

c[Gly‐Gln‐Asn‐isoAsp(OBn)] (TH10). ^1^H NMR (600 MHz, DMSO‐*d*_6_) δ 8.45 (t, *J* = 6.1 Hz, 1H, GlyNH), 8.04 (d, *J* = 7.7 Hz, 1H, AspNH), 7.62 (d, *J* = 8.6 Hz, 1H, GlnNH), 7.38–7.30 (m, 6H, Bn‐aromatic+Asn‐CONH_2_), 7.23 (br s, 1H, Gln‐CONH_2_), 7.14 (d, *J* = 8.9 Hz, 1H, AsnNH), 6.77 (br s, 1H, Asn‐CONH_2_), 6.74 (br s, 1H, Gln‐CONH_2_), 5.13 (s, 2H, Bn‐CH_2_), 4.76 (dt, *J* = 8.9, 5.5 Hz, 1H, AsnHα), 4.40 (td, *J* = 7.8, 5.8 Hz, 1H, AspHα), 4.09 (td, *J* = 8.7, 6.7 Hz, 1H, GlnHα), 3.72 (dd, *J* = 14.5, 6.5 Hz, 1H, GlyHα), 3.48–3.46 (m, 1H, GlyHα), 2.80 (dd, *J* = 15.5, 6.1 Hz, 1H, AsnHβ), 2.74 (dd, *J* = 15.5, 7.9 Hz, 1H, AspHβ), 2.61 (dd, *J* = 15.5, 4.9 Hz, 1H, AsnHβ), 2.32 (dd, *J* = 15.4, 5.8 Hz, 1H, AspHβ), 2.09–2.00 (m, 2H, GlnHγ), 1.77 (ddt, *J* = 13.6, 9.1, 6.7 Hz, 1H, GlnHβ), 1.70 (ddt, *J* = 15.3, 8.9, 4.3 Hz, 1H, GlnHβ). ^13^C NMR (151 MHz, DMSO‐*d*_6_) δ 173.32, 171.80, 171.00, 170.72, 170.18, 169.56, 169.42, 135.90, 128.41, 127.94, 127.59, 64.91, 54.02, 49.71, 48.83, 44.18, 36.92, 35.40, 31.39, 27.27. ESI MS m/z calcd. for [C_22_H_29_N_6_O_8_]^+^ 505.2, found 505.2 [M + H]^+^.

c[Gly‐Gln‐Asn‐Asp(OBn)] (TH15). ^1^H NMR (600 MHz, DMSO‐*d*_6_) δ 8.48 (t, *J* = 6.2 Hz, 1H, GlyNH), 8.06 (d, *J* = 7.7 Hz, 1H, AspNH), 7.65 (d, *J* = 8.6 Hz, 1H, GlnNH), 7.38–7.30 (m, 6H, Bn‐aromatic+Asn‐CONH_2_), 7.22 (br s, 1H, Gln‐CONH_2_), 7.13 (d, *J* = 8.8 Hz, 1H, AsnNH), 6.76 (br s, 1H, Asn‐CONH_2_), 6.74 (br s, 1H, Gln‐CONH_2_), 5.13 (s, 2H, Bn‐CH_2_), 4.75 (dt, *J* = 9.0, 5.5 Hz, 1H, AsnHα), 4.40 (td, *J* = 7.8, 5.7 Hz, 1H, AspHα), 4.09 (td, *J* = 8.6, 6.7 Hz, 1H, GlnHα), 3.71 (dd, *J* = 14.5, 6.5 Hz, 1H, GlyHα), 3.46 (dd, *J* = 14.4, 6.0 Hz, 1H, GlyHα), 2.80 (dd, *J* = 15.5, 6.1 Hz, 1H, AsnHβ), 2.74 (dd, *J* = 15.6, 7.8 Hz, 1H, AspHβ), 2.63–2.60 (m, 1H, AsnHβ), 2.32 (dd, *J* = 15.6, 6.0 Hz, 1H, AspHβ), 2.04 (dt, *J* = 8.9, 6.0 Hz, 2H, GlnHγ), 1.79–1.67 (m, 2H, GlnHβ). ^13^C NMR (151 MHz, DMSO‐*d*_6_) δ 173.32, 171.80, 171.01, 170.71, 170.17, 169.57, 169.43, 135.91, 128.41, 127.94, 127.59, 66.12, 54.03, 49.69, 48.83, 44.18, 36.92, 35.42, 31.39, 27.27. ESI MS m/z calcd. for [C_22_H_29_N_6_O_8_]^+^ 505.2, found 505.2 [M + H]^+^.

c[Gly‐Gln‐Asn‐d‐isoAsp] (TH3). ^1^H NMR (600 MHz, DMSO‐*d*_6_) δ 8.51 (t, *J* = 6.1 Hz, 1H, GlyNH), 7.70–7.63 (m, 2H, AspNH+AsnNH), 7.61 (d, *J* = 9.6 Hz, 1H, GlnNH), 7.44 (br s, 1H, Asn‐CONH_2_), 7.25 (br s, 1H, Gln‐CONH_2_), 6.91 (br s, 1H, Asn‐CONH_2_), 6.76 (br s, 1H, Gln‐CONH_2_), 4.51 (td, *J* = 9.6, 4.8 Hz, 1H, AsnHα), 4.28 (ddd, *J* = 11.8, 7.4, 4.2 Hz, 1H, AspHα), 4.18 (td, *J* = 9.4, 7.0 Hz, 1H, GlnHα), 3.67 (dd, *J* = 15.8, 6.0 Hz, 1H, GlyHα), 3.55 (dd, *J* = 15.8, 6.1 Hz, 1H, GlyHα), 2.63 (dd, *J* = 14.2, 4.2 Hz, 1H, AspHβ), 2.55–2.51 (m, 1H, AspHβ), 2.49–2.46 (m, 1H, AsnHβ), 2.33 (dd, *J* = 14.8, 9.8 Hz, 1H, AsnHβ), 2.04 (dt, *J* = 8.6, 6.0 Hz, 2H, GlnHγ), 1.84–1.73 (m, 2H, GlnHβ). ^13^C NMR (151 MHz, DMSO‐*d*_6_) δ 173.27, 172.61, 171.60, 170.69, 169.64, 169.18, 169.14, 54.26, 50.82, 50.05, 43.47, 37.25, 36.26, 31.21, 26.73. ESI MS m/z calcd. for [C_15_H_23_N_6_O_8_]^+^ 415.4, found 415.4 [M + H]^+^.

H‐Gly‐Gln‐Asn‐Asp(OBn)‐OH (TH14). ^1^H NMR (600 MHz, DMSO‐*d*_6_) δ 8.54 (d, *J* = 8.0 Hz, 1H, GlnNH), 8.29 (d, *J* = 7.9 Hz, 1H, AsnNH), 8.23 (d, *J* = 8.0 Hz, 1H, AspNH), 7.40–7.27 (m, 6H, Bn‐aromatic+Asn‐CONH_2_), 7.23 (br s, 1H, Gln‐CONH_2_), 6.89 (br s, 1H, Asn‐CONH_2_), 6.79 (br s, 1H, Gln‐CONH_2_), 5.10 (br d, *J* = 2.3 Hz, 2H, Bn‐CH_2_), 4.64 (q, *J* = 6.5 Hz, 1H, AspHα), 4.59 (td, *J* = 8.1, 5.1 Hz, 1H, AsnHα), 4.33 (q, *J* = 7.4 Hz, 1H, GlnHα), 3.57 (s, 2H, GlyHα), 2.65 (d, *J* = 6.0 Hz, 2H, AspHβ), 2.55–2.52 (m, 1H, AsnHβ), 2.38 (dd, *J* = 15.4, 8.6 Hz, 1H, AsnHβ), 2.10 (t, *J* = 8.0 Hz, 2H, GlnHγ), 1.88 (dq, *J* = 14.3, 7.6 Hz, 1H, GlnHβ), 1.74 (dt, *J* = 14.1, 7.8 Hz, 1H, GlnHβ). ^13^C NMR (151 MHz, DMSO‐*d*_6_) δ 173.74, 171.81, 171.18, 170.96, 170.75, 170.65, 165.94, 135.89, 128.42, 127.95, 127.60, 66.11, 54.92, 52.16, 49.47, 48.82, 37.07, 36.09, 31.22, 28.39. ESI MS m/z calcd. for [C_22_H_31_N_6_O_9_]^+^ 523.2, found 523.2 [M + H]^+^.

c[Gly‐Gln‐Asn‐d‐isoAsp(OBn)] (TH11). ^1^H NMR (600 MHz, DMSO‐*d*_6_) δ 8.53 (t, *J* = 6.1 Hz, 1H, GlyNH), 7.83 (d, *J* = 7.2 Hz, 1H, AspNH), 7.72–7.60 (m, 2H, AsnNH+GlnNH), 7.45 (br s, 1H, Asn‐CONH_2_), 7.39–7.24 (m, 6H, Bn‐aromatic+Gln‐CONH_2_), 6.92 (br s, 1H, Asn‐CONH_2_), 6.76 (br s, 1H, Gln‐CONH_2_), 5.15 (d, *J* = 12.6 Hz, 1H, Bn‐CH_2_), 5.12 (d, *J* = 12.6 Hz, 1H, Bn‐CH_2_), 4.52 (td, *J* = 9.4, 4.9 Hz, 1H, AsnHα), 4.41 (ddd, *J* = 11.7, 7.2, 4.0 Hz, 1H, AspHα), 4.18 (q, *J* = 8.6 Hz, 1H, GlnHα), 3.68 (dd, *J* = 15.8, 6.1 Hz, 1H, GlyHα), 3.53 (dd, *J* = 15.9, 6.2 Hz, 1H, GlyHα), 2.67 (dd, *J* = 14.2, 4.1 Hz, 1H, AspHβ), 2.59–2.54 (m, 1H, AspHβ), 2.48–2.45 (m, 1H, AsnHβ), 2.31 (dd, *J* = 14.8, 9.5 Hz, 1H, AsnHβ), 2.03 (q, *J* = 6.9 Hz, 2H, GlnHγ), 1.77 (q, *J* = 7.9 Hz, 2H, GlnHβ). ^13^C NMR (151 MHz, DMSO‐*d*_6_) δ 173.25, 171.76, 170.91, 170.72, 169.71, 169.13, 168.83, 135.83, 128.45, 128.06, 127.77, 66.17, 54.23, 50.78, 50.27, 43.52, 37.13, 36.06, 31.20, 26.71. ESI MS m/z calcd. for [C_22_H_29_N_6_O_8_]^+^ 505.2, found 505.2 [M + H]^+^.

H‐Gly‐Gln‐Asn‐Asp‐OBn (TH6). ^1^H NMR (600 MHz, DMSO‐*d*_6_) δ 8.54 (d, *J* = 8.1 Hz, 1H, GlnNH), 8.33–8.25 (m, 2H, AsnNH+AspNH), 7.42–7.27 (m, 6H, Bn‐aromatic+Asn‐CONH_2_), 7.22 (br s, 1H, Gln‐CONH_2_), 6.90 (br s, 1H, Asn‐CONH_2_), 6.80 (br s, 1H, Gln‐CONH_2_), 5.11 (d, *J* = 12.6 Hz, 1H, Bn‐CH_2_), 5.09 (d, *J* = 12.6 Hz, 1H, Bn‐CH_2_), 4.65 (dt, *J* = 8.1, 6.2 Hz, 1H, AspHα), 4.60 (td, *J* = 8.1, 4.9 Hz, 1H, AsnHα), 4.36 (td, *J* = 8.0, 5.6 Hz, 1H, GlnHα), 3.58 (s, 2H, GlyHα), 2.67 (qd, *J* = 16.8, 6.1 Hz, 2H, AspHβ), 2.54–2.51 (m, 1H, AsnHβ), 2.38 (dd, *J* = 15.7, 8.5 Hz, 1H, AsnHβ), 2.10 (t, *J* = 8.0 Hz, 2H, GlnHγ), 1.88 (dtd, *J* = 16.1, 7.6, 5.7 Hz, 1H, GlnHβ), 1.73 (dq, *J* = 13.4, 8.0 Hz, 1H, GlnHβ). ^13^C NMR (151 MHz, DMSO‐*d*_6_) δ 173.73, 171.63, 171.08, 171.02, 170.66, 170.59, 165.80, 135.86, 128.41, 127.96, 127.61, 66.14, 52.02, 49.43, 48.75, 40.06, 37.11, 35.89, 31.21, 28.50. ESI MS m/z calcd. for [C_22_H_31_N_6_O_9_]^+^ 523.2, found 523.2 [M + H]^+^.

H‐Lys‐Ser‐Asp‐d‐Leu‐OH (TH19). ^1^H NMR (600 MHz, DMSO‐*d*_6_) δ 8.66 (d, *J* = 7.9 Hz, 1H, SerNH), 8.45 (d, *J* = 7.7 Hz, 1H, AspNH), 8.19 (d, *J* = 5.0 Hz, 2H, Lys‐αNH_2_), 7.96 (d, *J* = 8.2 Hz, 1H, LeuNH), 7.82 (t, *J* = 5.8 Hz, 2H, Lys‐ɛNH_2_), 4.60 (td, *J* = 8.0, 4.9 Hz, 1H, AspHα), 4.44 (q, *J* = 6.6 Hz, 1H, SerHα), 4.21 (ddd, *J* = 10.3, 8.1, 4.6 Hz, 1H, LeuHα), 3.85 (q, *J* = 5.9 Hz, 1H, LysHα), 3.69 (dd, *J* = 10.5, 6.0 Hz, 1H, SerHβ), 3.50 (dd, *J* = 10.5, 6.8 Hz, 1H, SerHβ), 2.80–2.75 (m, 2H, LysHɛ), 2.72–2.67 (m, 1H, AspHβ), 2.55 (dd, *J* = 16.7, 8.3 Hz, 1H, AspHβ), 1.71 (dt, *J* = 10.9, 6.6 Hz, 2H, LysHβ), 1.64–1.51 (m, 4H, LysHδ+LeuHγ+LeuHβ), 1.48–1.43 (m, 1H, LeuHβ), 1.38–1.32 (m, 2H, LysHγ), 0.85 (d, *J* = 6.3 Hz, 3H, LeuHδ‐CH_3_), 0.80 (d, *J* = 6.4 Hz, 3H, LeuHδ‐CH_3_). ^13^C NMR (151 MHz, DMSO‐*d*_6_) δ 173.87, 171.73, 170.42, 169.81, 168.64, 62.04, 54.68, 52.00, 50.43, 49.82, 38.61, 36.22, 30.59, 26.57, 24.22, 22.96, 21.22, 21.20. ESI MS m/z calcd. for [C_19_H_36_N_5_O_8_]^+^ 462.2, found 462.0 [M + H]^+^.

H‐Lys‐Ser‐Asp‐Leu‐OH (TH18). ^1^H NMR (600 MHz, DMSO‐*d*_6_) δ 8.65 (d, *J* = 7.8 Hz, 1H, SerNH), 8.38 (d, *J* = 8.1 Hz, 1H, AspNH), 8.16 (d, *J* = 5.4 Hz, 2H, Lys‐αNH_2_), 7.95 (d, *J* = 8.0 Hz, 1H, LeuNH), 7.78 (t, *J* = 5.9 Hz, 2H, Lys‐ɛNH_2_), 4.64 (td, *J* = 8.1, 5.0 Hz, 1H, AspHα), 4.42 (dt, *J* = 8.0, 6.2 Hz, 1H, SerHα), 4.18 (ddd, *J* = 9.9, 8.0, 5.1 Hz, 1H, LeuHα), 3.86–3.83 (m, 1H, LysHα), 3.66 (dd, *J* = 10.7, 6.1 Hz, 1H, SerHβ), 3.57 (dd, *J* = 10.7, 6.3 Hz, 1H, SerHβ), 2.76 (dt, *J* = 10.2, 5.9 Hz, 2H, LysHɛ), 2.70 (dd, *J* = 16.8, 5.0 Hz, 1H, AspHβ), 2.57–2.51 (m, 1H, AspHβ), 1.73–1.62 (m, 2H, LysHβ), 1.60–1.44 (m, 5H, LysHδ+LeuHγ+LeuHβ), 1.41–1.30 (m, 2H, LysHγ), 0.88 (d, *J* = 6.6 Hz, 3H, LeuHδ‐CH_3_), 0.83 (d, *J* = 6.5 Hz, 3H, LeuHδ‐CH_3_). ^13^C NMR (151 MHz, DMSO‐*d*_6_) δ 173.72, 171.69, 170.52, 169.53, 168.49, 64.94, 54.75, 51.89, 50.45, 49.38, 38.52, 35.98, 30.53, 26.49, 24.18, 22.88, 21.35, 21.10. ESI MS m/z calcd. for [C_19_H_36_N_5_O_8_]^+^ 462.2, found 462.0 [M + H]^+^.

c[isoLys‐Ser‐Asp‐d‐Leu] (TH5). ^1^H NMR (600 MHz, DMSO‐*d*_6_) δ 8.75 (br s, 1H, SerNH), 8.20 (d, *J* = 7.9 Hz, 1H, AspNH), 7.62 (d, *J* = 9.3 Hz, 1H, LeuNH), 7.56 (br d, *J* = 6.9 Hz, 1H, Lys‐ɛNH), 4.36–4.17 (m, 3H, SerHα+AspHα+LeuHα), 3.72 (dd, *J* = 11.3, 5.5 Hz, 1H, SerHβ), 3.64 (dd, *J* = 8.7, 4.0 Hz, 1H, LysHα), 3.58 (dd, *J* = 11.4, 3.9 Hz, 1H, SerHβ), 3.35–3.30 (m, 1H, LysHɛ), 2.80–2.72 (m, 1H, LysHɛ), 2.65 (dd, *J* = 16.2, 5.3 Hz, 1H, AspHβ), 2.40 (dd, *J* = 16.2, 5.1 Hz, 1H, AspHβ), 1.68–1.64 (m, 2H, LeuHβ+LysHβ), 1.57–1.45 (m, 4H, LysHβ+LeuHγ+LysHδ+LeuHβ), 1.40–1.25 (m, 3H, LysHδ+LysHγ), 0.86 (d, *J* = 6.5 Hz, 3H, LeuHδ‐CH3), 0.80 (d, *J* = 6.4 Hz, 3H, LeuHδ‐CH3). ^13^C NMR (151 MHz, DMSO‐*d*_6_) δ 173.72, 172.89, 171.45, 170.83, 170.16, 64.91, 56.71, 52.94, 50.62, 50.33, 37.85, 37.71, 32.13, 26.83, 24.23, 23.32, 21.07, 20.78. ESI MS m/z calcd. for [C_19_H_34_N_5_O_7_]^+^ 444.2, found 444.1 [M + H]^+^.

c[isoLys‐Ser‐Asp‐Leu] (TH4). ^1^H NMR (600 MHz, DMSO‐*d*_6_) δ 8.51 (d, *J* = 7.6 Hz, 1H, LeuNH), 8.36 (br s, 1H, SerNH), 8.32 (br s, 1H, AspNH), 8.04 (t, *J* = 5.6 Hz, 1H, Lys‐ɛNH), 4.26–4.16 (m, 2H, SerHα+AspHα), 3.99 (ddd, *J* = 11.2, 7.5, 4.6 Hz, 1H, LeuHα), 3.68 (dd, *J* = 11.3, 5.6 Hz, 1H, SerHβ), 3.59 (dd, *J* = 11.5, 4.8 Hz, 1H, SerHβ), 3.25 (dd, *J* = 8.8, 3.6 Hz, 1H, LysHα), 3.20–3.12 (m, 1H, LysHɛ), 2.92–2.83 (m, 1H, LysHɛ), 2.59 (dd, *J* = 15.6, 4.9 Hz, 1H, AspHβ), 2.21 (dd, *J* = 15.5, 5.3 Hz, 1H, AspHβ), 1.66–1.61 (m, 1H, LeuHγ), 1.56–1.48 (m, 3H, LeuHβ+LysHβ), 1.41–1.31 (m, 3H, LysHβ+LysHδ), 1.26–1.13 (m, 2H, LysHγ), 0.85 (d, *J* = 6.5 Hz, 3H, LeuHδ‐CH3), 0.78 (d, *J* = 6.4 Hz, 3H, LeuHδ‐CH3). ^13^C NMR (151 MHz, DMSO‐*d*_6_) δ 173.35, 172.82, 171.82, 171.58, 170.13, 64.90, 56.13, 54.22, 52.50, 51.51, 38.67, 36.13, 28.44, 24.29, 23.13, 21.93, 21.07, 17.26. ESI MS m/z calcd. for [C_19_H_34_N_5_O_7_]^+^ 444.2, found 444.1 [M + H]^+^.

### 5.13 Molecular modeling

Plausible structures of the cyclopeptides were obtained by simulated annealing using the AMBER force field in a 30 Å × 30 Å × 30 Å box of standard TIP3P models of equilibrated water, periodic boundary conditions dielectric scale factor = 1, and cutoff for the nonbonded interactions = 12 Å; all water molecules closer than 2.3 Å to a solute atom were eliminated. 100 random structures were generated by a 100 ps simulation at 1200 K; then the system was cooled in 20 ps to 50 K. The resulting structures were minimized by 3000 cycles of steepest descent and 3000 cycles of conjugated gradient, convergence = 0.01 kcal Å^−1^ mol^−1^. The backbones of the structures were clustered by the rmsd analysis.

For the Molecular Dynamics simulations of TH2‐Y^9^NLLKEEQTPQ^19^, the cyclopeptide TH2 was initially superimposed to the G^295^QNG^298^ turn, then the C‐terminal sequence was removed. The system was subjected to a 1000 ps molecular dynamics simulations keeping the Y^9^NLLKEEQTPQ^19^ sequence fully restrained, force constant = 7 kcal mol^−1^ Å^−2^, for distances, 16 kcal mol^−1^ Å^−2^ for dihedral angles. The simulation was conducted at 300 K and 1 atm by using the AMBER force field in a 60 Å × 60 Å × 60 Å box of standard TIP3P models of equilibrated water, periodic boundary conditions dielectric scale factor = 1, and cutoff for the nonbonded interactions = 12 Å; all water molecules closer than 2.3 Å to a solute atom were eliminated. Then the system was subjected to a 1000 ps simulation with the peptide restrained by a 50% scaled force field, followed by a 1000 ps unrestrained simulation. The resulting structure was minimized by 3000 cycles of steepest descent and 3000 cycles of conjugated gradient, and convergence = 0.01 kcal Å^−1^ mol^−1^.

## AUTHOR CONTRIBUTIONS

**Alessandra Stefan:** Investigation. **Luca Gentilucci:** Conceptualization; funding acquisition. **Francesca Ruffolo:** Investigation. **Valentina Rossi:** Investigation. **Sofia Sordi:** Investigation. **Federica Santino:** Investigation. **Maurizio Brigotti:** Investigation. **Claudia Scotti:** Investigation. **Luisa Iamele:** Investigation. **Hugo de Jonge:** Investigation. **Fabrizio Dal Piaz:** Investigation. **Danilo Rocco Santarcangelo:** Investigation. **Alejandro Hochkoeppler:** Conceptualization; writing – original draft; writing – review and editing; supervision; funding acquisition; data curation. **Tingting He:** Investigation. **Giuseppina di Stefano:** Investigation.

## CONFLICT OF INTEREST STATEMENT

The authors declare no conflict of interest.

## Supporting information


**DATA S1.** Supporting Information.
