# Supplementary material for: Peptides inhibiting the assembly of monomeric human l‐lactate dehydrogenase into catalytically active homotetramer decrease the synthesis of lactate in cultured cells
Source: Protein Sci. 2024 Sep 14;33(10):e5161. doi: 10.1002/pro.5161 (PMC11400633; doi:10.1002/pro.5161)
Supplement: Supplementary file 1 — DATA S1. Supporting Information. [file PRO-33-e5161-s001.pdf]

Supplementary data for:

Peptides inhibiting the assembly of monomeric human L-lactate dehydrogenase into catalytically-active homotetramer decrease the synthesis of lactate cultured cells

Alessandra Stefan°$, Luca Gentilucci£, Francesca Ruffolo°, Valentina Rossi#, Sofia Sordi°, Tingting He£, Giuseppina Di Stefano#, Federica Santino£, Maurizio Brigotti#, Claudia Scotti§, Luisa Iamele§, Hugo de Jonge§, Fabrizio Dal Piaz¶, Danilo Rocco Santarcangelo£, Alejandro Hochkoeppler°$*

°Department of Pharmacy and Biotechnology, University of Bologna, Viale Risorgimento 4, 40136 Bologna (Italy)

$CSGI, University of Firenze, Via della Lastruccia 3, 50019 Sesto Fiorentino, FI (Italy)

£Department of Chemistry "Giacomo Ciamician", University of Bologna, Via Selmi 2, 40126 Bologna (Italy)

#Department of Medical and Surgical Sciences, University of Bologna, Via San Giacomo 14, 40126 Bologna (Italy)

§Department of Molecular Medicine, University of Pavia, Via Forlanini 6, 27100 Pavia (Italy)

¶Department of Medicine, University of Salerno, Via Giovanni Paolo II 132, 84084 Fisciano, Italy

*Corresponding Author. e-mail: a.hochkoeppler@unibo.it

Table of contents:

Figures S1-S4	pS2-S5
Tables ST1-ST2	pS6
Figures S5A-S5Q	pS7-S21
Figures S6A-S6C	pS22-S24

CATATGGCGACCCTGAAAGACCAACTGATTTACAACCTGCTGAAAGAGGAACA
GACCCCGCAAAACAAGATTACCGTGGTGGGCGTGGGCGCGGTTGGTATGGCGT
GCGCGATCAGCATTCTGATGAAGGACCTGGCGGATGAACTGGCGCTGGTGGAC
GTTATCGAAGATAAGCTGAAAGGCGAGATGATGGACCTGCAGCACGGCAGCCT
GTTCCTGCGTACCCCGAAGATTGTGAGCGGCAAAGATTACAACGTTACCGCGA
ACAGCAAGCTGGTGATCATTACCGCGGGTGCGCGTCAGCAAGAAGGCGAGAGC
CGTCTGAACCTGGTGCAACGTAACGTTAACATCTTCAAGTTCATCATTCCGAA
CGTGGTTAAGTACAGCCCGAACTGCAAACTGCTGATCGTGAGCAACCCGGTTG
ACATTCTGACCTATGTTGCGTGGAAGATCAGCGGTTTCCCGAAAAACCGTGTG
ATTGGTAGCGGCTGCAACCTGGATAGCGCGCGTTTTCGTTATCTGATGGGTGA
ACGTCTGGGCGTTCATCCGCTGAGCTGCCATGGTTGGGTTCTGGGCGAGCATG
GTGACAGCAGCGTGCCGGTTTGGAGCGGTATGAACGTGGCGGGCGTTAGCCTG
AAAACCCTGCACCCGGATCTGGGCACCGACAAGGATAAAGAACAGTGGAAAGA
GGTGCACAAACAAGTGGTTGAAAGCGCGTACGAGGTGATCAAGCTGAAAGGTT
ATACCAGCTGGGCGATTGGCCTGAGCGTGGCGGACCTGGCGGAGAGCATCATG
AAGAACCTGCGTCGTGTGCACCCGGTTAGCACCATGATCAAGGGTCTGTACGG
CATTAAAGACGATGTGTTTCTGAGCGTTCCGTGCATCCTGGGTCAGAACGGCA
TTAGCGATCTGGTGAAAGTTACCCTGACCAGCGAGGAAGAGGCGCGTCTGAAA
AAGAGCGCGGACACCCTGTGGGGCATCCAAAAGGAACTGCAATTCTAA**GGATC**
C

MATLKDQLIYNLLKEEQTPQNKITVVGVGAVGMACAISILMKDLADELALVDV
IEDKLKGEMMDLQHGSLFLRTPKIVSGKDYNVTANSKLVIITAGARQQEGESR
LNLVQRNVNIFKFIIPNVVKYSPNCKLLIVSNPVDILTYVAWKISGFPKNRVI
GSGCNLDSARFRYLMGERLGVHPLSCHGWVLGEHGDSSVPVWSGMNVAGVSLK
TLHPDLGTDKDKEQWKEVHKQVVESAYEVIKLKGYTSWAIGLSVADLAESIMK
NLRRVHPVSTMIKGLYGIKDDVFLSVPCILGQNGISDLVKVTLTSEEEARLKK
SADTLWGIQKELQF

Supplementary Figure S1. Synthetic gene coding for human L-lactate dehydrogenase A (SwissProt Code: P00338).
Sequence of the synthetic gene, optimized for the *Escherichia coli* codon usage, coding for human L-lactate dehydrogenase A. Start and stop codons are underlined. *Nde*I and *BamH*I sites are marked in green and blue, respectively. The corresponding translation is also shown.

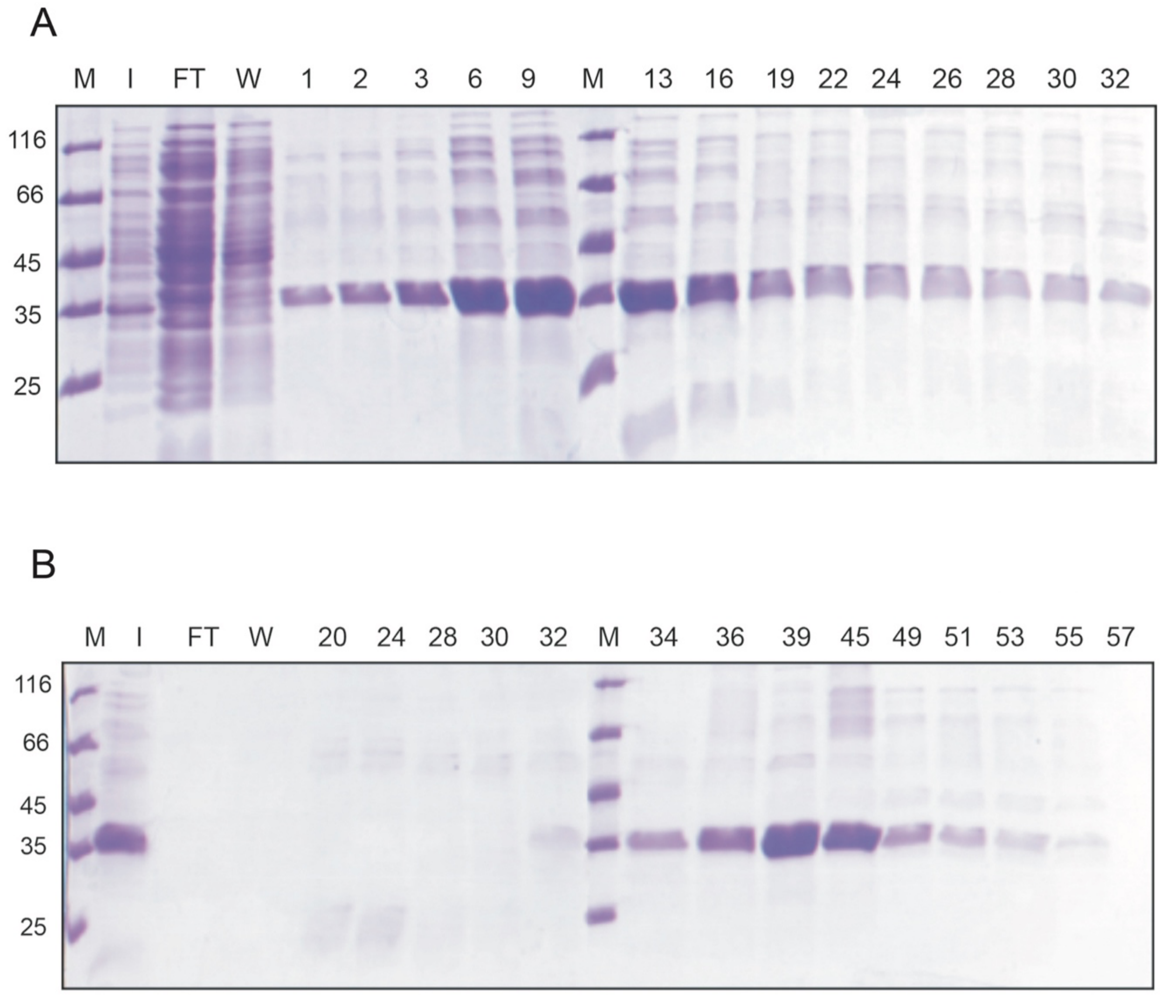

Supplementary Figure S2. SDS-PAGE analysis of the purification of monomeric human LDH-A by means of affinity and hydrophobic interaction chromatography.

(A, B) Electrophoretic analysis of fractions eluted from the Cibacron Blue (A) and HiTrap Phenyl (B) columns used to perform the first two purification steps of monomeric human LDH-A. M: molecular mass markers (their M_r is indicated in kDa at the left); I: input; FT: flow through; W: wash; the fraction numbers are indicated on the top.

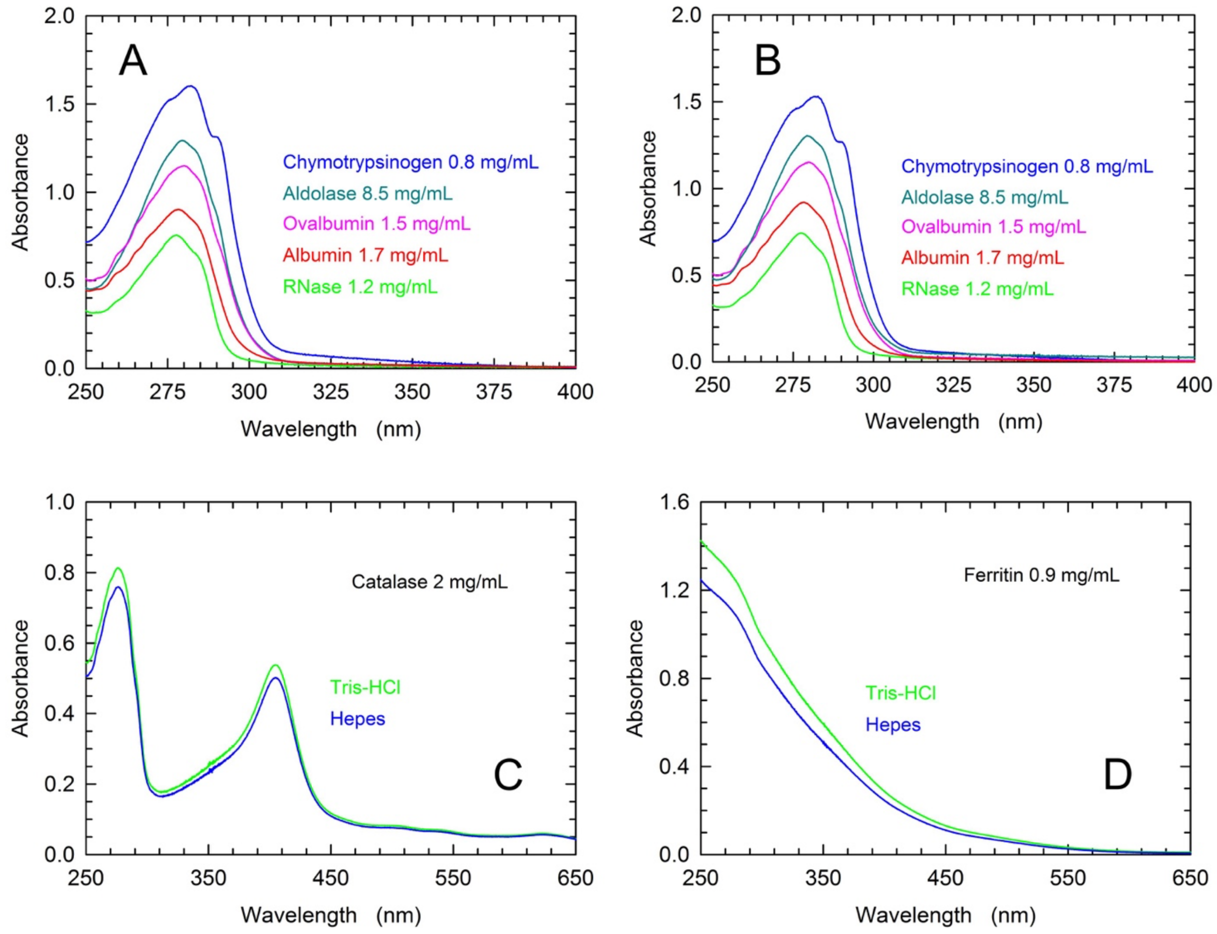

Supplementary Figure S3. Absorption spectra of the molecular mass markers used to calibrate the Superdex 200 column utilized for gel filtration experiments (see Figure 1).

(A, B) Absorption spectra of the indicated molecular mass markers dissolved in 10 mM Tris-HCl (A) or in 10 mM HEPES (B). Both buffers were poised at pH 7.5. (C, D) Absorption spectra of catalase (C) and ferritin (D) dissolved in 10 mM Tris-HCl (green lines) or in 10 mM HEPES (blue lines). Both buffers were poised at pH 7.5. The spectra of ferritin are in agreement with those reported by May and Fish (May ME, Fish WW, 1978, The UV and visible spectral properties of ferritin. Arch Biochem Biophys 190:720-725).

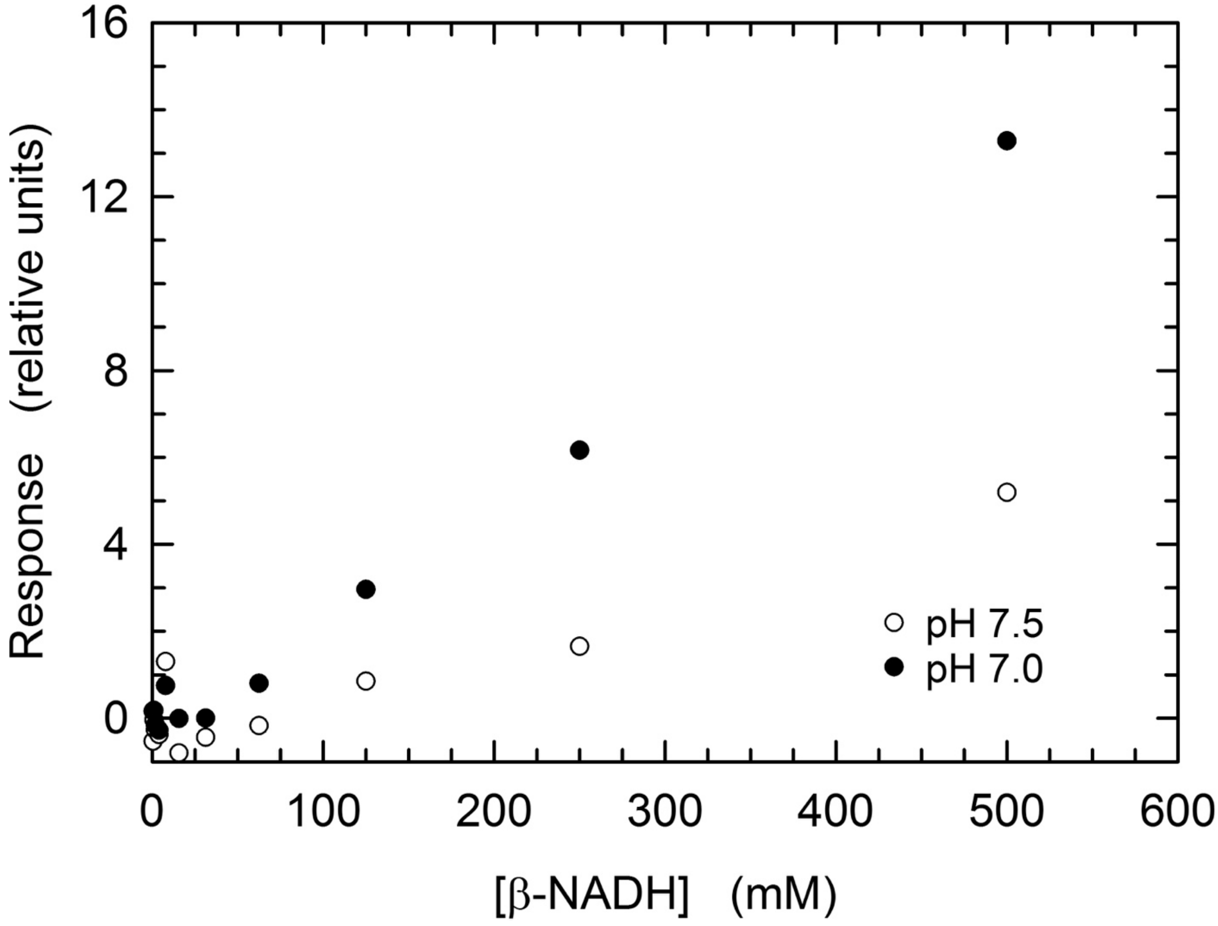

Supplementary Figure S4. Binding of β-NADH to monomeric human LDH-A. The association of β-NADH to human LDH-A in monomeric form was evaluated by Surface Plasmon Resonance (SPR) at pH 7.5 (white circles) and 7.0 (black circles). Other conditions as in Figure 4.

Table ST1. RP HPLC and ESI MS analyses of the linear precursors and of the cyclopeptides considered. Reaction yields are also shown.

Protected linear peptides[a]				
Compound	Sequence	Yield (%)[e]	Purity (%)[c]	ESI-MS [M+1]+ [d]
TH6	GQND-OBn	78	98	523.2/523.2
TH7	GQN-(*R*)-D-OBn	75	97	523.2/523.2
TH8	Fmoc-KSD(OBn)L	80[b]	89	773.8/774.4
TH9	Fmoc-KSD(OBn)-(*R*)-L	77[b]	85	773.8/774.4
TH14	GQND(OBn)	78	97	523.2/523.2
**Protected cyclic peptides**				
Compound	Sequence	Yield (%)[e]	Purity (%)[c]	ESI-MS [M+1]+ [d]
TH10	c[GQN-isoD(OBn)]	68	96	505.2/505.2
TH11	c[GQN-(*R*)-isoD(OBn)]	64	96	505.2/505.2
TH12	c[isoK(Fmoc)-SDL]	59	82	666.3/666.3
TH13	c[isoK(Fmoc)-SD-(*R*)-L]	55	80	666.3/666.3
TH15	c[GQND(OBn)]	68	96	505.2/505.2
**Cyclic peptides**				
Compound	Sequence	Yield (%)[e]	Purity (%)[c]	ESI-MS [M+1]+ [d]
TH2	c[GQN-isoD]	62	97	415.4/415.4
TH3	c[GQN-(*R*)-isoD]	55	96	415.4/415.4
TH4	c[isoKSDL]	55	96	444.1/444.2
TH5	c[isoKSD-(*R*)-L]	47	95	444.1/444.2

[a] isoAsp corresponds to Asp-OBn. [b] based on the estimated loading of the resin. [c] Determined by analytical RP HPLC (General Methods). [d] Experimental/Expected. [e] Determined after semi-preparative RP HPLC.

Table ST2. RP HPLC and ESI MS analyses of the fully deprotected linear peptides. Reaction yields are also shown.

Protected peptides[a]		Fully deprotected peptides				
Compound	Sequence	Compound	Sequence	Yield (%)[e]	Purity (%)[c]	ESI-MS [M+1]+[d]
TH6	GQND-OBn	TH16	GQND	72	96	432.9/433.2
TH7	GQN-(*R*)-D-OBn	TH17	GQN-(*R*)-D	69	97	432.9/433.2
		TH1	GQNGISDL	85	97	803.3/803.4
TH8	Fmoc-KSD(OBn)L	TH18	KSDL	73	97	462.0/462.2
TH9	Fmoc-KSD-(*R*)-L	TH19	KSD-(*R*)-L	72	96	462.0/462.2

[a] isoAsp corresponds to Asp-OBn. [b] Determined after semi-preparative RP HPLC.
[c] Determined by analytical RP HPLC. [d] Experimental/Expected. [e]Determined after semi-preparative RP HPLC.

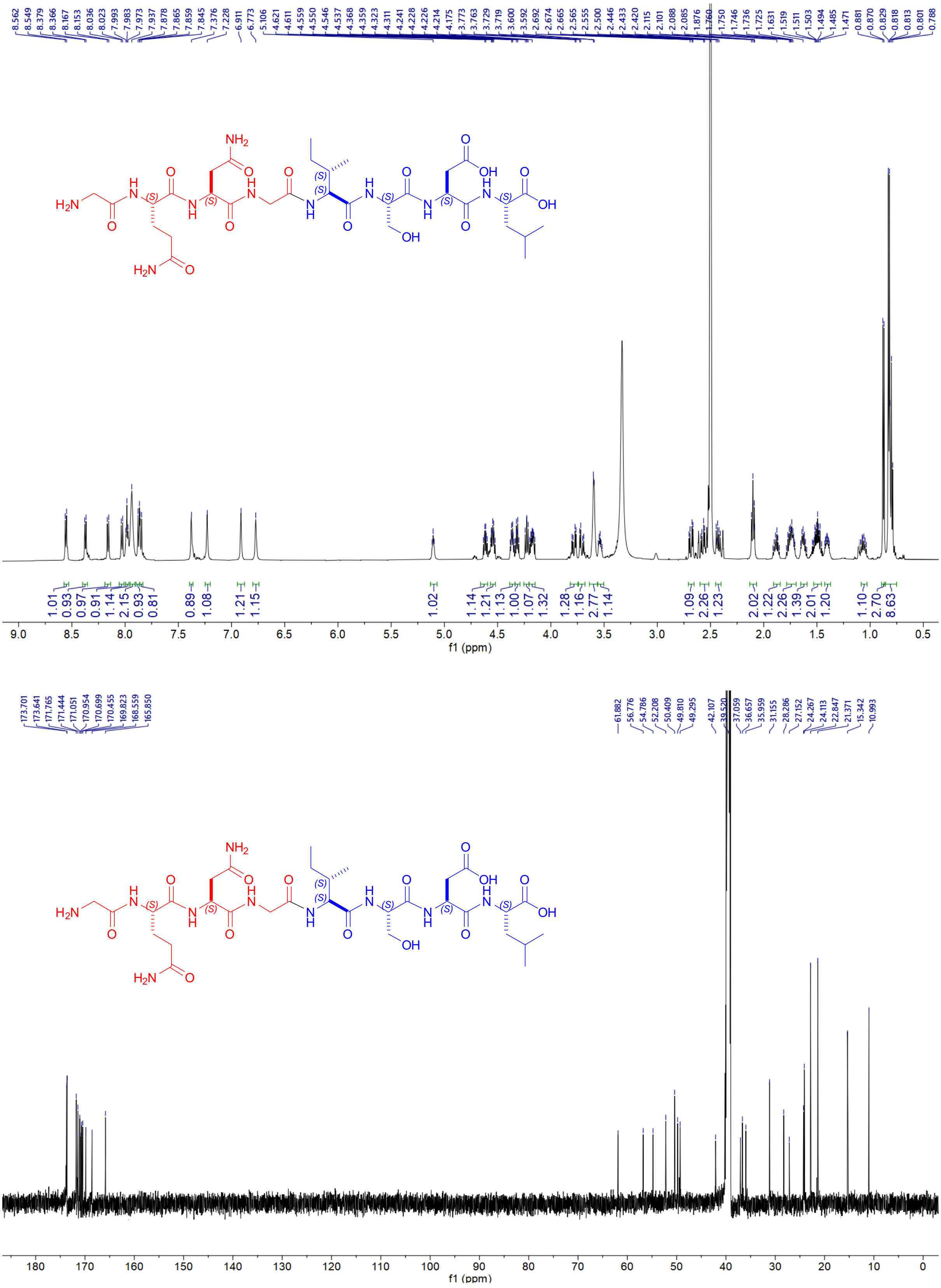

Supplementary Figure S5A. NMR spectra of the purified TH1 octapeptide.

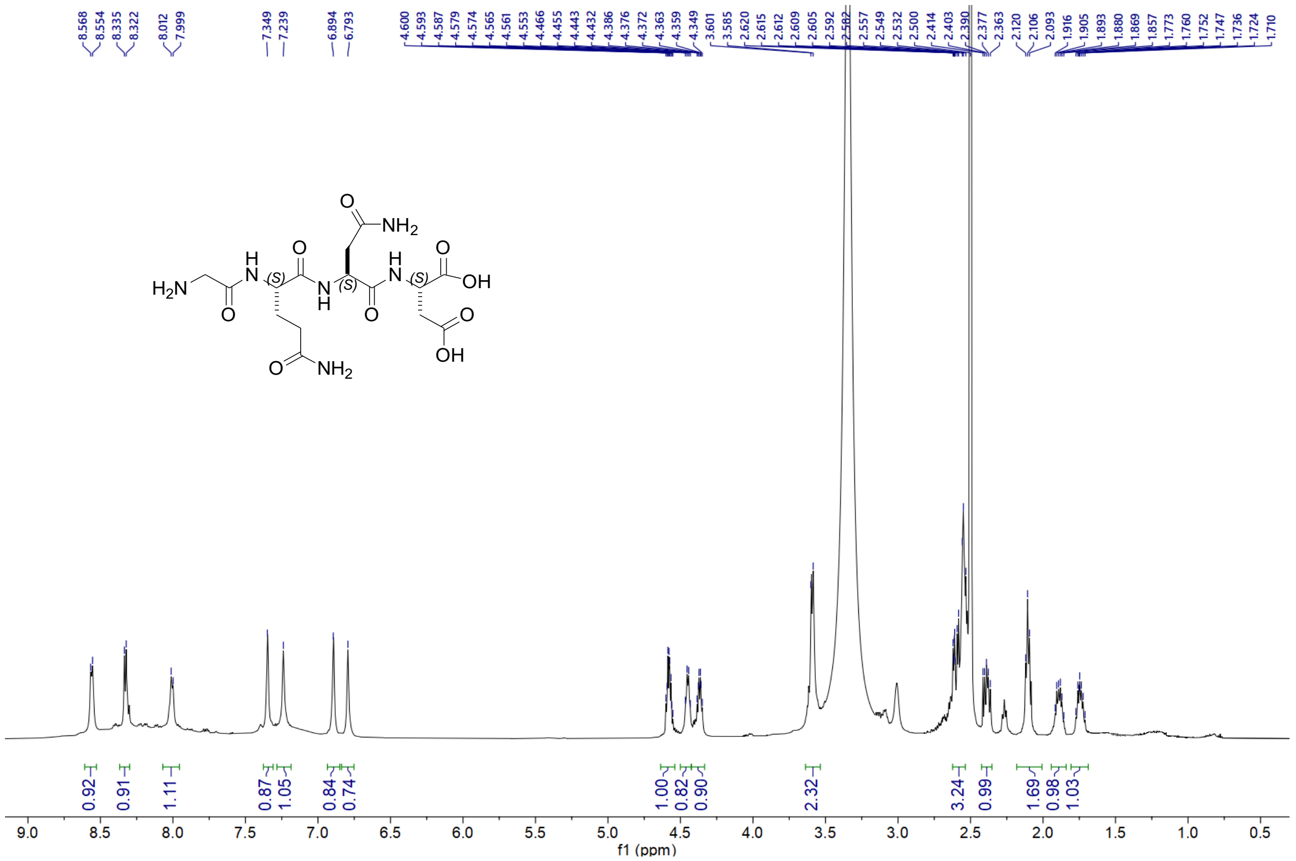

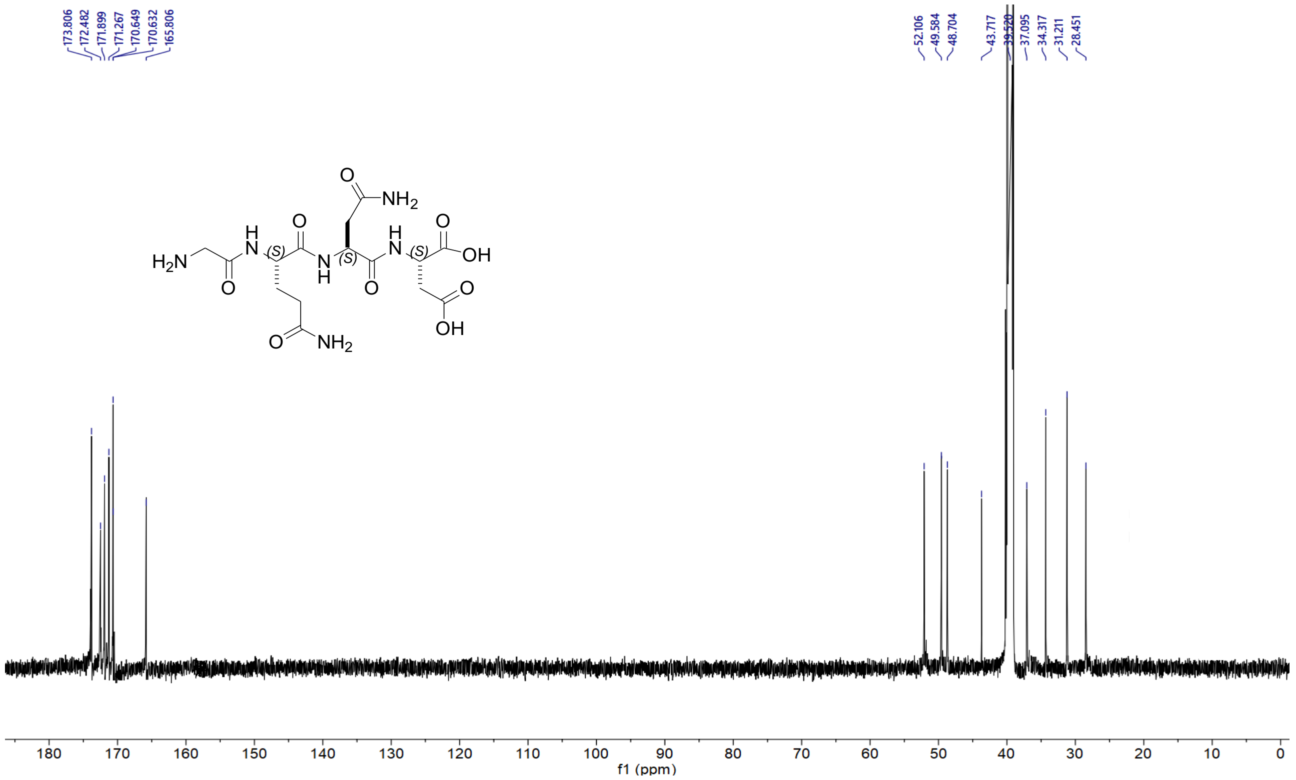

Supplementary Figure S5B. NMR spectra of the purified TH16 tetrapeptide.

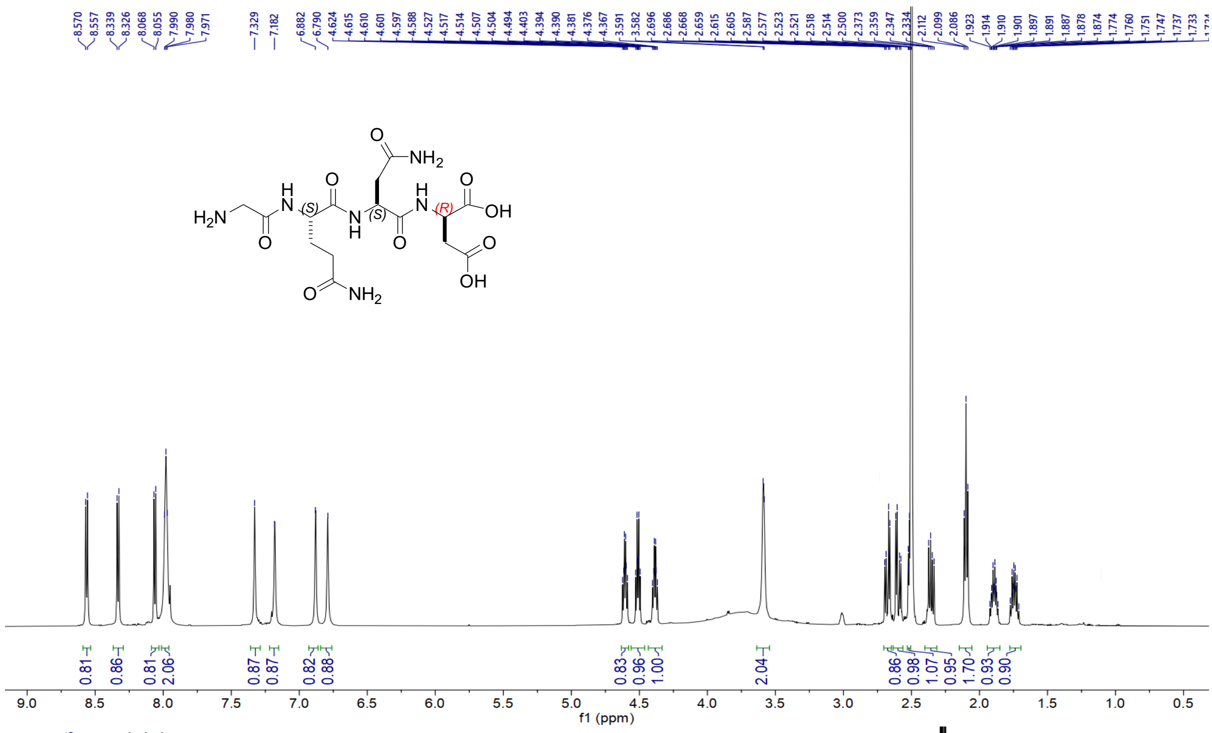

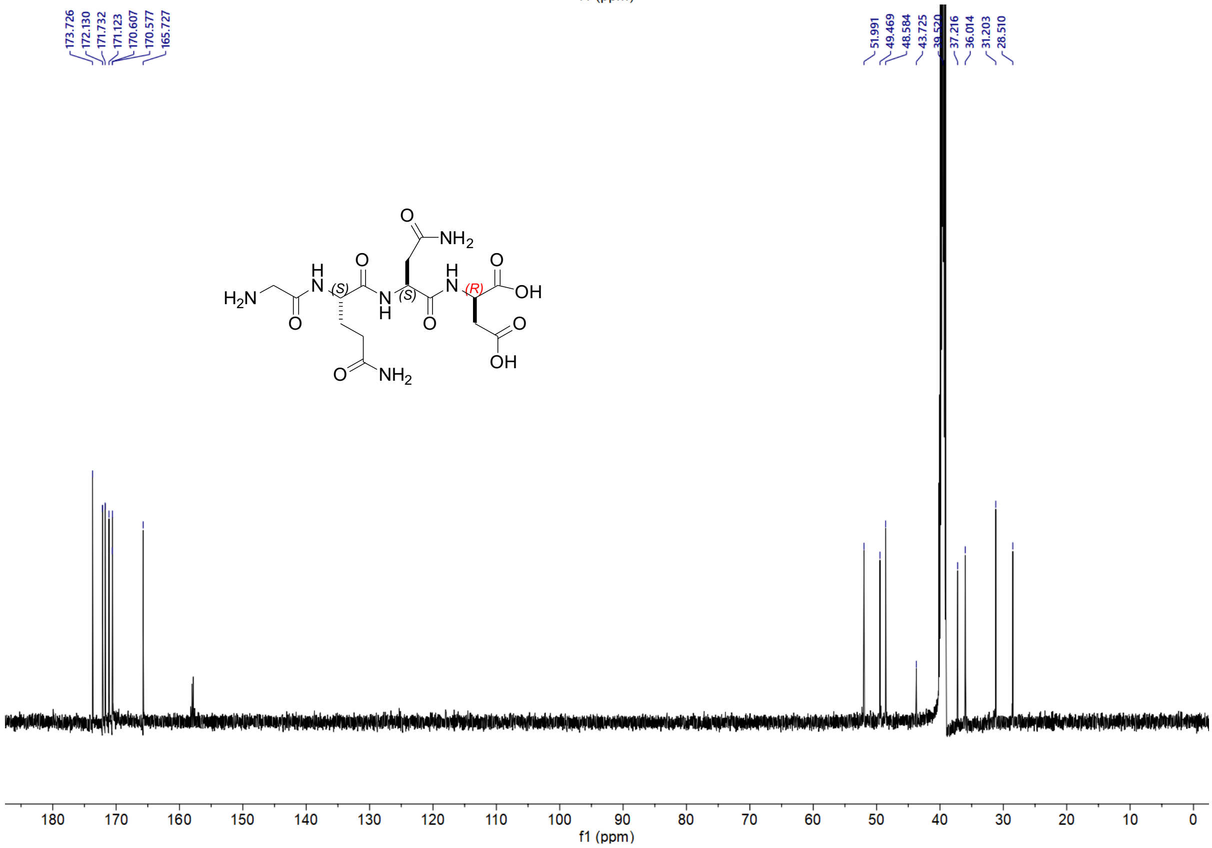

Supplementary Figure S5C. NMR spectra of the purified TH17 tetrapeptide.

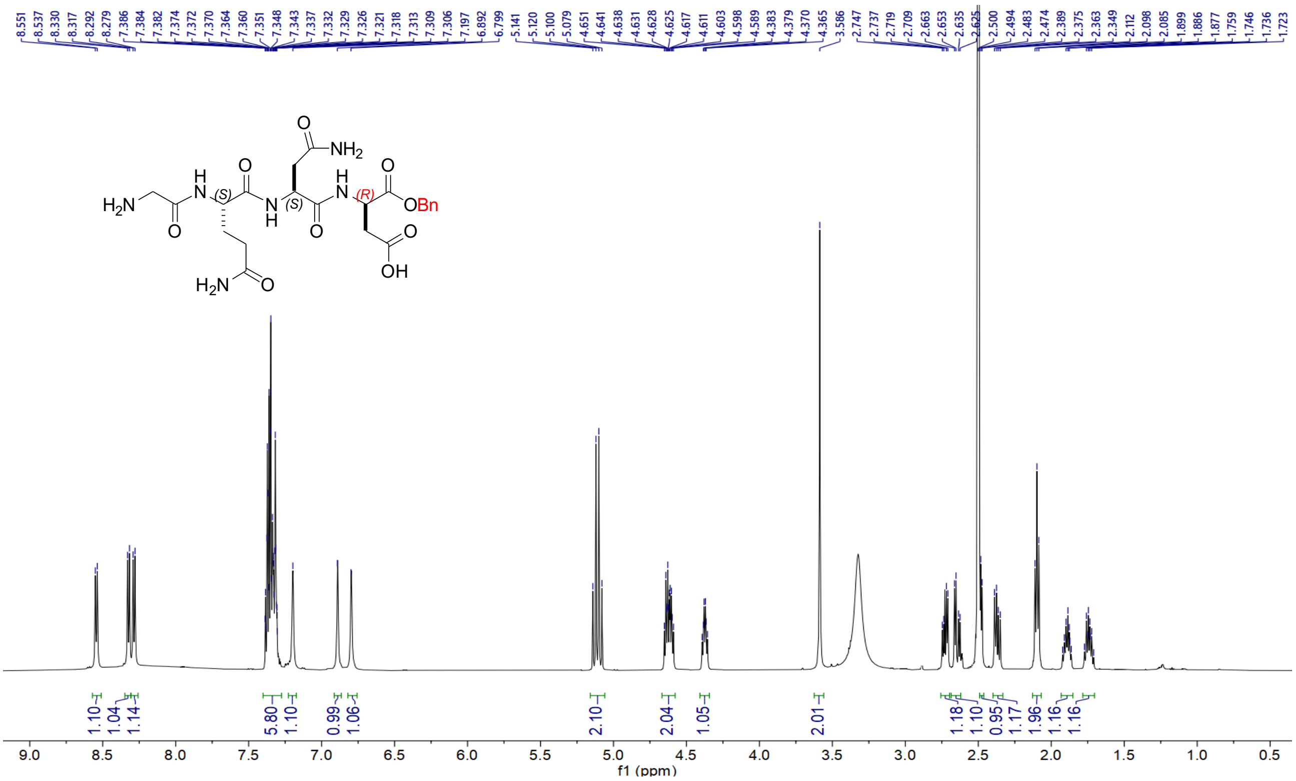

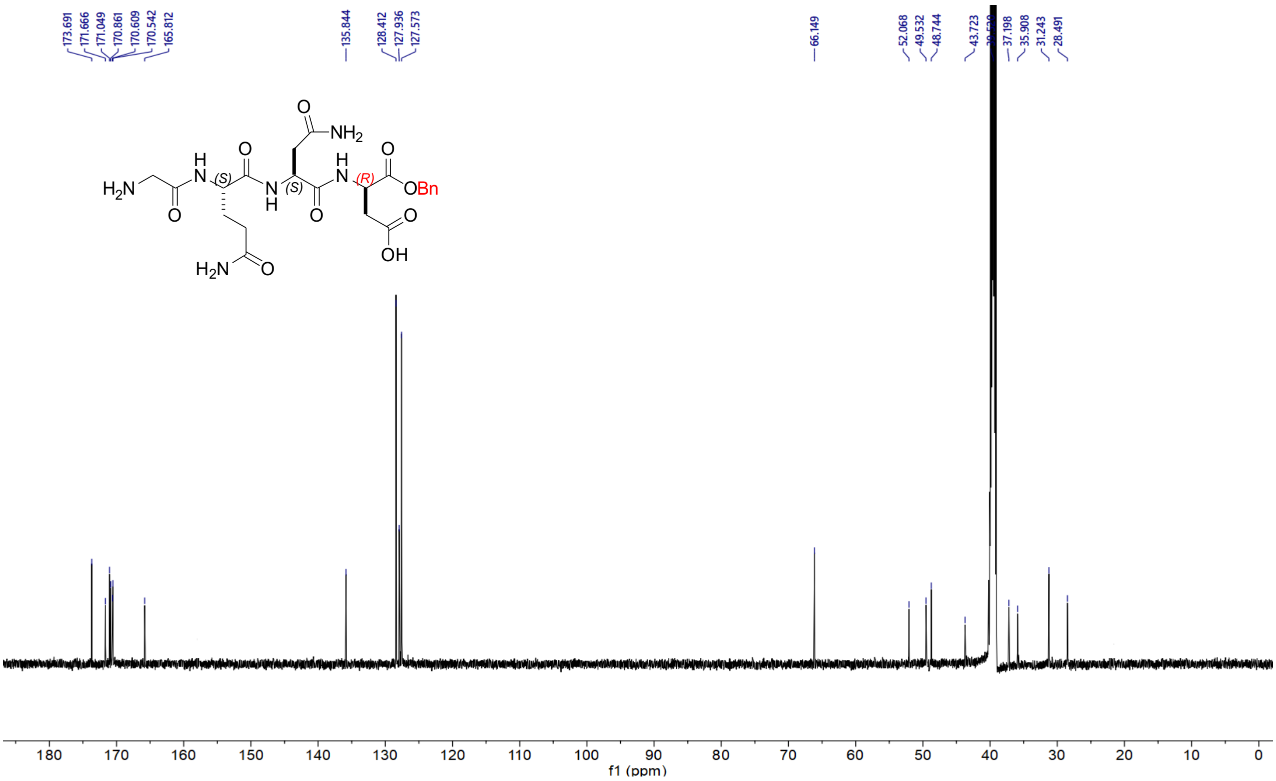

Supplementary Figure S5D. NMR spectra of the purified TH7 tetrapeptide.

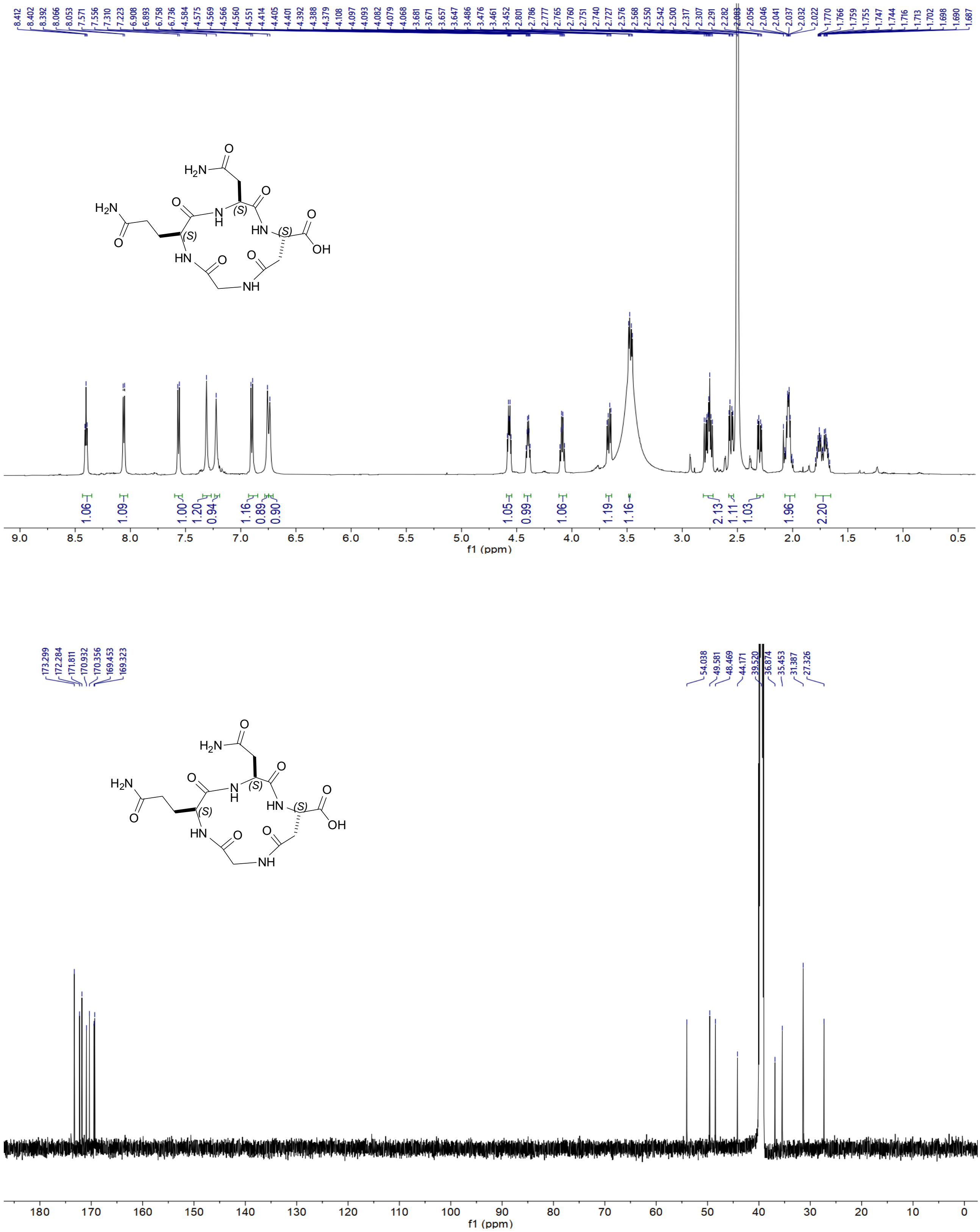

Supplementary Figure S5E. NMR spectra of the purified TH2 tetrapeptide.

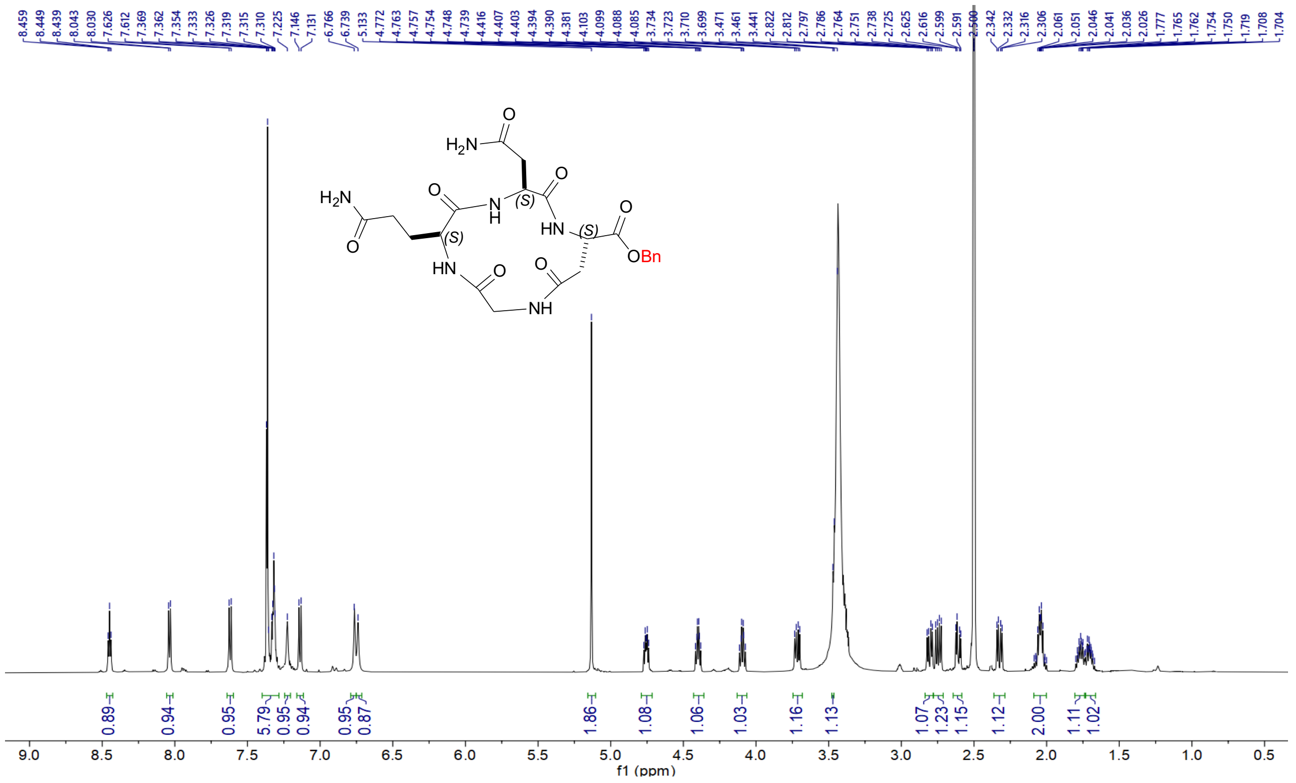

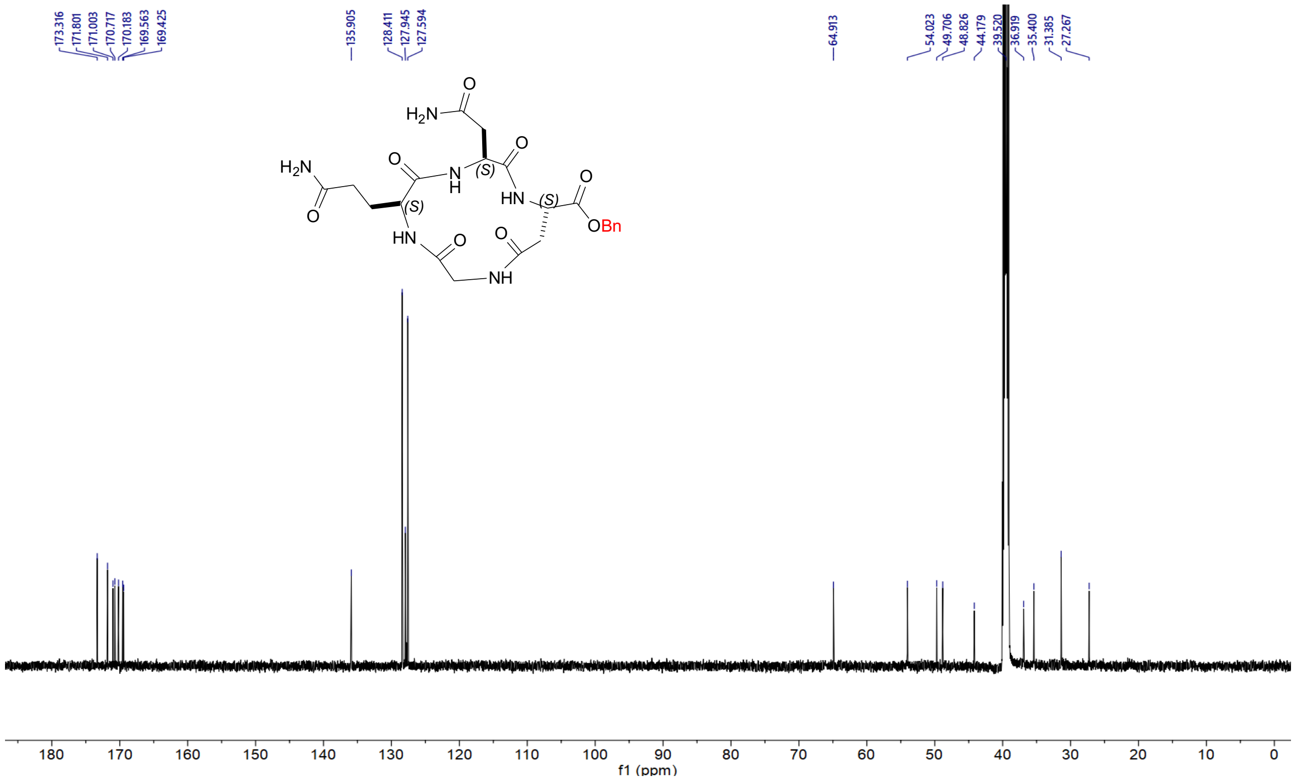

Supplementary Figure S5F. NMR spectra of the purified TH10 tetrapeptide.

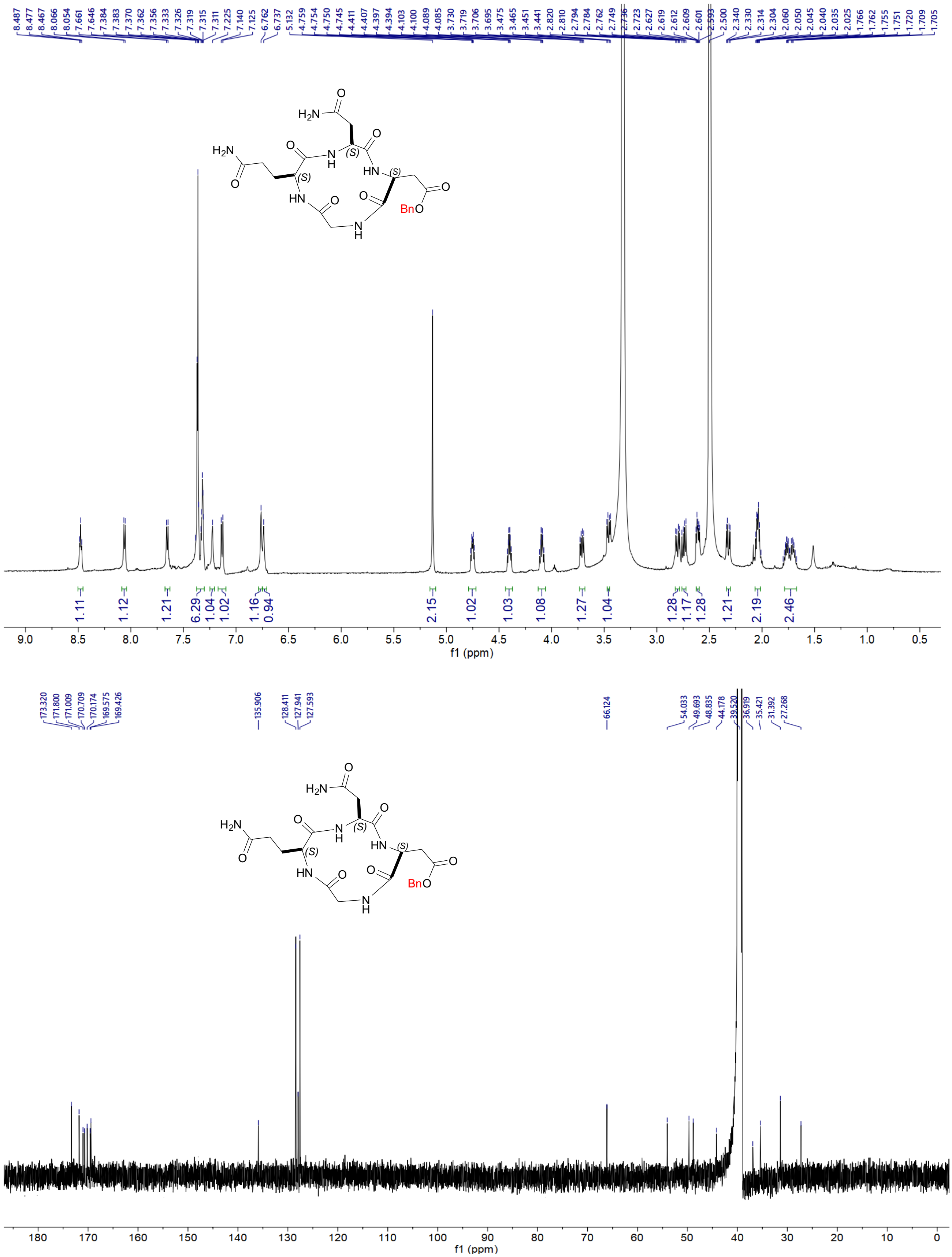

Supplementary Figure S5G. NMR spectra of the purified TH15 tetrapeptide.

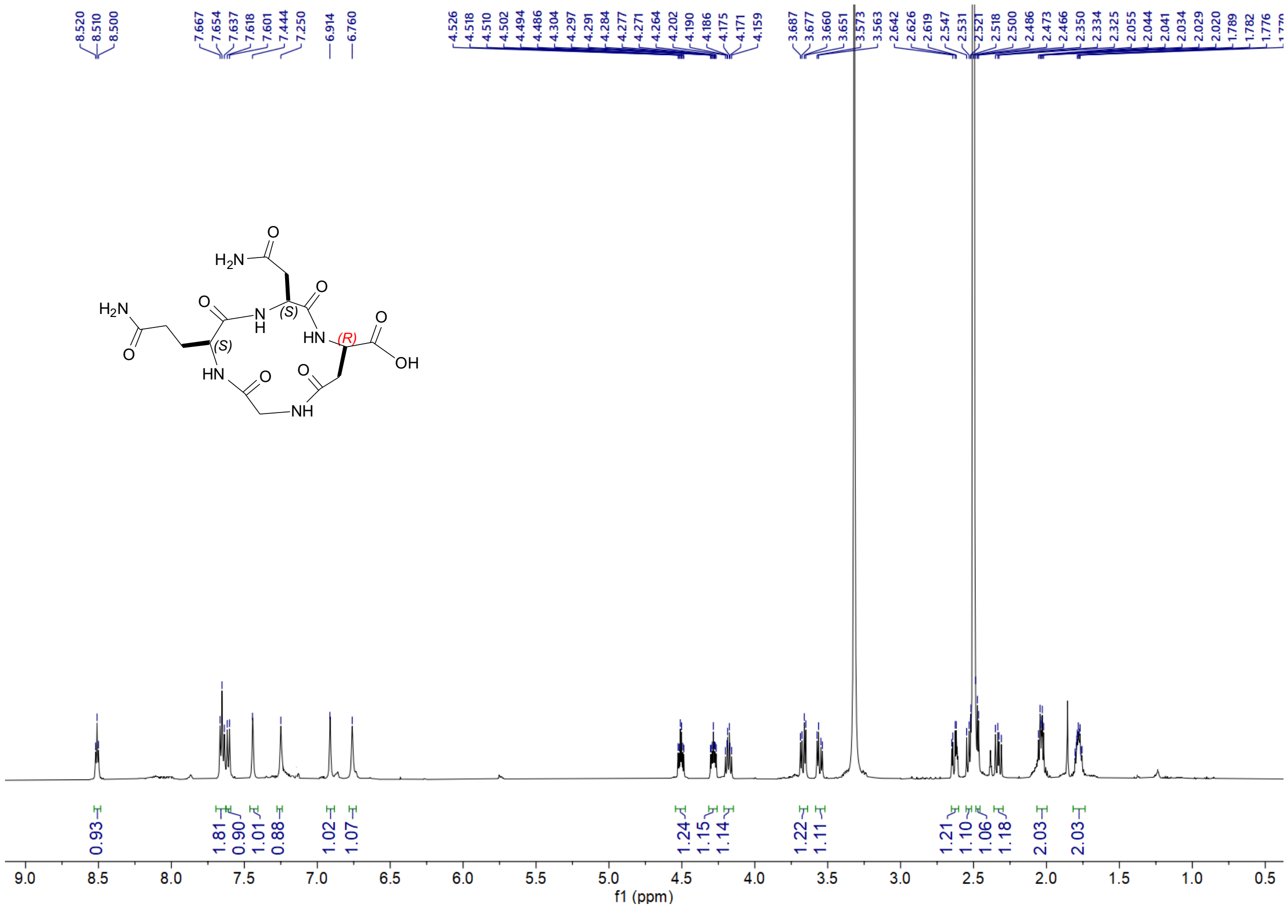

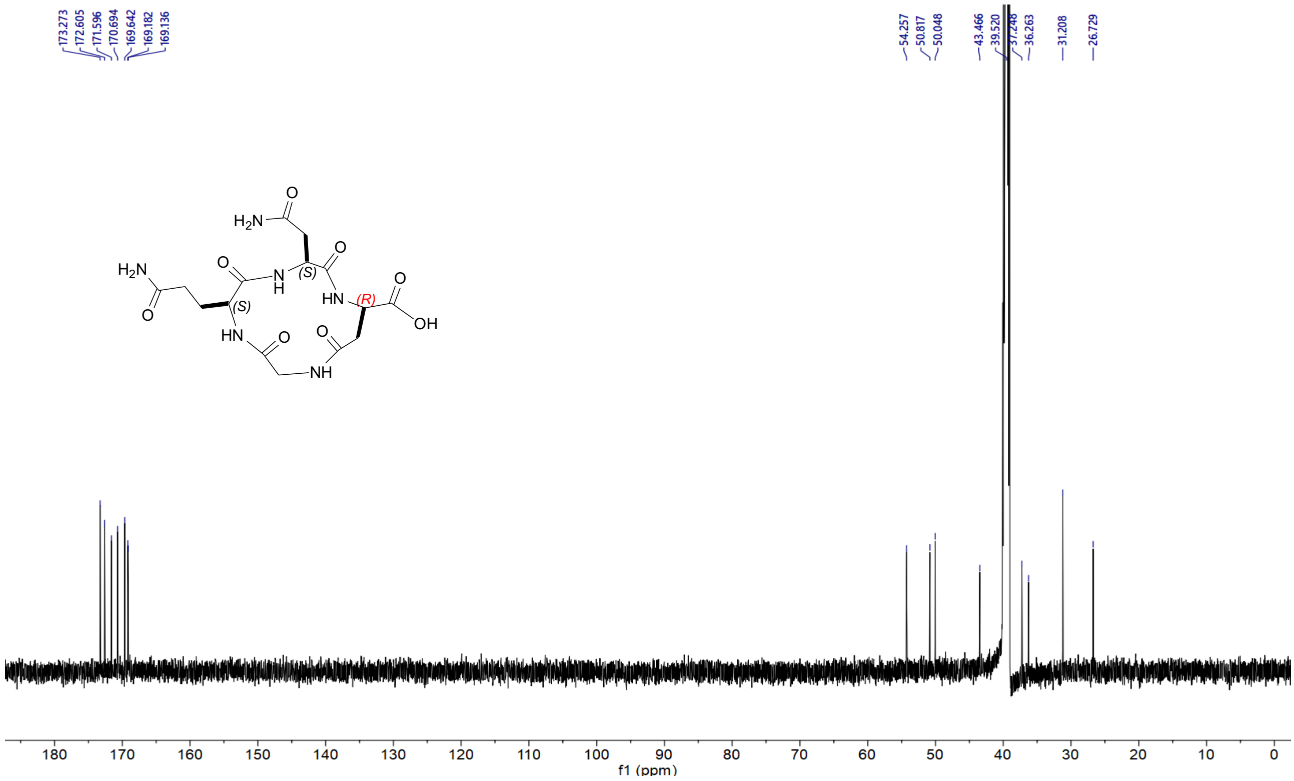

Supplementary Figure S5H. NMR spectra of the purified TH3 tetrapeptide.

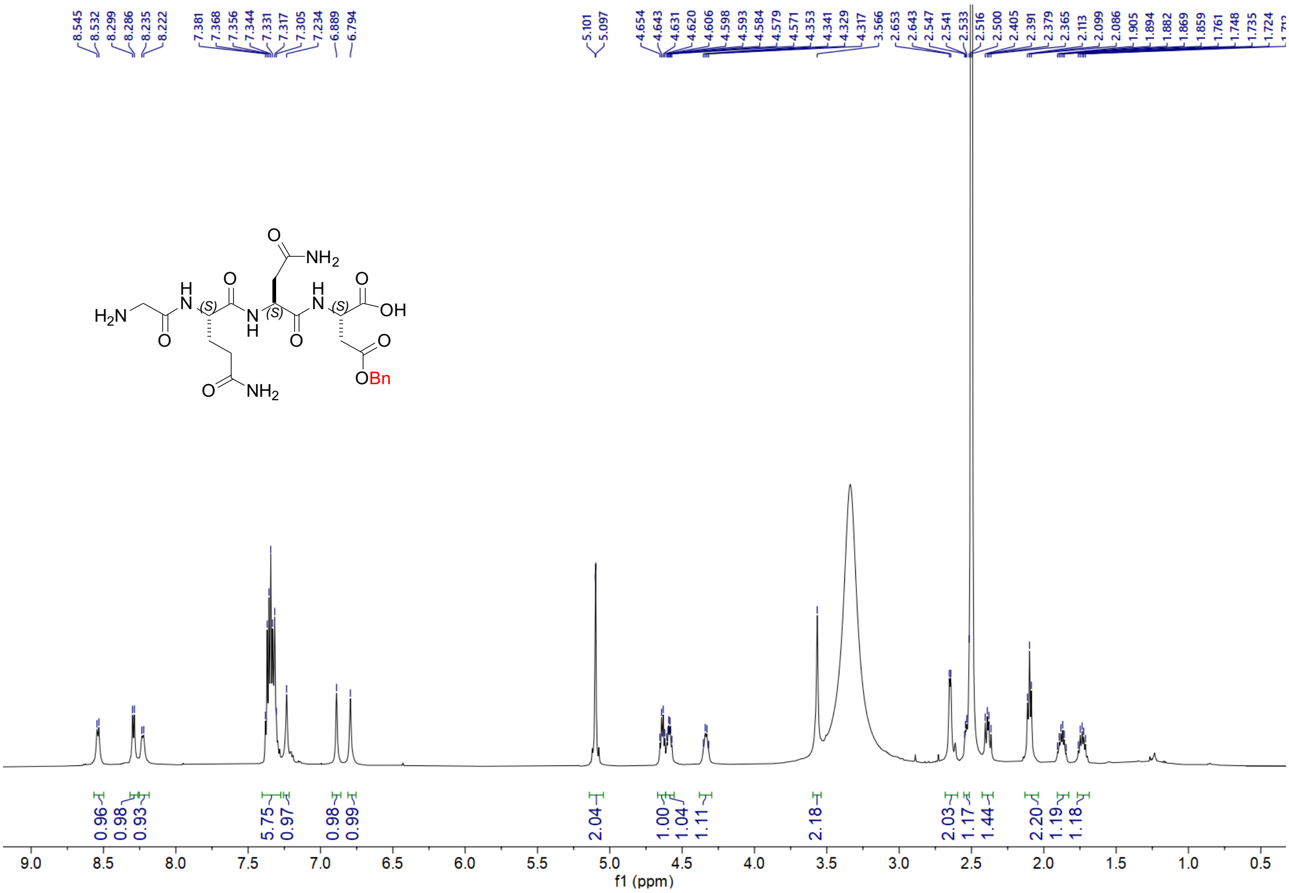

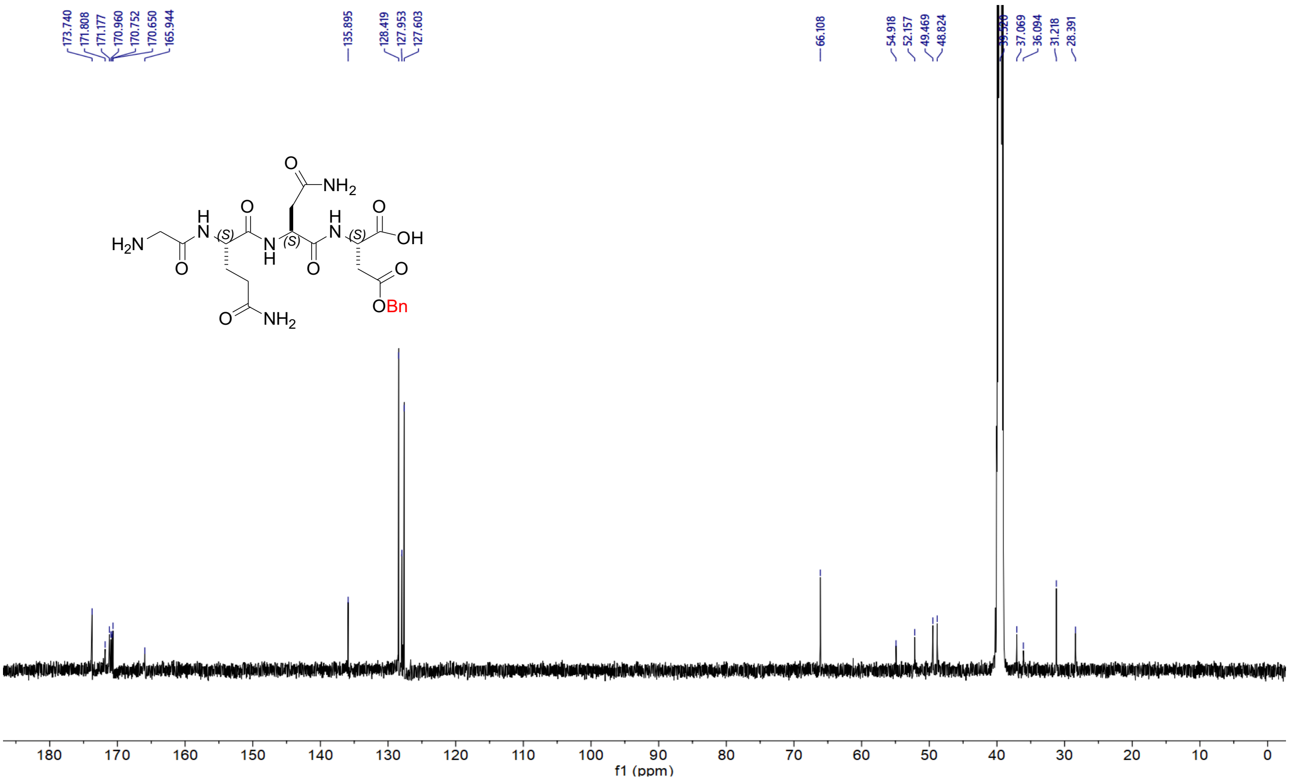

Supplementary Figure S5I. NMR spectra of the purified TH14 tetrapeptide.

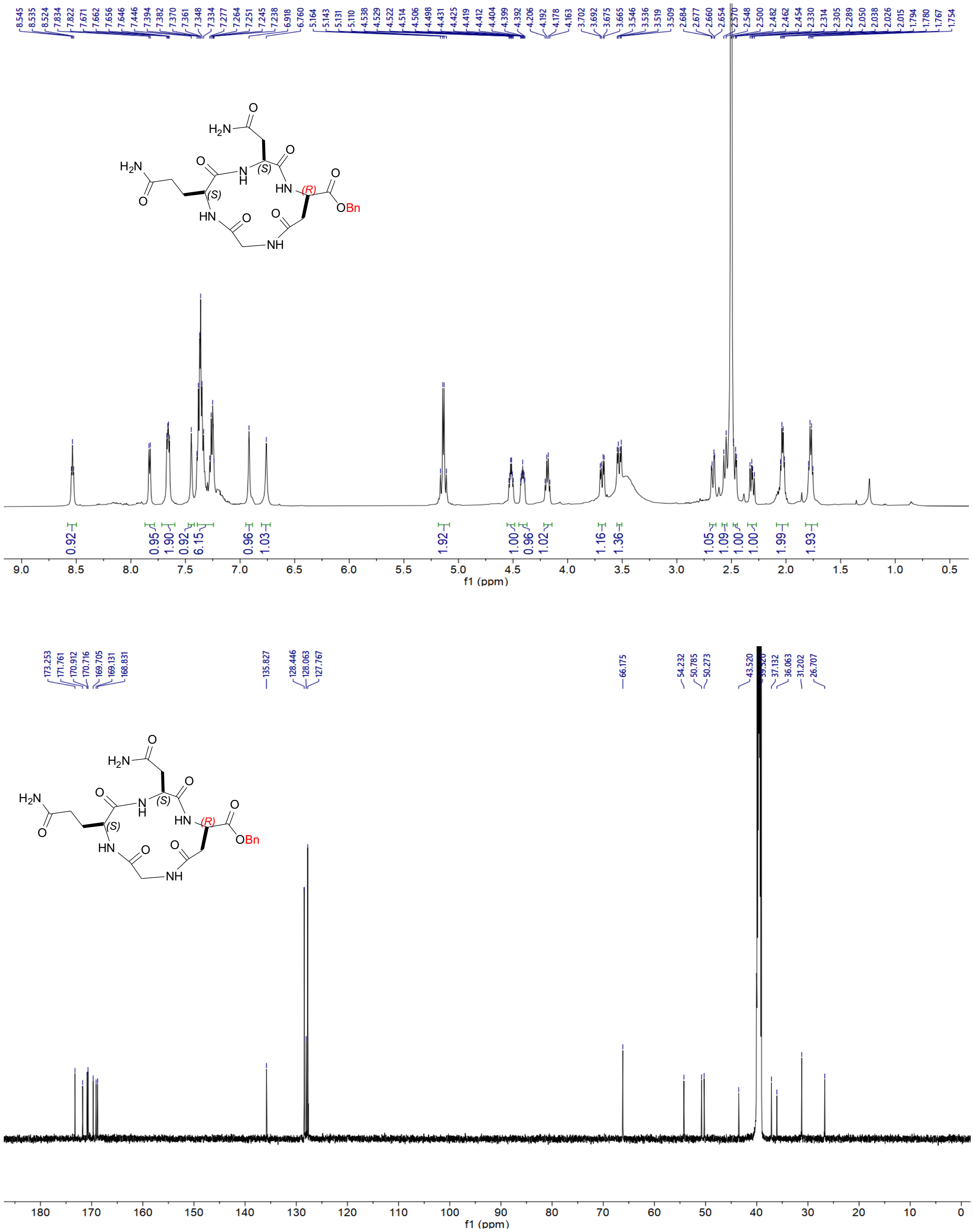

Supplementary Figure S5L. NMR spectra of the purified TH11 tetrapeptide.

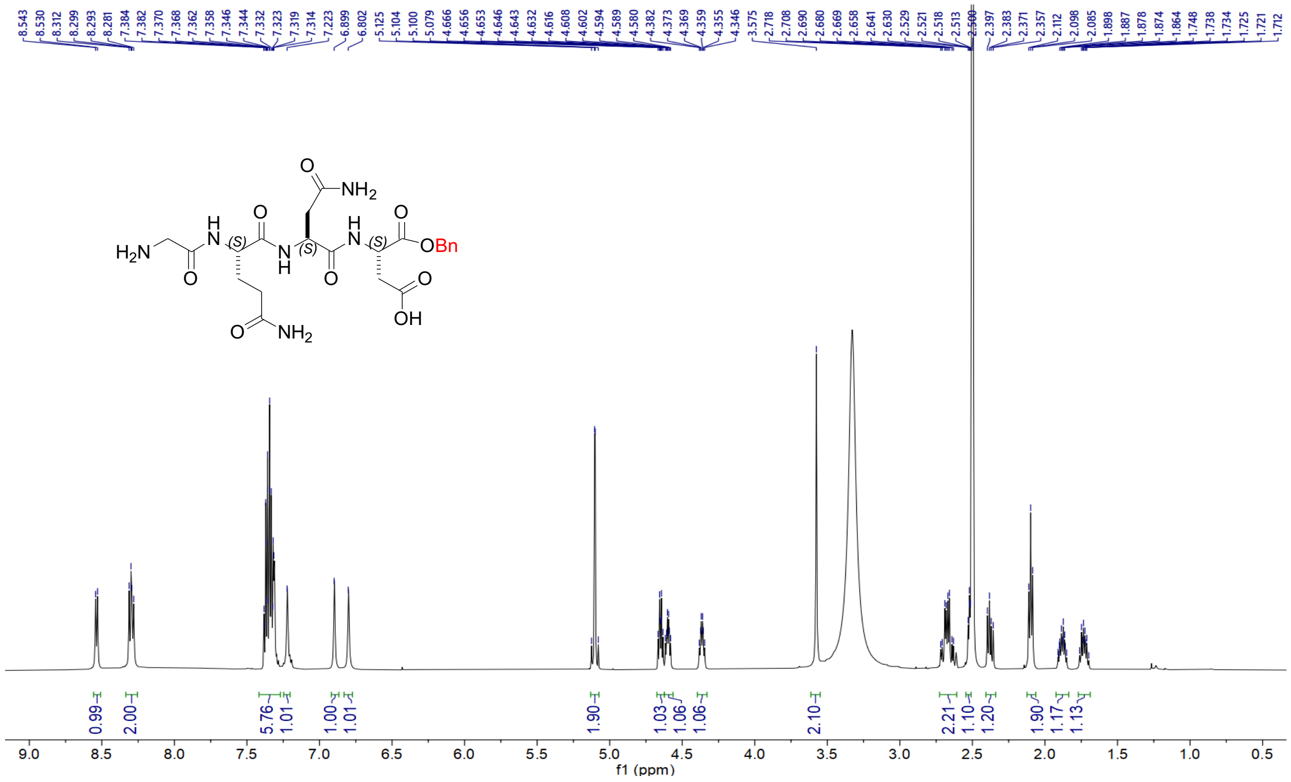

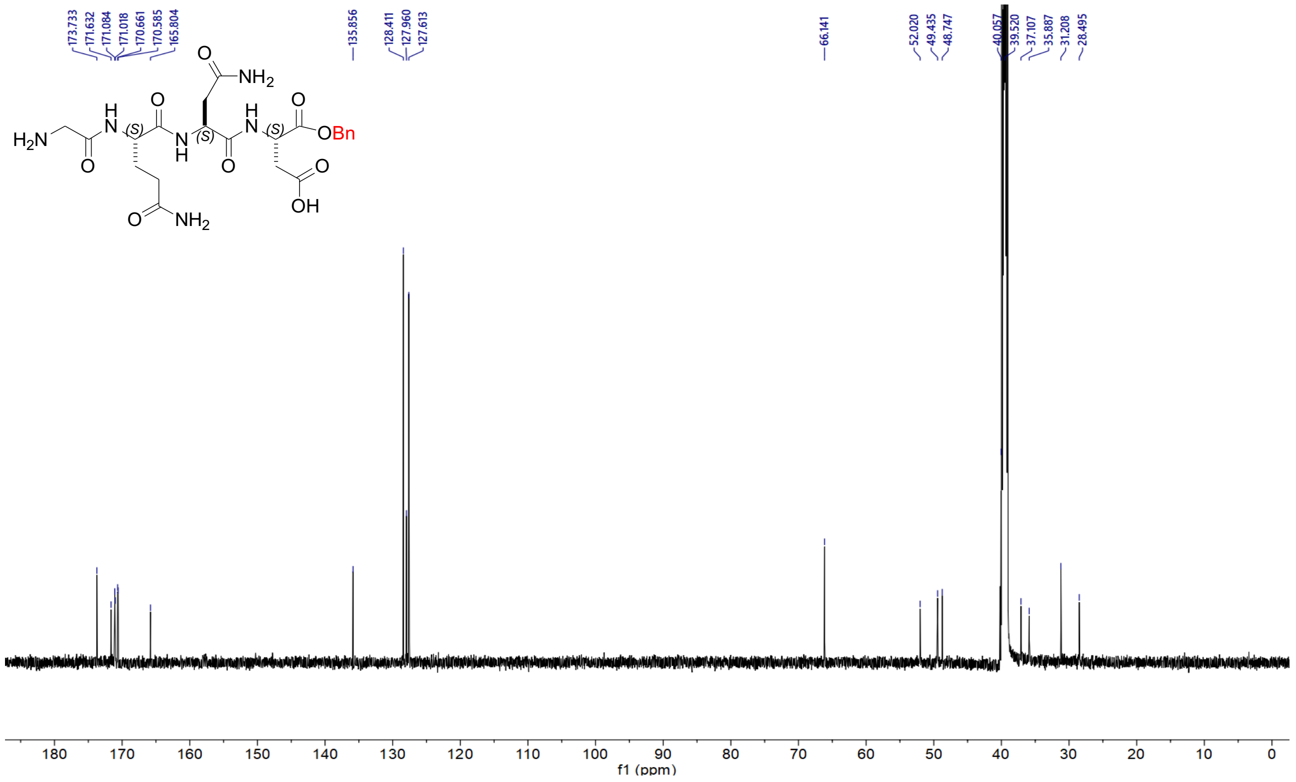

Supplementary Figure S5M. NMR spectra of the purified TH6 tetrapeptide.

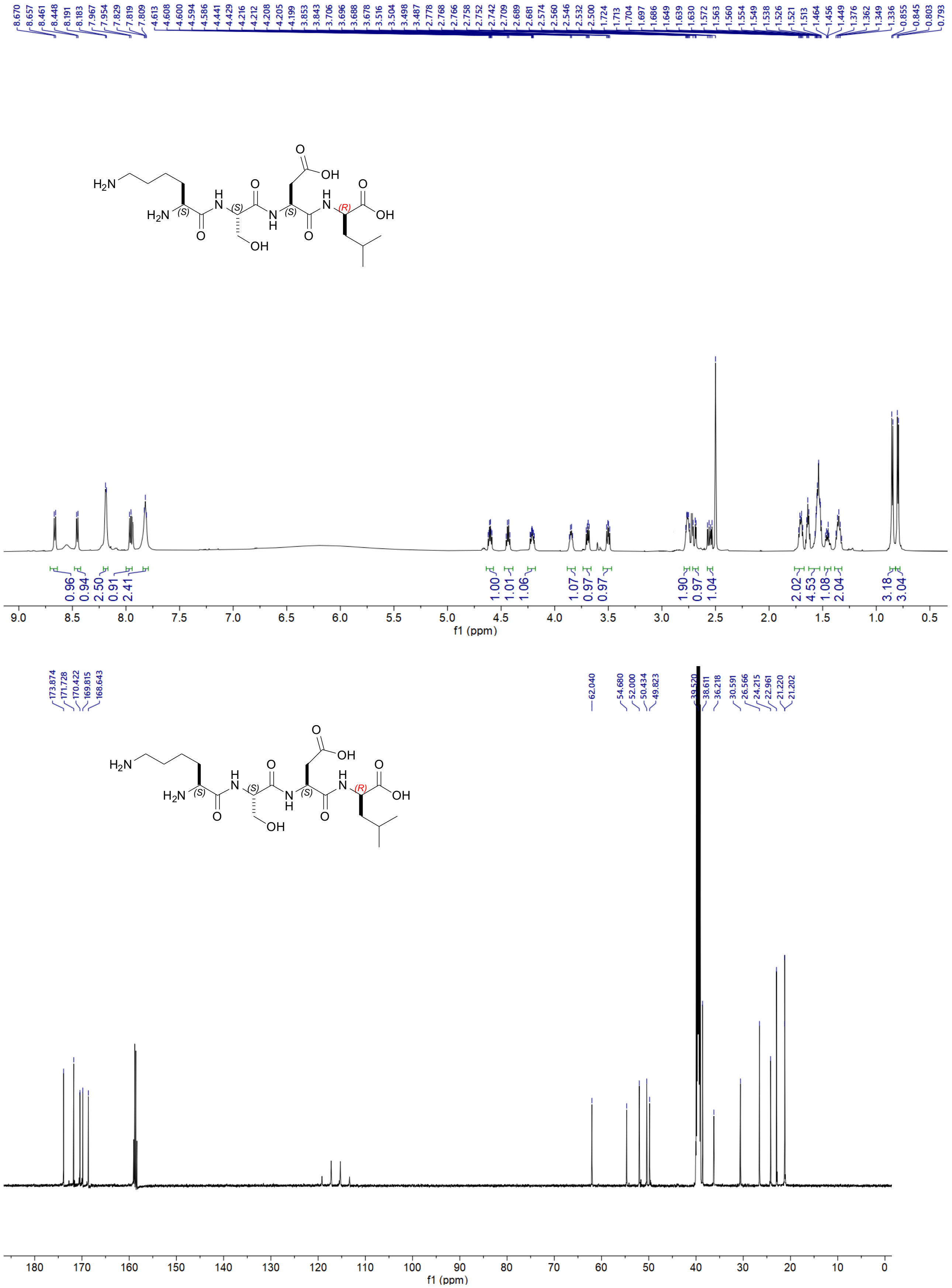

Supplementary Figure S5N. NMR spectra of the purified TH19 tetrapeptide.

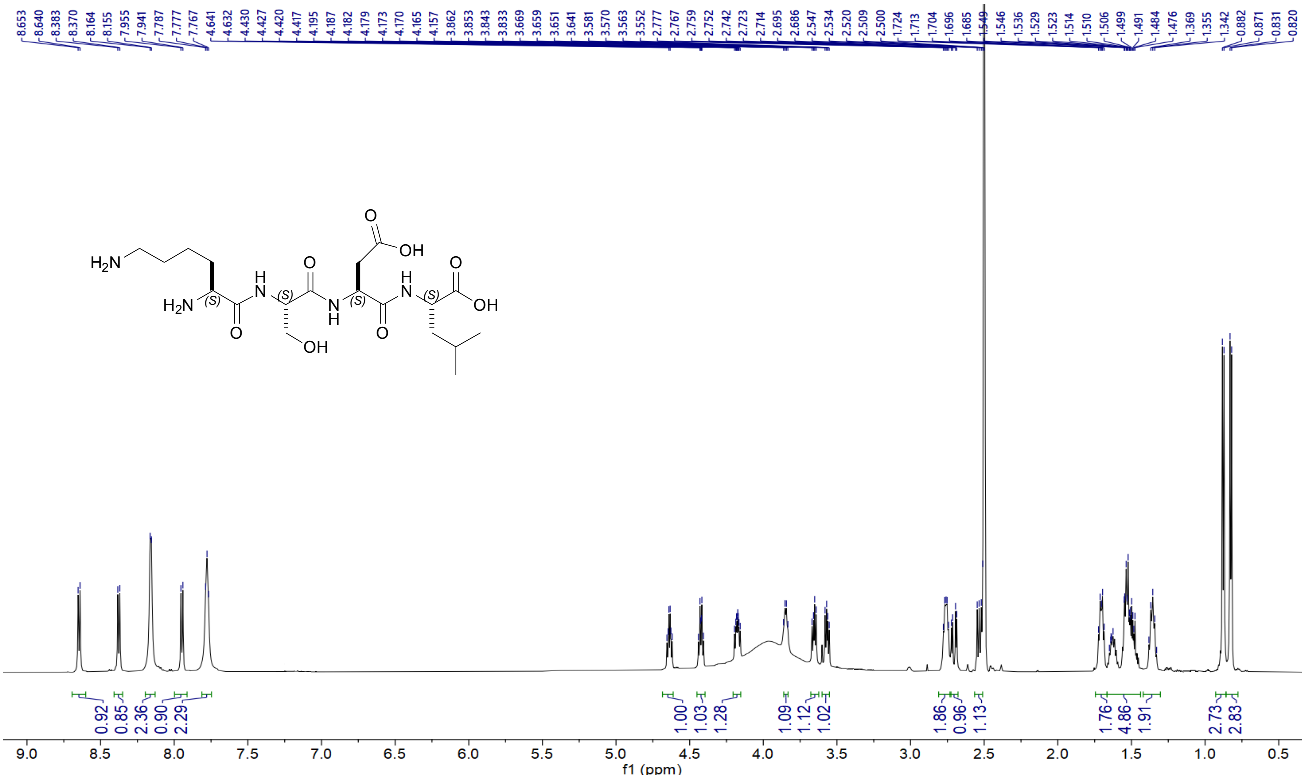

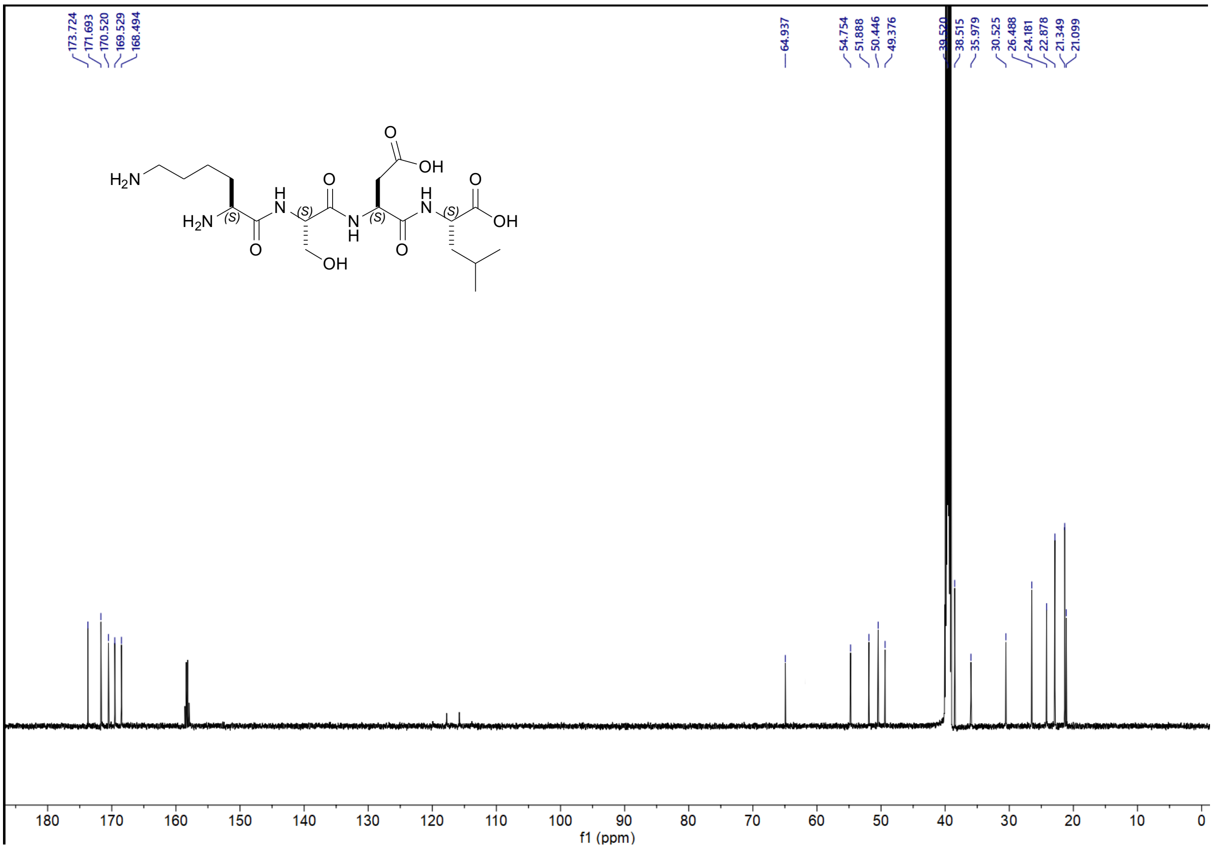

Supplementary Figure S5O. NMR spectra of the purified TH18 tetrapeptide.

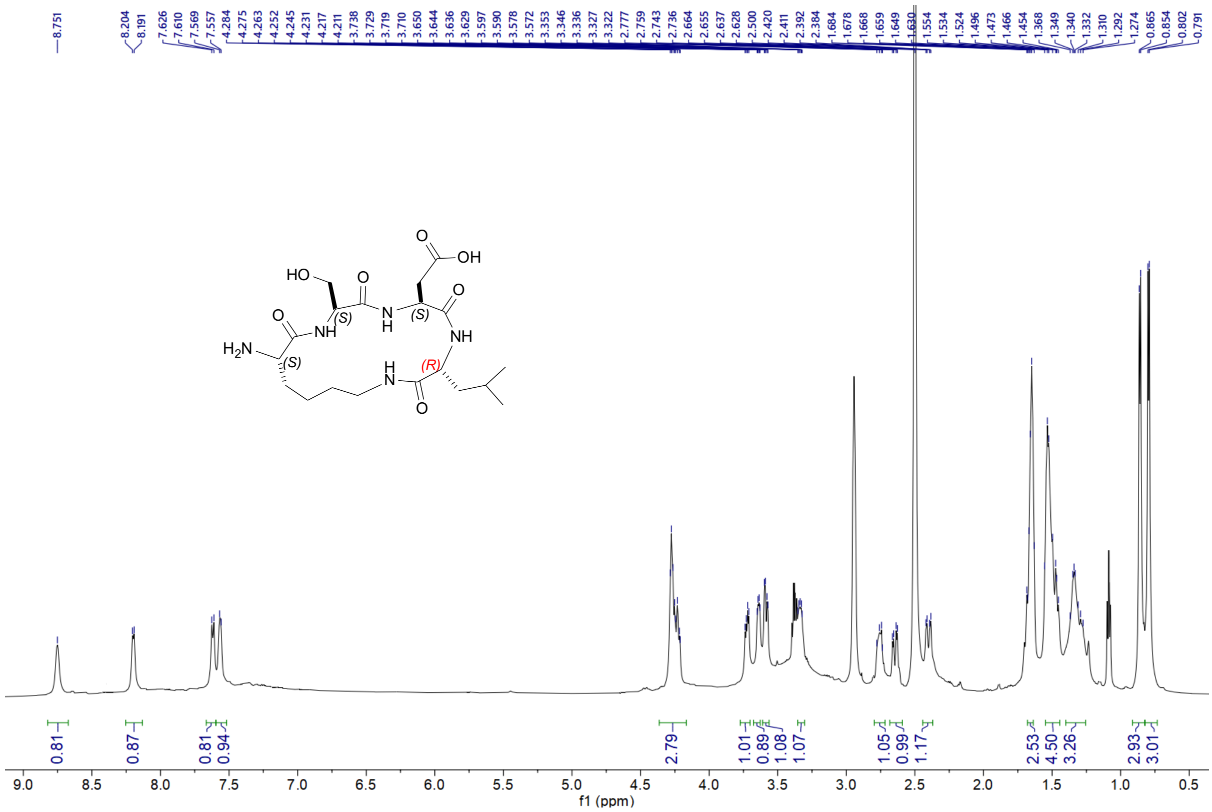

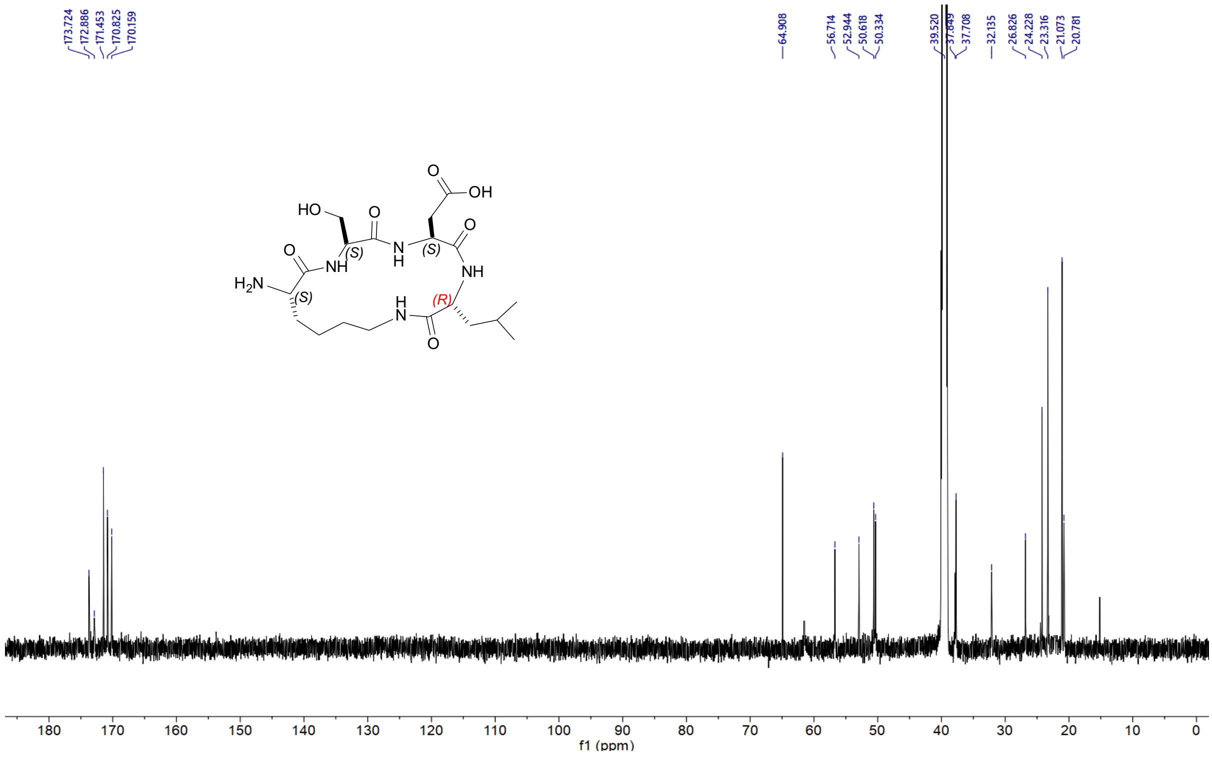

Supplementary Figure S5P. NMR spectra of the purified tetrapeptide TH5.

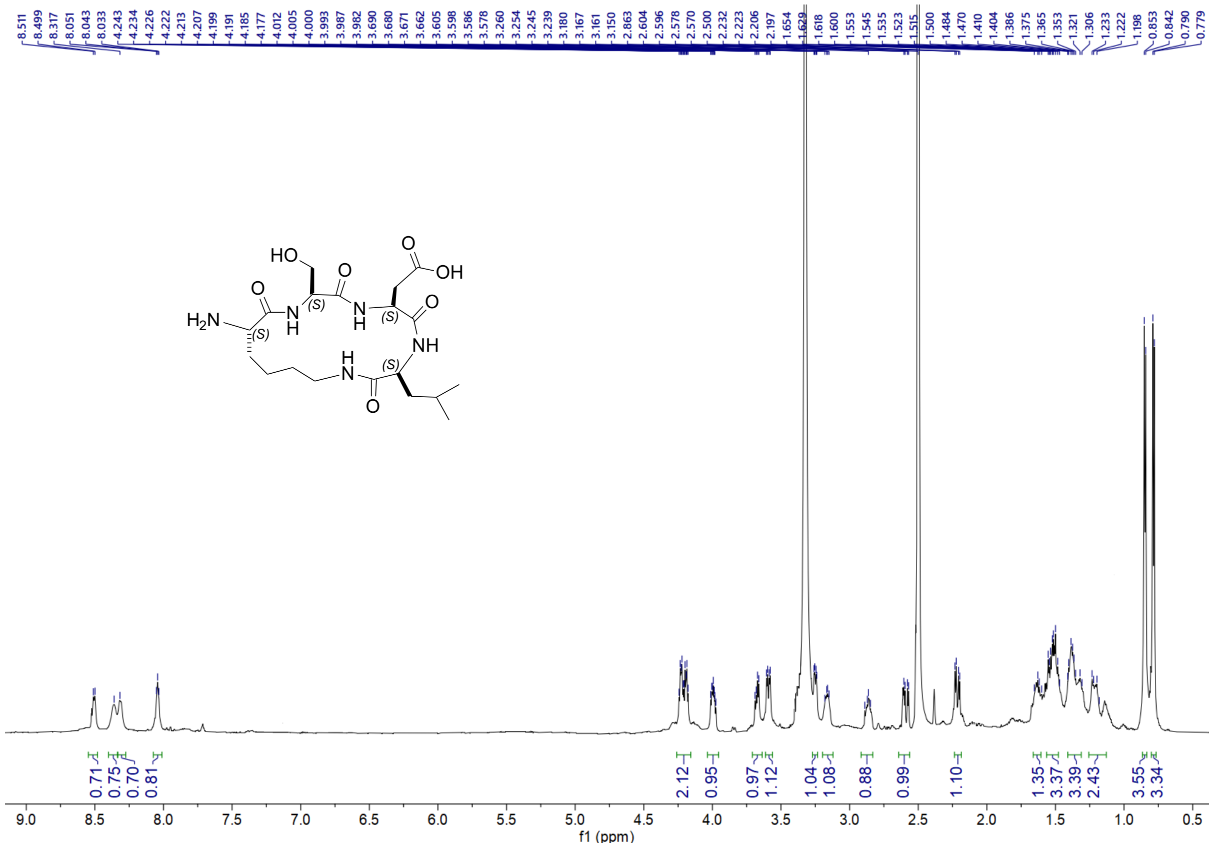

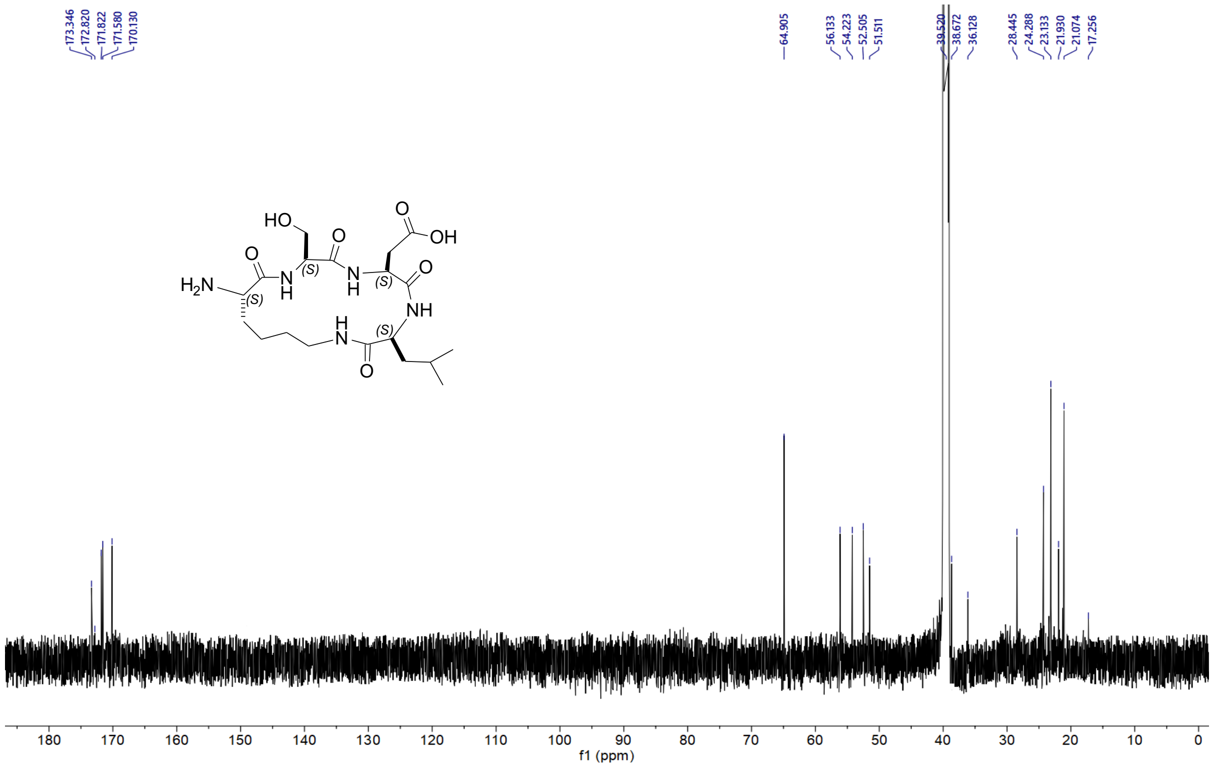

Supplementary Figure S5Q. NMR spectra of the purified tetrapeptide TH4.

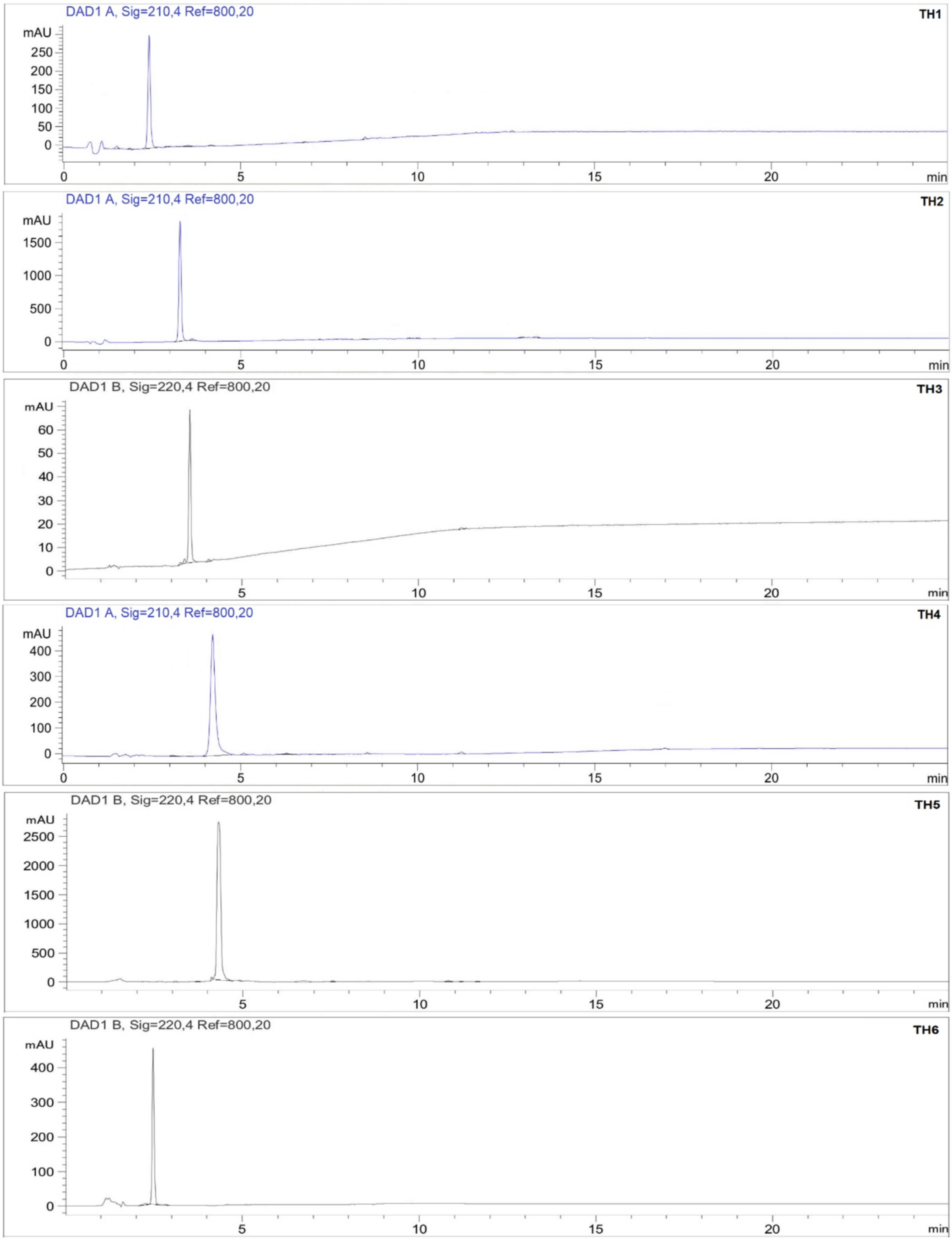

Supplementary Figure S6A. Analytical HPLC chromatograms of the indicated peptides.

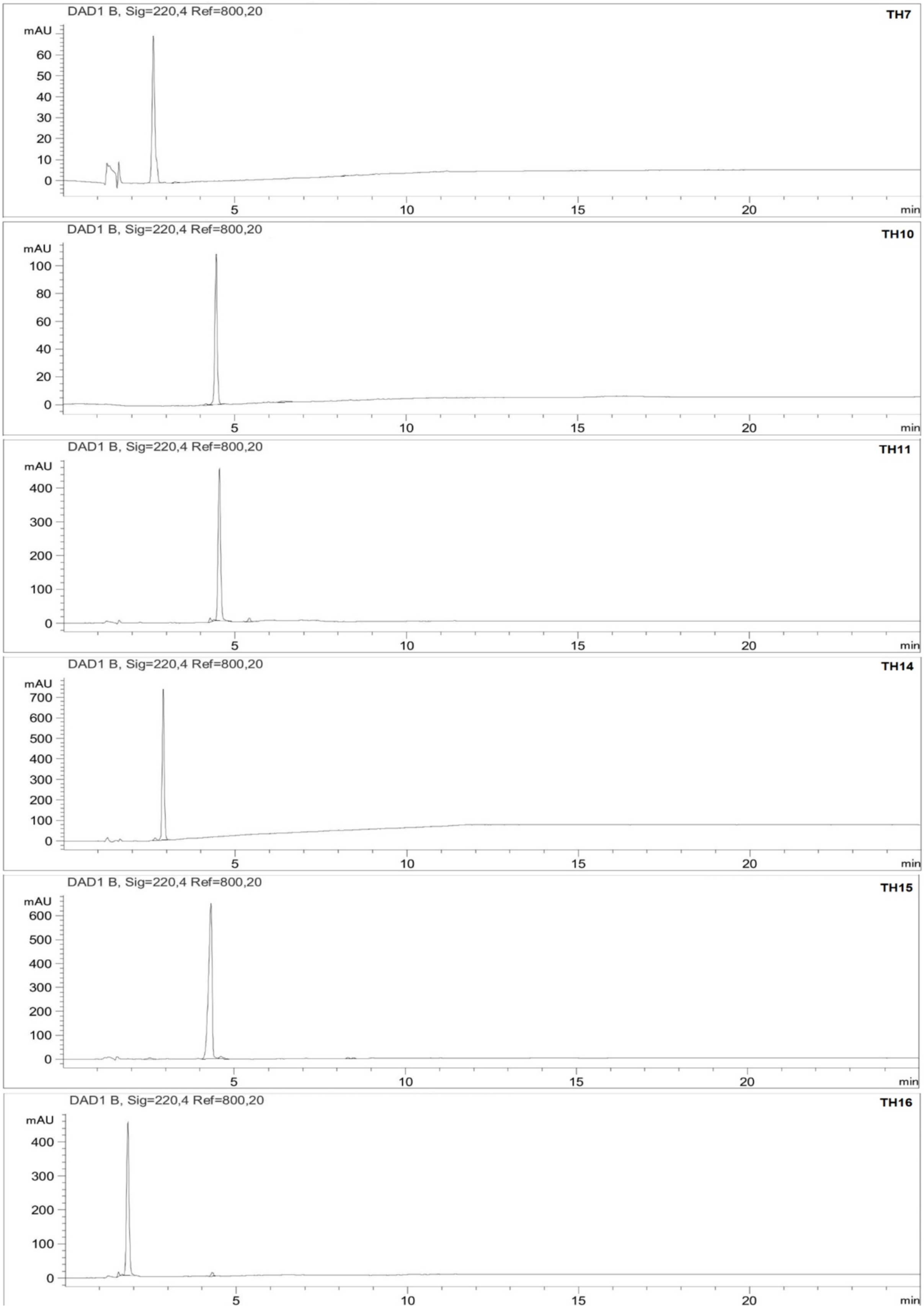

Supplementary Figure S6B. Analytical HPLC chromatograms of the indicated peptides.

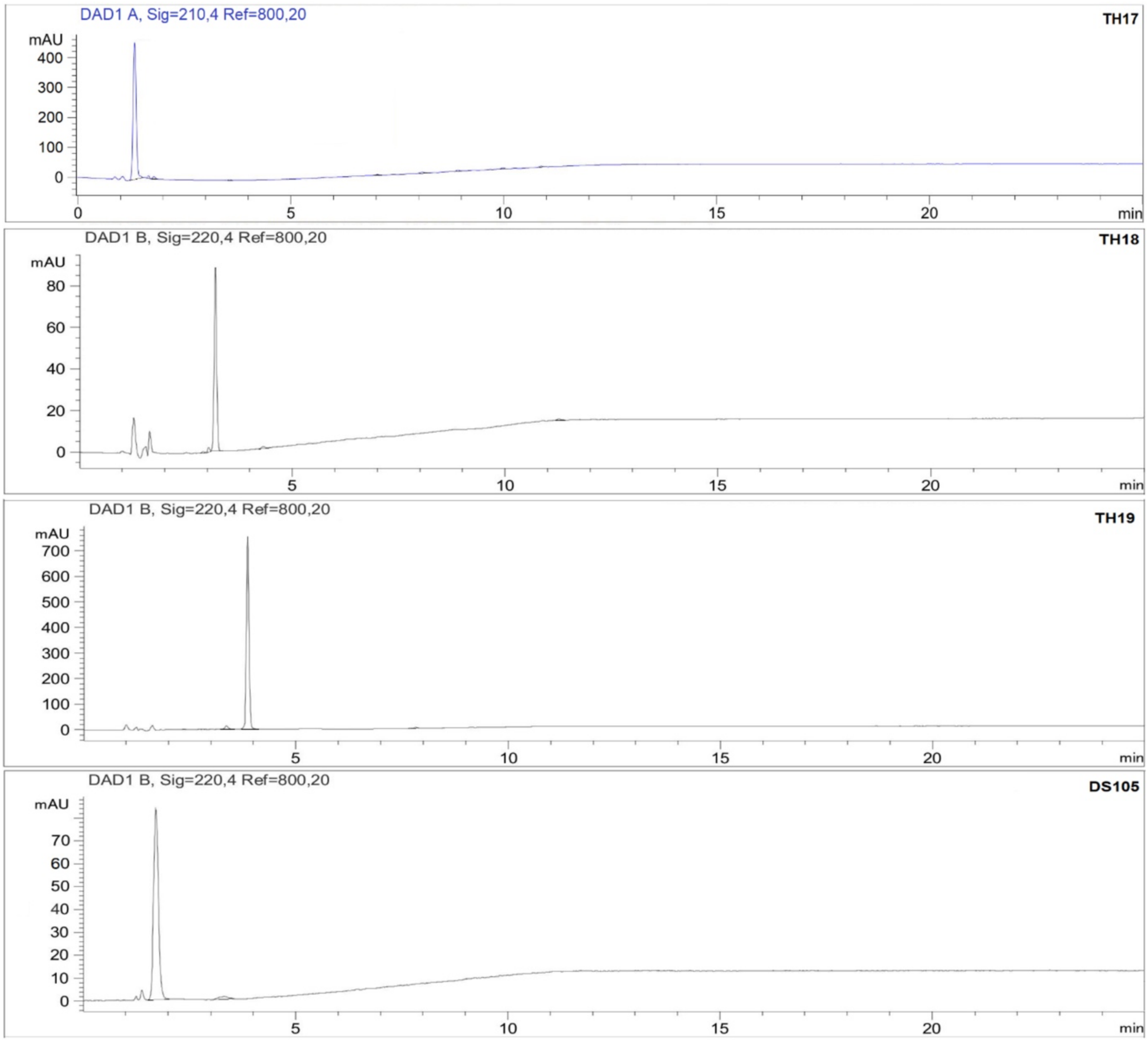

Supplementary Figure S6C. Analytical HPLC chromatograms of the indicated peptides.
